# Stirring Without Stirrers: Polymer Fouling‐Driven Mass Transport Unlocks Order‐of‐Magnitude Gain in Electrochemiluminescence

**DOI:** 10.1002/advs.202506610

**Published:** 2025-07-16

**Authors:** Wathsala Prasadini Kapuralage, Hemendra Kala, Mariusz Martyniuk, Nadim Darwish, Melanie MacGregor, K. Swaminathan Iyer, Simone Ciampi

**Affiliations:** ^1^ School of Molecular and Life Sciences Curtin University Bentley Western Australia 6102 Australia; ^2^ Department of Electrical Electronic and Computer Engineering The University of Western Australia Crawley Western Australia 6009 Australia; ^3^ Australian Research Council Centre for Transformative Meta‐Optical Systems The University of Western Australia Crawley Western Australia 6009 Australia; ^4^ Flinders Institute for Nanoscale Science and Technology Flinders University Bedford Park South Australia 5042 Australia; ^5^ School of Molecular Sciences The University of Western Australia Crawley Western Australia 6009 Australia

**Keywords:** convection, electrochemiluminescence, electroosmotic flow, Marangoni effect, photolithography

## Abstract

Electrode reactions are central to analytical chemistry and a green approach to chemical synthesis. Here, it is demonstrated that sacrificing electrode‐electrolyte contact to microscale polymeric blocks creates fouled electrodes that outperform unobstructed ones. By tuning the dielectric's geometry, surface chemistry, and charge – and controlling electrode alignment relative to gravity – a paradigm shift in electrode design, from “clean” to “fouled,” as a strategy to enhance reaction rates is proposed. Electrochemiluminescence (ECL) microscopy reveals that strategic electrode fouling enhances mass transport, primarily through electrochemically actuated lateral density gradients. Engineered fouling induces flow velocities up to 0.4 cm s^−1^ in otherwise quiescent systems. Sub‐millimeter plastic features boost local rates by up to 290%, while micrometer‐scale arrays yield a 30% net electrolysis gain. Through electrolyte engineering, it is shown that beyond expected hydrophobic reactant enrichment, the chemistry of the insulator influences reaction rates via electroosmotic flow and Marangoni‐driven convection at the insulator‐electrode‐electrolyte boundary. This work establishes engineered fouling as a powerful strategy for enhancing electrochemical processes and provides a framework for designing advanced electrode architectures for ECL and electrosynthetic applications.

## Graphical abstract

Strategic electrode fouling enhances mass transport, primarily through electrochemically actuated lateral density gradients. Engineered fouling induces flow velocities up to 0.4 cm s⁻¹ in otherwise quiescent systems. Sub‐millimeter plastic features boost local rates by up to 290%, while micrometer‐scale arrays yield a 30% net electrolysis gain.

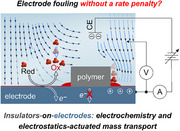

## 1 Introduction

The first half of the nineteenth century, marked by seminal works on electricity by, among others, Volta, Ampère, and Faraday,^[^ ^1^, ^2^ ^]^ laid the foundation of modern electrochemistry. Electrochemistry is now a truly multidisciplinary field^[^ ^3^, ^4^ ^]^ impacting the synthesis of commodity chemicals,^[^ ^5^, ^6^ ^]^ electrorefining,^[^ ^7^ ^]^ and separation science.^[^ ^8^, ^9^ ^]^ It drives advancements in batteries and fuel cells,^[^ ^10^, ^11^ ^]^ underpins corrosion science,^[^ ^12^, ^13^ ^]^ and supports the design and operation of chemical sensors.^[^ ^14^, ^15^ ^]^ Electrochemistry also fuels innovation in data storage and semiconductor technologies,^[^ ^16^, ^17^, ^18^ ^]^ advanced microscopy,^[^ ^19^, ^20^, ^21^ ^]^ and molecular electronics.^[^ ^22^, ^23^, ^24^ ^]^

The dependence of electrochemical rates on electrode area was already implicitly present in Faraday's work,^[^ ^2^ ^]^ and it is therefore unsurprising that, regardless of the specific electrode reaction, partially blocked electrodes (whether fouled or recessed), and electrodes exhibiting kinetic heterogeneity, are generally considered suboptimal conditions for electrolysis.^[^ ^25^, ^26^ ^]^ Reduced intimate contact between the electrode and electrolyte decreases current (i.e., the reaction rate),^[^ ^27^ ^]^ while surface heterogeneities introduce mass transport and kinetic complexity,^[^ ^28^, ^29^, ^30^ ^]^ ultimately limiting the accuracy and analytical potential of the redox measurement. To ensure intimate contact between the liquid and the electrified solid, electrochemists have therefore developed extensive knowledge of electrode cleaning procedures,^[^ ^31^, ^32^ ^]^ including strategies to facilitate the removal of nonconductive reaction products adhering to the electrode surface.^[^ ^33^, ^34^ ^]^ There are, however, certain situations where partial electrode blocking is not entirely detrimental. For instance, a small insulating block – relative to the solute diffusion length – can partially compensate for the loss of electroactive area.^[^ ^35^ ^]^ Radial diffusion of reactants from the solution above a micrometer‐sized insulating disk can restore the net current of a fouled electrode to a level comparable to that of an unblocked electrode.^[^ ^36^ ^]^ Experimental evidence also shows that local redox currents are enhanced at the junction where the electrode, electrolyte, and insulator converge.^[^ ^37^, ^38^, ^39^, ^40^ ^]^ In the latter case, the loss of electroactive area is offset by the strong electric field at the water‐insulator interface,^[^ ^37^, ^41^, ^42^ ^]^ and by enrichment of low‐solubility reactants at the electrode‐electrolyte‐insulator boundary.^[^ ^38^ ^]^ Surface hydrophobic effects, including changes to solvation shells,^[^ ^43^ ^]^ have been, for example, used to concentrate reactants near the electrode to enhance electrochemiluminescence,^[^ ^44^ ^]^ as well as to direct reaction rates and selectivity in electrosynthesis.^[^ ^45^, ^46^ ^]^

The height and width of the blocking object, as well as the net surface charge that develops on an insulator immersed in an electrolyte, are variables that can be easily tuned. It therefore follows that two insulator‐related physical effects, elaborated in more detail below, may offset the loss of wet electrode area: electro‐osmotic (EOF) flows^[^ ^47^ ^]^ and electrochemically driven natural convection.^[^ ^48^ ^]^ In this article, we seek to verify whether these two forms of mass transport can be engineered to have a fouled electrode outperform a clean electrode.

**Figure 1:**
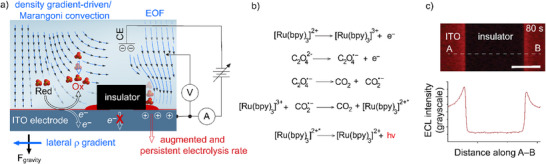
Electrochemiluminescence (ECL) of nonsurface‐active species on electrodes strategically fouled through lithographic techniques. a) Depiction of possible convective forces behind the observed near‐insulator current augmentation, including electrochemical (density gradient‐driven convection and Marangoni flow) and coulombic (electroosmotic flow, EOF) mechanisms. b) Graphical representation of the oxidative‐reduction mechanism for the ECL of [Ru(bpy)_3_]^2+^ with oxalate co‐reactant. c) ECL micrograph (2×, inverted microscope) and intensity plot profile (lower panel) acquired at an ITO‐coated glass electrode in aqueous H_2_SO_4_ (0.1 m, corrected to pH 6 with 10 m NaOH) in the presence of [Ru(bpy)_3_]Cl_2_ (4 mm) and oxalic acid (24 mm), 80 s after the potential was stepped from open circuit to +1.4 V (vs Ag|AgCl, 3.4 m KCl). The scale bar is 200 µm. The ECL emission (sampled along the A–B line) peaks near the edges of the insulator.

First, interactions between double‐layer ions and electric fields tangent to an insulating patch partially blocking an electrode share similarities with those near, for instance, the insulating wall of an electroosmotic flow (EOF) device.^[^ ^49^ ^]^ The electric field between the counter and working electrodes is likely to induce tangential liquid movement along the insulator's wall – an EOF convective force that may enhance electrolysis rates (**Figure** **1**a). Second, it is established that even in a nominally stagnant solution, some degree of convective stirring exists due to the action of gravity on electrochemically generated density gradients.^[^ ^50^ ^]^ This form of natural convection can distort amperometric currents from pure Cottrellian (diffusive) behavior,^[^ ^51^ ^]^ but efforts have generally focused on eliminating rather than utilizing this form of mass transport.^[^ ^52^ ^]^ Electrochemically driven buoyancy forces, which are measurable regardless of the electrode size,^[^ ^53^, ^54^ ^]^ have seldom been deliberately utilized to maximize reaction rates at macroscopic electrodes,^[^ ^55^ ^]^ but could be used for mixing solutions instead of pumps or thermal gradients.^[^ ^56^ ^]^ Electrolysis‐driven movement of charge‐balancing electrolyte ions creates natural convection due to molar volume differences between bulk solution and diffusive layer.^[^ ^50^ ^]^ We hypothesized that an electrode design with alternating blocked and active regions could induce convection, as charge transfer would be concentrated in the exposed areas while obviously absent in the blocked regions (Figure 1a).

Unlike convection driven by thermal gradients, electrochemically induced natural convection is significantly more complex to simulate,^[^ ^57^ ^]^ a challenge that is further complicated if, as mentioned above, an EOF also exists near the wall of the dielectric object (or array of objects) blocking the electrified surface. This article aims to determine, through experiments, to what extent “strategic” electrode fouling can augment electrolysis rates of small organic molecules in aqueous electrolytes, and to quantify the relative contribution of natural convection and EOF toward the rate enhancement (Figure 1a). The focus is on aqueous electrochemistry since water, driven by stricter regulations and rising environmental awareness, is becoming the chemical industry's preferred solvent.^[^ ^5^ ^]^ The aim is to augment rates using a minimal quantity of strategically placed insulators – photolithographic patterns – on standard electrodes. We use the term strategic fouling to emphasize its deliberate and beneficial nature, in contrast to detrimental or biofouling processes.^[^ ^58^, ^59^ ^]^ The key fouling parameters explored include the two‐ and three‐dimensional geometry of the insulating pattern, the hydrophobic or hydrophilic character of the dielectric material, the charge of the insulator‐electrolyte interface, the orientation of the electrochemically driven density gradient relative to the gravitational field, and properties of the electrolyte – specifically in regard to ionic mobility and ionic effects on water structuring (Hofmeister series).

## 2 Results and Discussion

### 2.1 Electrode Material Selection and ECL System Optimization for Minimal Surface Activity

Among the various redox imaging techniques, electrochemiluminescence (ECL) microscopy is ideally suited for this study,^[^ ^21^, ^60^ ^]^ as it provides the spatiotemporal resolution^[^ ^61^ ^]^ necessary to map redox rates around microscopic insulating patterns. ECL microscopy has been previously used to map fluid movement in electrochemical systems,^[^ ^62^ ^]^ and it does not carry the risk of adding convective disturbances,^[^ ^63^ ^]^ such as through a scanning probe measurement.^[^ ^64^ ^]^ Although ECL reaction mechanisms are far from being simple,^[^ ^65^, ^66^, ^67^ ^]^ light emission typically scales with the electrolytic current (Figure S1, Supporting Information); hence, ECL microscopy becomes a simple, direct, and *in‐situ* read‐out (light) on local redox currents across an electrified conductor.^[^ ^68^ ^]^ The ECL light emitter is populated through an electrode reaction, most commonly the simultaneous anodization of the chloride salt of tris(2,2′‐bipyridine)ruthenium(II) (hereafter [Ru(bpy)_3_]^2+^) and a co‐reactant, such as tripropylamine (TPrA).^[^ ^67^ ^]^ TPrA is however surface active and accumulates on the surface of several hydrophobic materials immersed in contact with its aqueous solutions.^[^ ^38^ ^]^ In order to minimize reactant enrichment as a cause of augmented redox rates at the electrode‐hydrophobe‐electrolyte interface we opted for a nonsurface‐active ECL co‐reactant – oxalate (C_2_O_4_^2−^). The mainstream oxidative‐reduction ECL path for the [Ru(bpy)_3_]^2+^ – oxalate system is shown in Figure 1b,^[^ ^69^ ^]^ and a representative ECL map (map of a surface immersed in an electrolyte), featuring both clean indium tin oxide (ITO hereafter) regions as well as regions masked by an insulating fouling object, is in Figure 1c. To map light emission, we utilized an inverted microscope and transparent electrodes partially fouled through photolithography (Figure S2, Supporting Information). Imaging the electrode‐electrolyte interface from the air‐electrode interface (i.e., not through the electrolyte) simplifies the experimental design but effectively restricts the choice of the electrode material to glass slides coated with ITO. Since there are only a few studies on the ECL of the [Ru(bpy)_3_]^2+^ – oxalate system on ITO, we initially performed spectroelectrochemical measurements to compare ECL responses between ITO and more conventional electrode materials, such as platinum and glassy carbon (GC). This step is important since the nature of the electrode material can strongly affect ECL reactions.^[^ ^70^ ^]^ As shown in Figure S3 (Supporting Information), ECL intensities are comparable on ITO, platinum, and GC. Further, the anodic bias at which the ECL peaks is also roughly independent of the nature of the working electrode (Figures S4–S7, Supporting Information), and therefore, unless specified otherwise, all the data presented hereafter refers to potentiostatic experiments carried out with a working electrode bias of +1.4 V versus Ag|AgCl.

**Figure 2:**
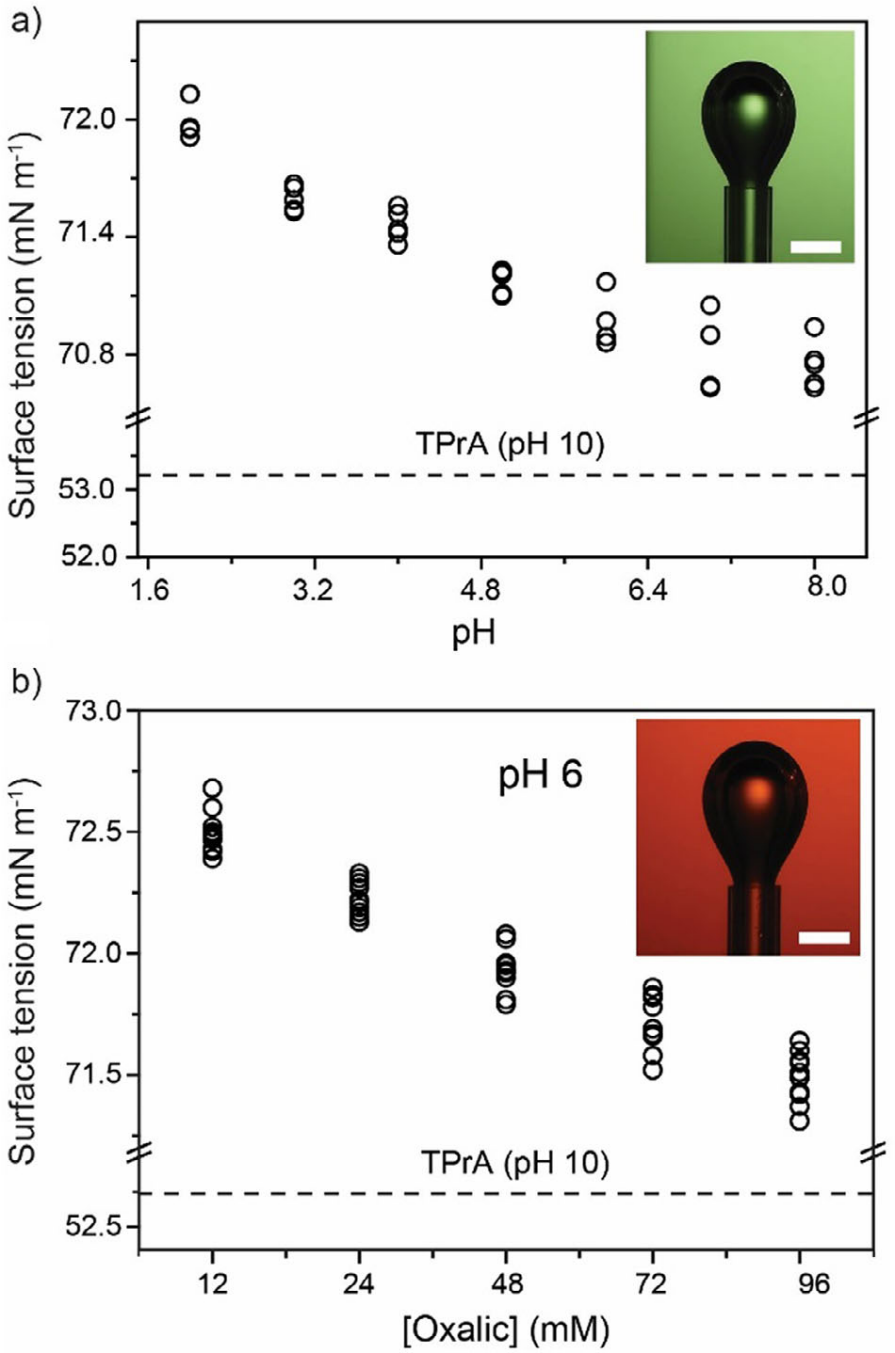
Surface activity of the ECL reactants inferred from bubble tensiometry measurements (Figure S8, Supporting Information). a) Surface tension of nitrogen gas bubbles (representative optical image as inset, scale bar 1.6 mm) in solutions of oxalic acid (1 mm) and aqueous sodium sulfate (0.1 m) corrected to the pH value specified in the abscissa axis. b) Changes to the water surface tension as the co–reactant (oxalic acid) concentration is varied between 12 and 96 mm (with 4 mm [Ru(bpy)_3_]^2+^ in 0.1 m aqueous sodium sulfate, pH 6). The inset shows a representative optical image of a nitrogen bubble in 4 mm [Ru(bpy)^3^]^2+^ and 24 mm oxalic acid. The scale is bar 1.6 mm. Tabled surface tension data are in Table S1 (Supporting Information).

As mentioned above, the near‐hydrophobe ECL augmentation observed in Figure 1c, and further elaborated in this article, is unlikely to result from a localized reactant enrichment. To verify this, we measured the surface activity of aqueous oxalate solutions using nitrogen gas bubbles as a hydrophobic model to mimic the photoresist fouling material explored in this study. As shown in **Figure** **2**a, the surface tension of the nitrogen bubble‐oxalate solution interface is only marginally smaller than that of pure water (72.53 mN m^−1^ at 22.5 °C^[^ ^71^ ^]^), and changed only slightly with pH, ranging approximately from 71 to 72 mN m^−1^ (calculated as described in Figure S8 and Table S1, Supporting Information). These surface tension values are in agreement with previous literature,^[^ ^72^ ^]^ suggesting negligible surface accumulation of the co‐reactant. The horizontal dashed line marked in Figure 2 (≈53 mN m^−1^) represents the surface tension of a system (TPrA as co‐reactant) where the near‐hydrophobe ECL augmentation is, on the other hand, due to reactant enrichment (Figure S9, Supporting Information).^[^ ^38^ ^]^

To further rule out adsorption for our ECL system, X‐ray photoelectron spectroscopy (XPS) experiments were conducted on films of both hydrophilic and hydrophobic photoresists (NLOF‐2035 and SU‐8 2002) incubated in the ECL solution. The survey and high‐resolution spectra of the photoresist pre‐ and post‐incubation did not indicate the presence of adsorbed reactant on the surfaces (Figures S10–S13, Supporting Information). As a further note, since the pH of the electrolytic solution affects the brightness of the [Ru(bpy)_3_]^2+^ – oxalate system (Figure S14, Supporting Information),^[^ ^69^ ^]^ surface tension data of Figure 2a also removes concerns about a possible surface enrichment‐pH relationship.

Notably, the two pK_a_ values of oxalic acid are 1.2 and 4.2, and therefore, based on solubility alone, we would expect surface tension to increase with pH, opposite to what is found experimentally (Figure 2a). We do not have a definitive explanation, but it is possible that the minor surface tension drop observed with increasing pH may involve an increase in the charge of the aerial surface of water under large hydroxide concentrations, which may reduce the cohesive forces between water molecules.^[^ ^73^ ^]^ Figure 2b explored the possibility of changes to the water surface tension in response to changes to the concentration of the ECL reactants, which is important to ascertain because the electrochemically driven convection introduced in the previous section, and plausibly behind the near‐hydrophobe augmentation shown in Figure 1c, may be due in part to surface tension gradients originating from a Marangoni effect.^[^ ^34^ ^]^ There are negligible changes to surface tension with changes to the oxalate concentration, indicating that a Marangoni effect, if present, would have to originate from local changes in electrolyte concentration rather than changes to the co‐reactant concentration (vide infra).

### 2.2 ECL Under Controlled Fluid Dynamics

Despite precautions to select an ECL system that minimizes adsorption effects,^[^ ^38^, ^45^ ^]^ the ECL emission is still evidently augmented near the electrode‐fouling object (Figure 1c), suggesting the onset of some form of electrochemically driven convection augmenting the electrolysis rate. However, while electrolytic currents generally scale with convection, the same is not necessarily true for the rate of a homogeneous reaction that requires a fruitful encounter between two electro‐generated molecules.^[^ ^74^ ^]^ It is therefore possible that increased stirring could cause a drop – not an increase – in ECL. Hydrodynamic rotating disc electrode (RDE) experiments coupled to light intensity measurements confirmed that for rotation speeds between 100 and 2000 rpm, both current and ECL for the [Ru(bpy)₃]^2^⁺ – oxalate system scaled with fluid velocity. Such rotation speeds are low enough to maintain the hydrodynamic system within the laminar flow regime (Reynolds number <2000), and data in Figure S15 (Supporting Information) show that stirring the system, moving for example from a nominally quiescent system (ECL measured 40 s after the potential step) to a radial fluid velocity of 0.14 cm s^−1^ (100 rpm),^[^ ^75^ ^]^ caused a ≈2.8‐fold enhancement in ECL intensity. A radial fluid velocity of 1.57 cm s^−1^ (500 rpm) led to a 6‐fold ECL enhancement and the increase in ECL approached a plateau ≈1000 rpm (radial fluid velocity of 4.44 cm s^−1^) where the light emission is ≈8 times that recorded for the unstirred (and un‐fouled) system.

### 2.3 Quantification of the Near‐Insulator ECL Augmentation

**Figure 3:**
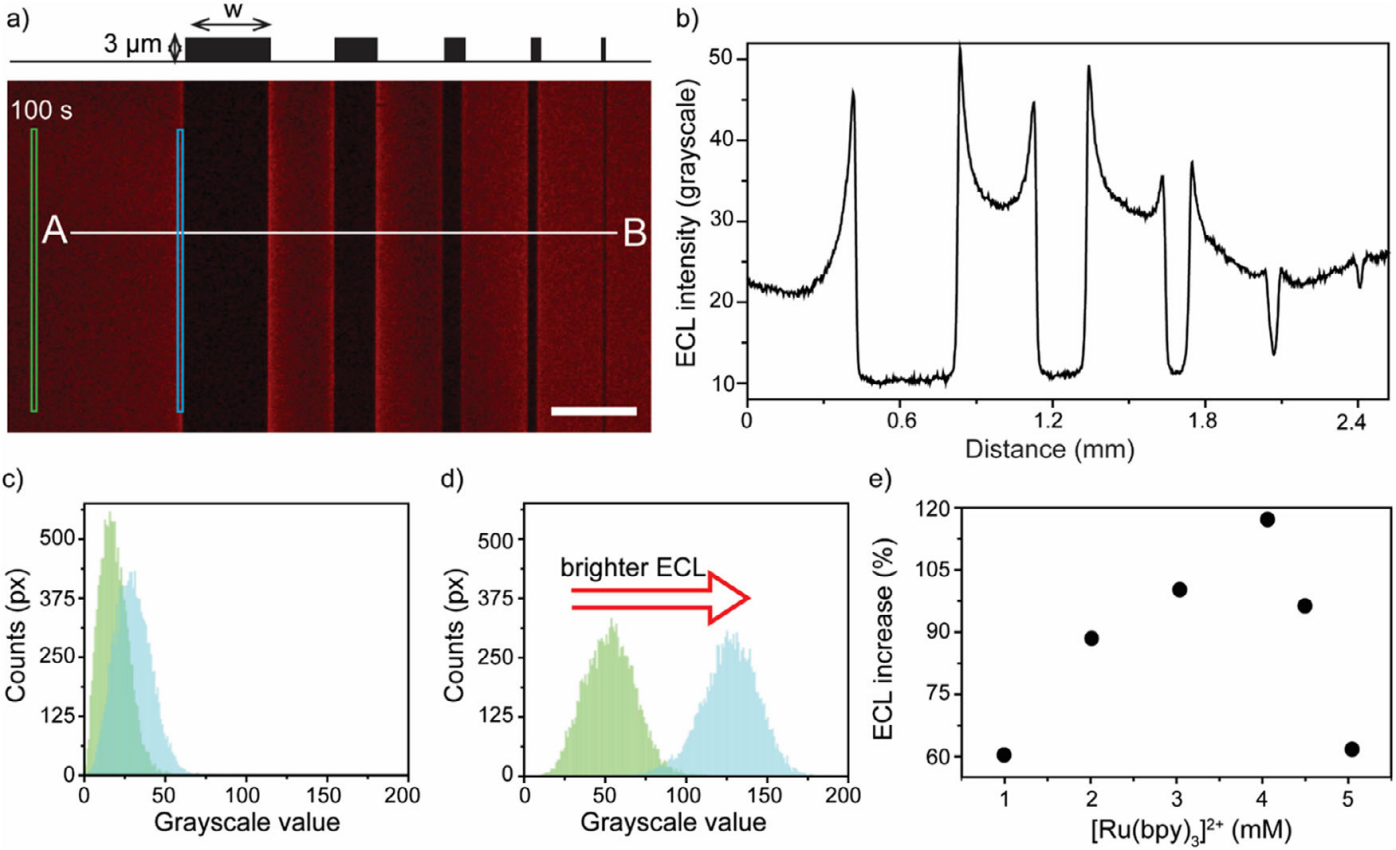
Near‐insulator augmentation of electrolysis rates revealed through ECL microscopy. a) Representative ECL micrograph (2×, inverted microscope, 1100 × 1500 px) for the anodic electrolysis of [Ru(bpy)_3_]^2+^ (4 mm) and oxalate (24 mm) in aqueous H_2_SO_4_ (0.1 m, corrected to pH 6) sampled 100 s after the ITO working electrode potential is stepped from open circuit to +1.4 V vs Ag|AgCl, 3.4 m KCl). The scale bar is 400 µm. b) ECL intensity (grayscale value) profile sampled across the A – B line marked in (a). The lithographically deposited line‐shaped patterns consist of a phenolic photoresist resin with melamine cross–linker (NLOF‐2035), 3 µm thick and of width (W) as depicted in Figure (a) upper panel, ranging from 400 to 25 µm. c,d) ECL intensity histograms for the background (clean electrode region, green) and the near‐insulator region (blue), as marked in (a) by color‐coded rectangles (10 × 1200 px). The histograms are generated by integrating the counts across 256 grayscale intensity levels within the marked region of interest. The electrolysis times in (c) and (d) are 100 s each, and the concentrations of the [Ru(bpy)_3_]^2+^ are 1 and 4 mm respectively. A set of the histograms and their plot profile is provided in Figure S16 (Supporting Information). e) Plot showing the maximum percentage increase in ECL near the insulator (e.g., blue vs. green region in (a)) compared to the background, as a function of [Ru(bpy)_3_]^2+^ concentration, with a constant 1:6 molar ratio of ECL dye to co‐reactant. The data points in (e) are integrations of the ECL intensity histograms. The complete ECL histogram and micrographs series are in Figures S17–S22 (Supporting Information).

These hydrodynamic ECL experiments support the hypothesis that a convective factor contributes to the enhanced electrolysis rate observed near the photoresist edge (Figure 1c). The nature of this convection was then explored systematically, beginning with an analysis of the ECL microscopy data in **Figure** **3**. With our lithographically fouled electrodes, the ECL enhancement factor – ECL near the insulator relative to that recorded at the same electrolysis time over clean electrode regions away (at least 600 µm away) from the insulator – was up to 2.1 (Figure 3a,b) for NLOF 2035 (thickness of 3 µm) without any deliberate external stirring. This estimate is through a simple but very informative line scan analysis (e.g., A–B line marked in Figure 3a) of greyscale intensities. Sampling larger regions of interest, that is repeating this investigation (or analysis) for increased areas of the blue and green boxes marked in Figure 3a, indicates a comparable ECL enhancement factor (2.4‐fold in Figure 3d (concentration of 4 mm [Ru(bpy)_3_]^2+^, 24 mm oxalic; Figure S16, Supporting Information). A comparison between the ECL enhancement recorded at 100 rpm using a clean RDE and the 2.4‐fold enhancement of Figure 3d demonstrates that fouling alone, even in a nominally quiescent electrode, can induce flow velocities close to 0.14 cm s^−1^. Data in Figure 3b show also a dependence of hydrolysis rates (hence ECL) on the width (w) of the fouling object, a finding which supports the onset of convective mass transport due to an electrochemically driven density gradient: a larger region with negligible redox rates (larger photoresist lines) adjacent to a clean electrode region of fixed size has the effect of maximizing the contrast between volumes of solution with mismatched densities. In other words, larger blocks result in larger solution volumes of mismatched density, leading to greater lateral contrast in buoyant forces, hence greater solution agitation. The direction of the agitation caused by the electrolysis occurring over a laterally heterogeneous electrode is a point that will be discussed in more detail below.

Furthermore, in support of convection originating from the passage of an electrolytic current is the observation of the insulator‐induced ECL augmentation scaling with the concentration of luminophore and co‐reactant (Figure 3b–e). Increasing the concentration of redox‐active species leads to larger electrolysis rates (current), hence maximizing the difference between the density of the solution above the insulator (bulk density) and that within the depletion layer (i.e., in the region of the solution above the accessible ITO).^[^ ^53^ ^]^ As shown in Figure 3e, holding the molar ratio between [Ru(bpy)_3_]^2+^ and oxalate constant at 1:6 (and the pH to 6), the percentage of ECL increase near the photoresist feature (Figure 3e; Figures S17–S22, Supporting Information) scaled with the reactants concentration up to ≈4 mm of [Ru(bpy)_3_]^2+^. The maximum percentage increase was nearly 120% and a further increase beyond 4 mm in the luminophore percentage increase was nearly 120% and a further increase beyond 4 4 mm in the luminophore concentration caused a decrease in the near‐insulator ECL augmentation effect. The reason is unclear, but it is possible that dark paths become significant at excessively large concentrations of luminophore and co‐reactant.^[^ ^76^, ^77^ ^]^ For this reason, in the remainder of the article, the concentrations of [Ru(bpy)_3_]^2+^ and oxalate are fixed at 4 and 24 mm, respectively.

We note that the drop in ECL augmentation observed when the [Ru(bpy)₃]^2^⁺ concentration exceeds 4 mm is unlikely to be due to increased solution conductivity, which could, for instance, suppress the EOF contribution.^[^ ^78^ ^]^ For example, the solution conductivity at the lowest reactant concentrations in Figure 3e ([Ru(bpy)₃]^2^⁺, 1 mm; oxalate, 6 mm) is 18.5 mS cm^−1^, which is comparable to the 20.9 mS cm^−1^ measured the highest concentrations tested ([Ru(bpy)₃]^2^⁺, 5 mm; oxalate, 30 mm). Since the supporting electrolyte concentration remains constant (0.1 m sulfate), changes in solution resistance across the data in Figure 3e are negligible. However, control experiments with an ECL solution consisting of 4 mm of [Ru(bpy)₃]^2^⁺, 24 mm oxalic acid in 0.2 m aqueous sulfate (32.9 mS cm^−1^) showed a decrease in near‐insulator ECL enhancement (Figure S23, Supporting Information). This effect is attributed to the suppression of EOF at very high ionic strength,^[^ ^78^ ^]^ as discussed further below.

**Figure 4:**
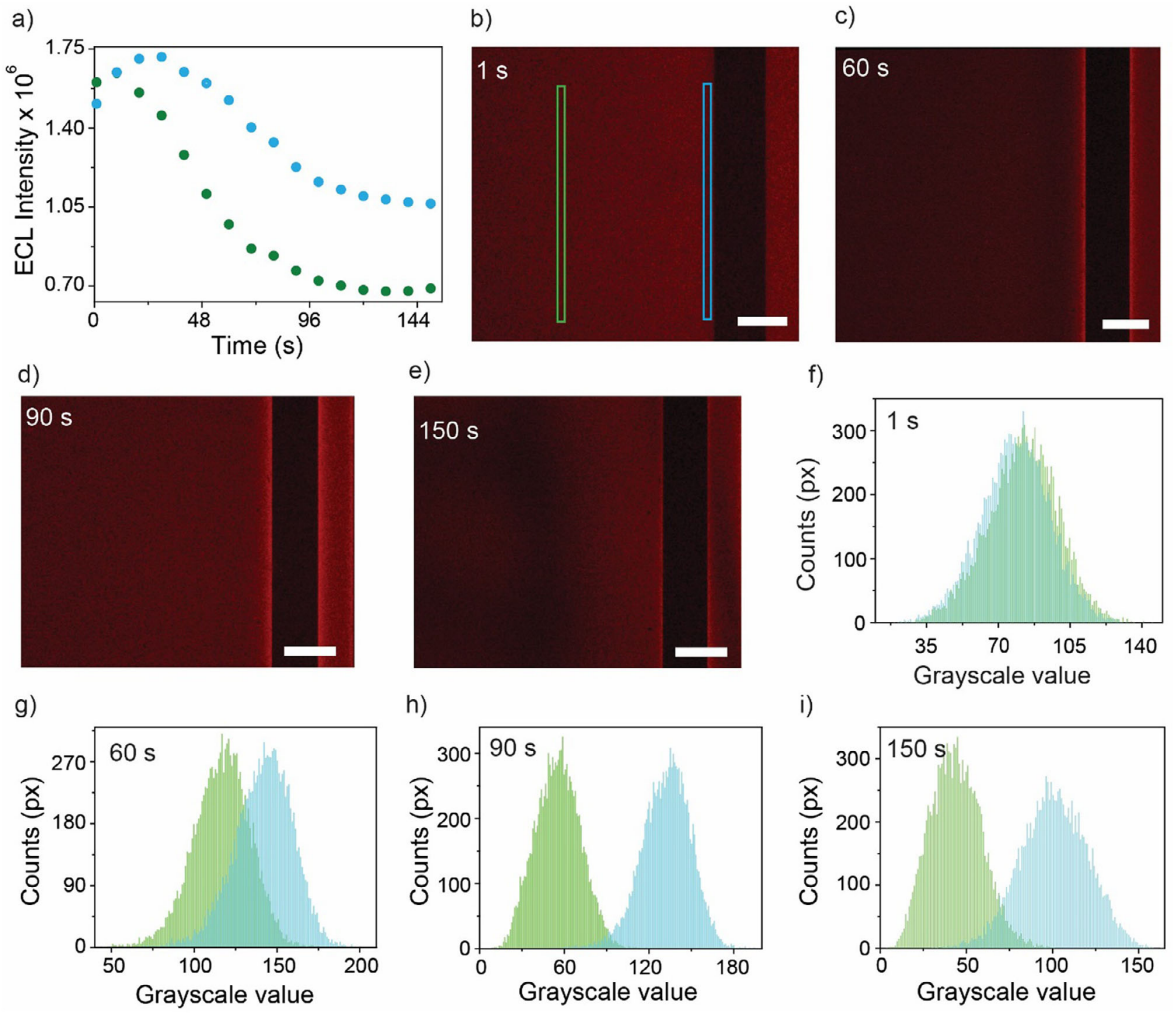
Non‐Cottrell decay of ECL emission around an electrically insulating object. a) Time evolution after the initial potential step of local ECL intensity sampled at clean and near‐insulator regions (green and blue rectangles with the sizes 10 × 1200 px in panel. The complete set of histograms and the non‐Cottrell decay emission for several lines are shown in Figures S24 and S25 (Supporting Information). b) The ITO‐glass electrode is partially fouled with photoresist patterns (NLOF 2035). The ECL reaction is the electrolysis (1.4 V vs Ag|AgCl, 3.4 m KCl) of an aqueous solution of Ru(bpy)_3_^2+^ (4 mm) and oxalic (24 mm) in aqueous 0.1 m H_2_SO_4_ (pH 6, adjusted by dropwise addition of 10 m NaOH). The ECL intensity values are the weighted summations of the total 256 (0–255) grayscale intensity histograms. b–e) Selected time‐stamped ECL micrographs (2× inverted microscope) with color‐coded rectangles indicating the regions of interest (ROI) used for the ECL intensity analysis in (a) and in (f–i). The scale bars are 400 µm. f–i) Time‐stamped histograms obtained from the analysis of ROI in micrographs (b–e). The histogram color matches the ROI coloring in (b), with the green histogram representing the intensity of the background and the blue histogram reflecting the intensity of the near‐insulator region. The time stamps indicate the time elapsed after the potential step. The complete set of ECL histograms, the corresponding micrographs and ECL video are provided in Figures S26, S27, and Video S1 (Supporting Information).

Another evidence in support of convective instability accounting for the near‐insulator augmentation discussed above would be a departure from the typical asymptotic decay predicted by the Cottrell equation (current scaling with the square root of time).^[^ ^57^ ^]^ Time‐stamped micrographs and histograms are shown in **Figure** **4**, and summarized spatiotemporal data (Figure 4a) compare vis‐a‐vis ECL emission near the insulator versus away from it, indicating convective instability for both the nonsteady as well as steady‐state part of the potentiostatic experiment. About 20 s after the voltage bias of the partially fouled ITO electrode is switched from an open circuit to +1.4 V, the rate of the light‐emitting electrode reaction peaks near the insulator (Figure 4a, blue symbols), implying that convection has reached such an amplitude to completely overbalance the drop in the diffusion driving force. The thickness of the diffusion layer away from the insulator is, at this time, still increasing, demonstrated by the ECL rate in the stagnant (clean) regions of the system continuing to drop (Figure 4a, green symbols). As the ECL rate over clean electrode regions approaches a steady state (green symbols, > 90 s, see also Figures S24, S25, and Video S1, Supporting Information) due to natural convection, the near‐insulator ECL reaches a maximum, which is evident in the lateral shift of the histograms in Figure 4f–i (see also Figures S26 and S27, Supporting Information). Past this maximum, the ECL enhancement effect reaches saturation and remains stable, or only slightly decreases, over time (Figure 4a).

As shown in Figure S28 (Supporting Information), prolonged ECL imaging experiments – comprising two consecutive 1‐h cycles of continuous anodic biasing – resulted in no visible signs of delamination, swelling, or chemical degradation of the photoresist (NLOF 2035).

### 2.4 Density Gradient‐Driven Convection Actuated by Electrochemistry on a Heterogeneous Surface

**Figure 5:**
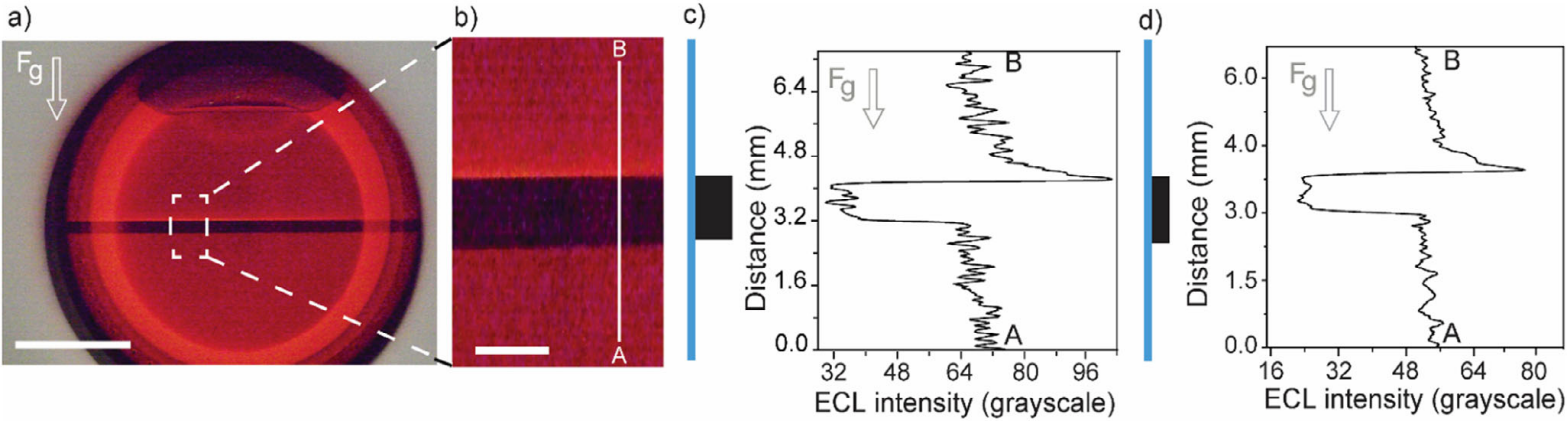
Evidence of electrochemically induced density gradient‐driven convection at a vertically mounted ITO electrode. a,b) ECL micrograph (scale bar is 1 cm) showing augmented electrolysis rates above a 1‐mm wide and 6 µm thick line‐shaped insulating pattern (NLOF 2035). The cell contained 4 mm [Ru(bpy)_3_]^2+^ and 24 mm oxalate in 0.1 m sodium sulfate (pH 6) (Video S2, Supporting Information). The direction of gravity (*F*_g_) is specified in the figure. The working electrode was biased at +1.4 V vs. Ag|AgCl (3.4 m KCl), and the ECL image shown in (a)–(b) was captured 80 s after electrolysis began. c,d) ECL intensity line profiles sampled across a rectangular region of interest (A–B line, 350 × 1200 px) for insulating patterns with thicknesses of either (c) 6 or (d) 3 µm. The electrolysis time was 80 s in (c) and 100 s in (e).

The difference in ECL intensity near and away from the photoresist generally reaches its maximum ≈70 s after applying the bias voltage (Table S2, Supporting Information). The presence of an ECL maximum at intermediate electrolysis times indicates that of mass transport not being only through diffusion, with the experiments discussed up to this point supporting convection originating from gravity acting on an electrochemically generated lateral density gradient. As definite proof, we performed an experiment with a vertical ECL cell (**Figure** **5**a) which allowed imaging of ECL patterns around insulators under a different relative alignment between the gravitational field (F_g_) and working electrode. Data in Figure 5 shows greater ECL augmentation on the upper portion of the insulator, which is highlighted qualitatively in (a) and (b), and quantitatively in (c) and (d). Since in a vertical cell, F_g_ runs parallel to the surface of the working electrode, the photoresist line acts as a physical barrier to falling liquid, blocking convective stirring underneath it. This explains the lower near‐insulator ECL augmentation observed below the insulating pattern compared to above it (Figure 5c,d). This specific electrolytic reaction increases solution density, and the gravitational pull on the denser near‐electrode volume generates significant convection when the moving liquid encounters an obstacle. Vertical ECL mapping with photoresist patterns of different width and height (Figure 5; Figure S29 and Video S2, Supporting Information) are in agreement with fluid agitation being significantly reduced under the insulator, directly demonstrating an interplay between buoyancy forces and electrochemically generated density gradients.

Figure S29 and Video S2 (Supporting Information) are in agreement with fluid agitation being significantly reduced under the insulator, directly demonstrating an interplay between buoyancy forces and electrochemically generated density gradients.

### 2.5 Coulombic Forces on Insulator‐Induced Convective Instability

**Figure 6:**
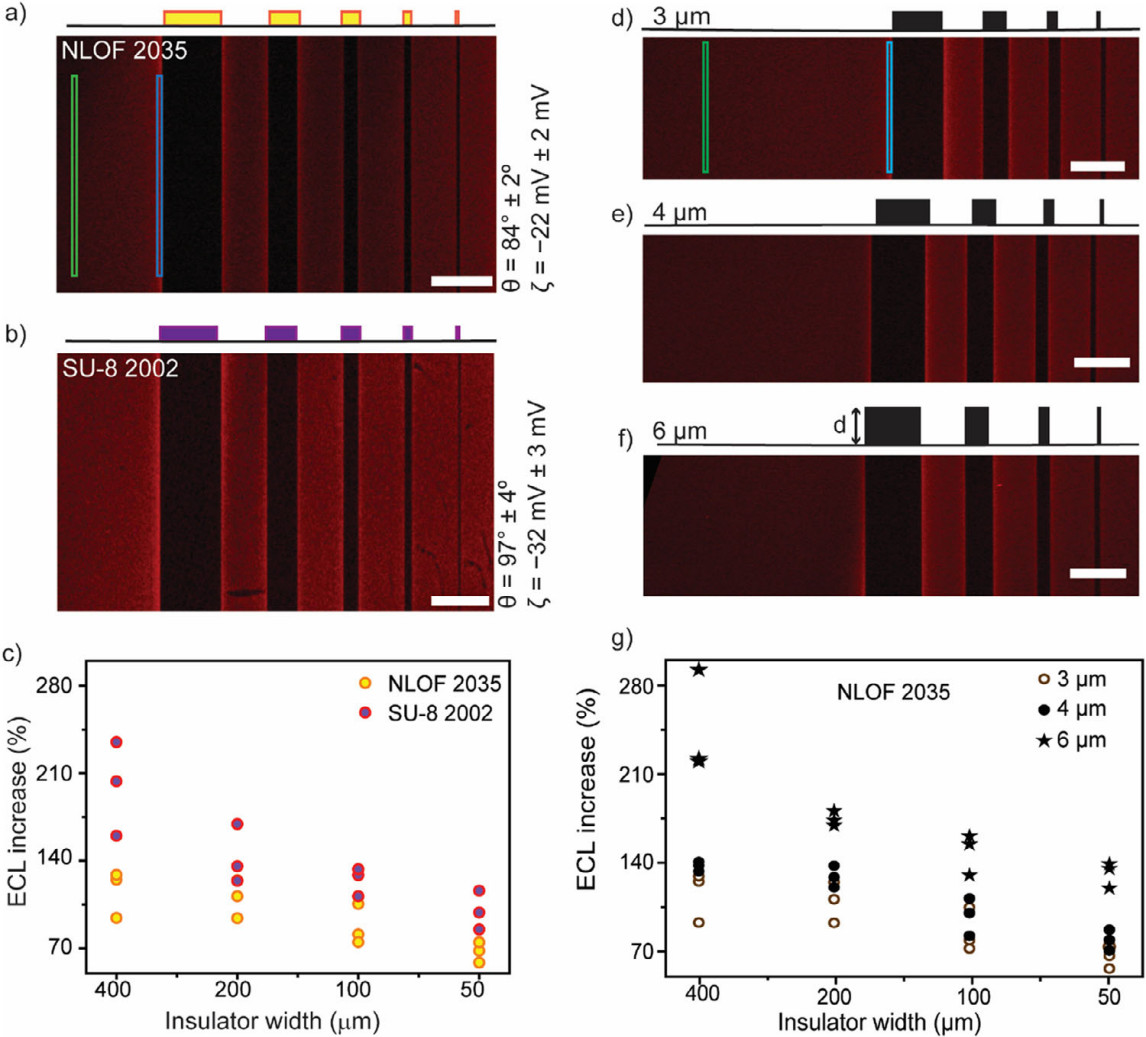
Augmentation of electrolysis rates as a function of the hydrophobicity, surface charge and height of the fouling material. a,b) ECL micrographs (2×, inverted microscope; scale bars: 400 µm) for ITO electrodes partially masked by 3 µm thick patterns of either hydrophilic NLOF 2035 (a) or hydrophobic SU–8 2002 (b). ζ potential values of the photoresist–electrolytic solution interface and water contact values of photoresist films are shown as labels to the ECL micrographs. Tabled ζ potential and contact angle values are in Tables S3 and S4 and Figures S30 and S31 (Supporting Information). d–f) ECL micrographs (2×, inverted microscope; scale bars: 400 µm) for ITO electrodes partially covered by NLOF 2035 line‐shaped features of variable thicknesses (3, 4, and 6 µm) and width (from 50 to 400 µm). The electrode bias is +1.4 V (vs Ag|AgCl, 3.4 m KCl), and the ECL solution is 4 mm [Ru(bpy)_3_]^2+^ and 24 mm oxalate in aqueous 0.1 m sodium sulfate (pH 6). ECL micrographs in (a,b,d–f) were obtained at electrolysis times between 60 and 100 s. c,g) Plots of the maximum percentage increase in near–insulator ECL intensity (relative to the clear electrode region, e.g., the green vs. blue regions of interest, each measuring 10 × 1200 px, marked in (a)). The entries in (c and g) are the weighted summations of the microscopy ECL grayscale intensity histograms. The complete set of ECL histograms is provided in Figures S32–S35 (Supporting Information).

We then turned to the question of whether or not EOF is operative at a partially fouled electrode. All the data presented up to this point indicate stirring triggered by a laterally heterogeneous electrolytic rate. Such knowledge can guide the design of the pattern, its shape, width, spacing, and electrode alignment relative to gravity, so as to maximize overall rates. The presence of this form of convection does not, however, rule out a mass transport contribution from EOF. Gaining insights into this type of field‐induced liquid flow can open new paths to further maximize redox rates. We reasoned that despite the extremely short height (micrometers) of the insulating wall (Figure 1a) systematic modifications to specific features of the photoresist could reveal the presence of an EOF. For instance, a higher surface charge density generally leads to stronger EOF because it enhances the electric field's effect on the counter‐ions in the electric double layer.^[^ ^79^ ^]^ A hydrophobic insulating surface^[^ ^80^ ^]^ and a greater absolute zeta potential value (ζ, positive or negative) of the insulator‐electrolyte interface tend to lead to stronger EOF,^[^ ^81^ ^]^ while the effect of changes to the length of the insulating wall (i.e., thickness of the insulator) are harder to predict. **Figure** **6** explores the role of the surface charge and hydrophobic/hydrophilic character of the insulator – it compares ECL augmentation at ITO electrodes partially fouled with either SU‐8 2002 or NLOF 2035 – and examines the effect of the wall length (insulator thicknesses).

The two photoresist materials have different hydrophobic/hydrophilic characters and different ζ potentials (Tables S3 and S4 and Figures S30 and S31, Supporting Information); hence, any difference in ECL augmentation near insulating patterns of identical geometric features would indicate the likely presence of an EOF and perhaps clarify on its magnitude. Data in Figure 6a–c point to a greater ECL augmentation for SU‐8 2002 (up to 240% ECL increase over the background, Figure S32, Supporting Information) compared to NLOF 2035 (up to 130% of ECL increase, Figure S33, Supporting Information). Further, laser Doppler velocimetry data, hydrophobicity, and ζ potential data suggest that the difference in ECL argumentation has a contribution from convection in response to EOF.

Moreover, if an EOF is present near the insulating features, this should perhaps scale with the area of the insulating wall, which can be changed by changes to the thickness of the photoresist.^[^ ^82^ ^]^ To investigate this, we deposited NLOF 2035 lines of different thicknesses. We note that although SU–8 2002 patterns showed the highest ECL augmentation (Figure 6c), for technical reasons, it is not possible to increase the thickness of SU–8 2002 features beyond 3 µm. As the thickness of the photoresist increases from 3 to 6 µm (Figure 6d–g; Figures S34 and S35, Supporting Information) the ECL intensity near the insulator was enhanced from 130% to 290% and this suggests fouling alone, even in a nominally quiescent electrode, can induce flow velocities close to 0.40 cm s^−1^ (200 rpm). Besides supporting the establishment of an EOF at partially fouled electrodes, the above experiment rules out a capacitive effect on the ECL augmentation: if capacitive pre‐concentration of ECL reactants on the polymer was involved, such an effect would be more marked in the thinner lines. This speculation originates from SU–8 2002 having a higher dielectric constant than NLOF 2035 (Table S5, Supporting Information).

### 2.6 Fluid Motion Driven by Electrochemically Actuated Surface Tension Gradients

The magnitude of EOF is generally directly proportional to the solution conductivity,^[^ ^83^ ^]^ which scales with ionic mobility. Therefore, an increase in ionic mobility should result in a corresponding increase in EOF magnitude. In this regard, our ECL mapping data suggest a third type of convective instability, electrochemically actuated, is present near the thin photoresist films. Data in Figure S36a (Supporting Information) show that changes to the electrolyte, specifically by using salts with anions of significantly different mobility (CH_3_COO^−^, F^−^, SO_4_^2−^, PO_4_^3−^; with a mobility of 4.3, 5.7, 8.3, and 9.6 (×10^−8^) m^2^ s^−1^ V^−1^ respectively)^[^ ^84^ ^]^ resulted in no clear correlation between the insulator‐induced ECL augmentation and mobility. However, ionic effects on the experimental ECL augmentation track relatively well the anion position in the Hofmeister series^[^ ^85^ ^]^ (Figure S36b, Supporting Information). If a concentration gradient of ions develops at a liquid interface, for instance in the vicinity of the electrode‐insulator‐electrolyte triple point, the resulting surface tension gradient can drive fluid motion through the Marangoni effect.^[^ ^34^ ^]^ ECL micrographs and the corresponding histograms in Figures S37–S40 (Supporting Information) point therefore to both EOF and Marangoni‐type convection being promoted by the partial fouling of the electrode, hence augmenting the local electrolysis rate.

### 2.7 Impact of Pattern Geometry on the Balance Between Rate Gain and Loss

**Figure 7:**
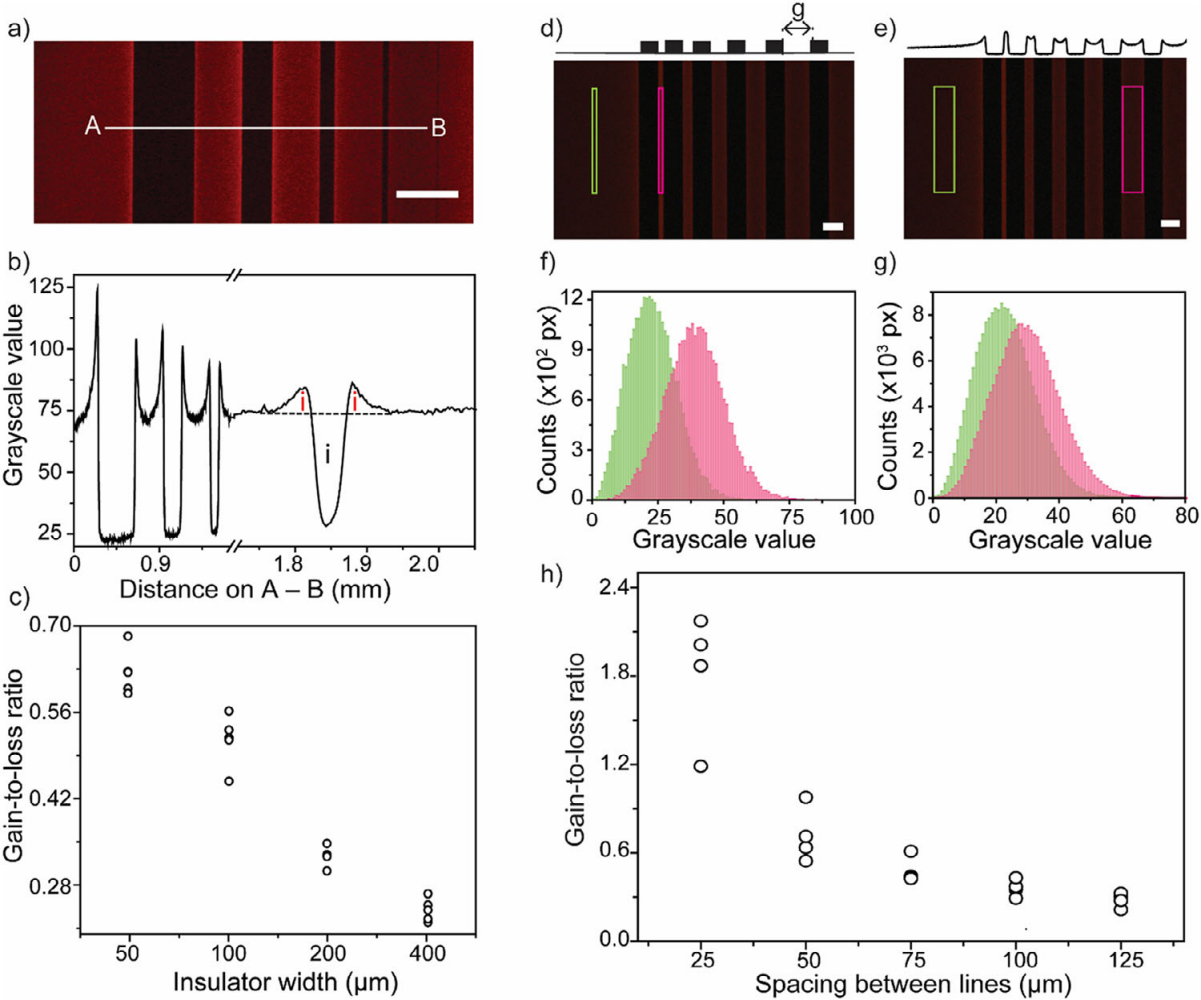
Strategic fouling: optimization of the width and the spacing between the insulating features. a) Representative ECL micrograph (2×, inverted microscope; scale bar is 400 µm). b) ECL intensity line plot profile sampled along the A–B line shown in (a). The *x*‐axis has a break at 1.65 mm to show a magnified view of values beyond this point. c) Plot of the maximum ECL gain/loss ratio as a function of the width of the insulating feature. The complete set of plot profiles and their micrograms and gain–to–loss ratio values are in Figure S41 and Table S6 (Supporting Information). The ECL gain‐to‐loss ratio was estimated from the ECL profile (b) by dividing the “gained” ECL counts (area marked by a red “i”) and the “lost” ECL (area under the insulator, marked as a black “i”). Individual data points in (c) are repeated experiments on independently prepared and analyzed samples. d,e) Representative ECL micrographs (2×, inverted microscope; scale bars are 50 µm). All micrographs sampled 100 s sampled 100 s after the ITO electrode potential was stepped from the open circuit to 1.4 V (vs. Ag|AgCl, 3.4 m KCl). The ECL solution contained 4 mm [Ru(bpy)₃]^2^⁺ and 24 mm oxalate in 0.1 m H_2_SO_4_ (pH adjusted to 6 with 10 m NaOH). The insulator is a NLOF 2035 film with a thickness of 3 µm. The width of the near‐insulator regions of interest marked in figure (pink rectangles) are 25 µm in (d) and 125 µm in (e). The green rectangles (25 µm wide in (d) and 125 µm wide in (e) define the regions used as “clean background”. f,g) ECL histograms of the background and near‐insulator regions for the small (25 µm) and large (125 µm) spacing between the NLOF 2035 insulating features. h) The plot of the maximum ECL gain‐to‐loss ratio as a function of the spacing between the insulators. The complete set of histograms, the corresponding micrographs, and the set of gain–to–loss ratio values are provided in Figures S42, S43, and Table S7 (Supporting Information).

The question now turned to whether or not the convective instability caused by the strategic electrode fouling is of such magnitude to offset the loss of the “wet” electrode‐electrolyte contact area. The question is not straightforward because the observed ECL enhancement depended on the size of the insulating block (Figures 3 and 6). For relatively spaced blocks, the loss in current due to the reduced wet electrode area of the 50 µm patterns (**Figure** **7**c; Figure S41 and Table S6, Supporting Information) is the best performer. The always outweighed the current gain near the insulator, regardless of the pattern width (Figure 7a–c), with gain‐to‐loss ratio for 50 µm wider patterns being 0.7 while it was ≈0.3 for 400 µm wider photoresists. We, therefore, concluded that the only way to increase the gain‐to‐loss ratio above one was to target a cumulative effect – bringing adjacent photoresist features closer together. As shown in Figure 7d–h, increasing the spacing between the insulating lines pushed the losses to exceed the gain (Table S7 and Figures S42 and S43, Supporting Information) and it was only for clean electrode gaps of 25 µm that the gain‐to‐loss ratio grew above unity (Figure 7h; Table S7, Supporting Information). Numerically, when the spacing between two patterns is 125 µm, the gain‐to‐loss ratio is ≈0.4, and when it decreases to 25 µm, the gain‐to‐loss ratio increases to nearly 2.0.

### 2.8 Electrode Fouling and Insulator Design for Large‐Area ECL Current Amplification

**Figure 8:**
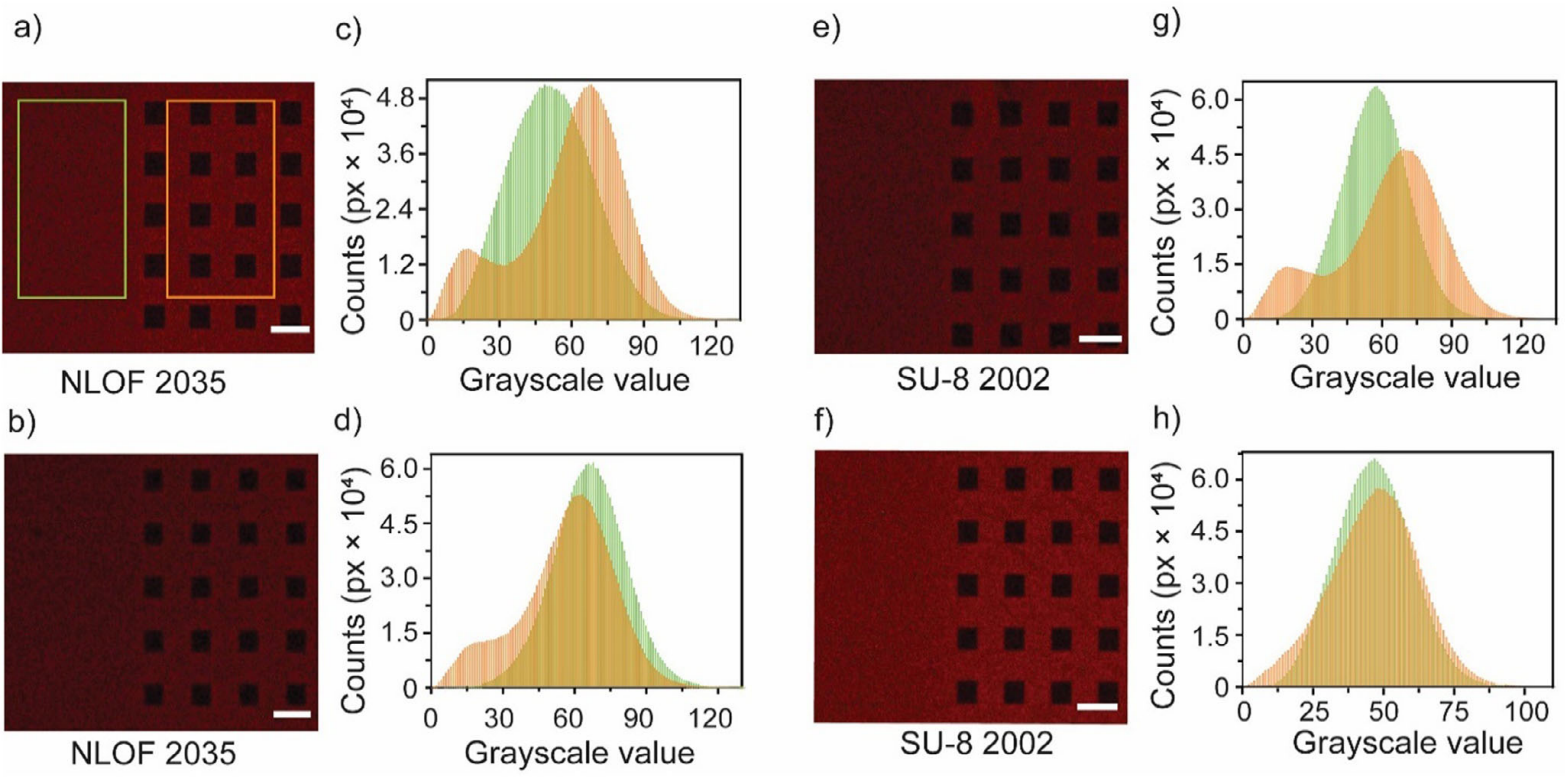
Strategic fouling: optimization of the insulating features with different spacing and sizes. ECL experiment of ITO‐glass (+1.4 V vs Ag|AgCl, 3.4 m KCl) partially fouled with the insulating photoresist for 4 mm [Ru(bpy)_3_]^2+^ and 24 mm oxalic in 0.1 m H_2_SO_4_ at pH 6 to investigate the intensity variation with the spacing between the insulators. a) Recorded ECL image (inverted microscope, 4×) of NLOF 2035 insulator for the pattern with 50 µm squares with 50 µm gaps of the ECL dye with oxalic acid; the green rectangle represents the area that accounts for the calculations in the background, and the orange rectangle represents the area selected in between the insulators. The sizes of the rectangles were kept constant, and it was 1204 px × 1879 px. b) Recorded ECL image (4× inverted microscope) of NLOF 2035 insulator for the pattern with 50 µm squares with 60 µm gaps of the ECL dye with oxalic acid. The scale is 100 µm. c,d) Corresponding histograms of the background and the gap between two insulators for the gap 50 and 60 µm, respectively. e) Recorded ECL image (inverted microscope, 4×) of SU‐8 2002 insulator for the pattern with 50 µm squares with 50 µm gaps of the ECL dye with oxalic acid. f) Recorded ECL image (inverted microscope, 4×) of SU‐8 2002 insulator for the pattern with 50 µm squares with 60 µm gaps of the ECL dye with oxalic acid. g,h) Histograms of the background and the gap between two insulators for the gap 50 and 65 µm for SU‐8 2002 photoresist, respectively. The complete set of histograms, corresponding micrographs, and gain‐to‐loss ratio values is provided in Figures S44–S47 and Tables S8 and S9 (Supporting Information).

With this knowledge of the optimal width and spacing of the insulator, the next logical step was to maximize the linear length of insulator‐electrode contact per unit area of the electrode. We opted for square‐shaped patterns to simplify geometric considerations on distances between neighboring blocks (Tables S8 and S9, Supporting Information). We proceeded to prepare and map a wide range of patterns with varying square widths and spacing guided by the results described above. The best‐performing patterns, along with their corresponding ECL histograms, are shown in **Figure** **8**a–h. It is difficult to determine the optimal material, spacing, and width through a visual comparison of ECL micrographs (Figure 8a,b,e,f; see also Figures S44 and S45, Supporting Information); however, clear clean ITO versus strategically fouled electrode trends emerge from the ECL histograms presented in Figure 8c,d,g,h and in Figures S46 and S47 (Supporting Information). For most patterns the gain‐to‐loss ratios exceeded unity (Table S9, Supporting Information) reaching 1.2, indicating that even on a macroscopic scale the gain, in terms of redox rates, from the insulator‐induced convective instability is offsetting the loss of intimate electrode‐electrolyte contact. The highest gain‐to‐loss ratio of 1.3 was achieved by SU–8 2002 and 1.2 for NLOF 2035 with square patterns featuring a width of 50 µm and a spacing of 65 µm. Although the ratios are nearly identical for both photoresist materials, NLOF thickness was 6 µm while the SU–8 2002 was 3 µm because of technical reasons and this reveals that the SU–8 2002 can give better performances than the NLOF 2035. Future efforts should be made with higher thickness of the photoresists with higher surface charge and hydrophobicity.

## 3 Conclusion

We demonstrate that electrodes fouled with minimal amounts of polymeric insulators can outperform clean electrodes. Sacrificing electrode‐electrolyte contact to microscale‐to‐millimetric dielectric blocks enhances net electrolysis rates, despite reducing the electroactive surface. This enhancement arises from electrochemically and electrostatically driven convective flows, visualized and quantified using electrochemiluminescence (ECL) microscopy. Our current report has focused mainly on the nonsurface‐active aqueous [Ru(bpy)_3_]^2+^/oxalate ECL system. However, our findings are general and apply to other systems, such as [Ru(bpy)₃]^2^⁺/TPrA in acetonitrile, as well as to alternative luminophores, such as luminol in aqueous alkaline electrolytes (Figure S48, Supporting Information).

By systematically varying the geometry, spacing, and surface chemistry of the polymer blocks, we have identified buoyancy‐driven convection as the primary driver of the observed rate increase, with local enhancements reaching 290% and flow velocities ≈0.40 cm s^−1^ without stirring. Gain‐to‐loss ratio – current enhancement near the insulator relative to the current lost to fouling – improves with smaller photoresist patterns and tighter spacing, reaching a net 1.28‐fold increase with 50 µm square blocks separated by 65 µm clean electrode gaps. The chemistry of the insulator plays a role, but not through expected hydrophobic effects that might locally enrich reactants. Instead, careful minimization of the reactants’ surface activity revealed that ion mobility and the insulator charge contribute significantly to the observed convective instability. This finding defines a scope for harnessing electroosmotic flows in strategically fouled systems. Hofmeister series trends further suggest a Marangoni effect at the electrode‐electrolyte‐insulator boundary, indicating opportunities for electrolyte engineering in multiphase electrochemical systems. Further efforts are needed to develop experimental and modeling tools that can quantitatively deconvolute the contributions of electroosmotic flow, density‐driven convection, and Marangoni effects.

The benefits of enhancing electrochemical reaction rates by introducing minimal amounts of insulating plastics onto electrode surfaces extend beyond ECL reactions, anodic processes, and are not restricted to transparent conductive substrates such as ITO electrodes. To demonstrate the broader applicability of strategic electrode fouling, we have conducted a proof‐of‐principle study on the cathodic electrosynthesis of valeric acid (VA) from levulinic acid (LA) – a reduction in which a keto acid is converted into a saturated carboxylic acid (Figure S49, Supporting Information). Electrosynthetic experiments were performed on glassy carbon (GC) electrodes. As shown in Figure S49 (Supporting Information), GC electrodes patterned with optimized microscopic insulating features (50 µm‐wide NLOF 2035 squares separated by 60 µm gaps of clean GC surface; see also Figure 8) led to a 1.6‐fold enhancement in VA electrosynthetic yield compared to unpatterned electrodes. This result highlights the potential to generalize the strategic fouling approach beyond ECL.

The current findings outline a scalable strategy for accelerating electrochemical processes by incorporating insulating structures to induce controlled stirring without moving parts or significant heat input – particularly valuable for heat‐sensitive biochemical applications. This approach could also be integrated with selective adsorption techniques (e.g., chiral oils, surfactant‐coated bubbles) to improve reaction selectivity without sacrificing efficiency.

## Conflict of Interest

The authors declare no conflict of interest.

## Supporting information



Supporting Information

Supplemental Video 1

Supplemental Video 2

## Data Availability

The data that support the findings of this study are available from the corresponding author upon reasonable request.
