# Supplementary material for: Stirring Without Stirrers: Polymer Fouling‐Driven Mass Transport Unlocks Order‐of‐Magnitude Gain in Electrochemiluminescence
Source: Adv Sci (Weinh). 2025 Jul 16;12(38):e06610. doi: 10.1002/advs.202506610 (PMC12520479; doi:10.1002/advs.202506610)
Supplement: Supplementary file 1 — Supporting Information [file ADVS-12-e06610-s001.pdf]

ADVANCED SCIENCE
Open Access

Supporting Information

for *Adv. Sci.*, DOI 10.1002/advs.202506610

Stirring Without Stirrers: Polymer Fouling-Driven Mass Transport Unlocks Order-of-Magnitude Gain in Electrochemiluminescence

*Wathsala Prasadini Kapuralage, Hemendra Kala, Mariusz Martyniuk, Nadim Darwish, Melanie MacGregor, K. Swaminathan Iyer and Simone Ciampi**

Supporting Information
©Wiley-VCH 2025
69451 Weinheim, Germany

Stirring without stirrers: Polymer Fouling-Driven Mass Transport Unlocks Order-of-Magnitude Gains in Electrochemiluminescence

Wathsala Prasadini Kapuralage,[a] Hemendra Kala,[b] Mariusz Martyniuk,[b,c] Nadim Darwish,[a] Melanie MacGregor,[d] K. Swaminathan Iyer,[e] and Simone Ciampi*[a]

[a] School of Molecular and Life Sciences, Curtin University, Bentley, Western Australia 6102, Australia,
Email: simone.ciampi@curtin.edu.au
[b] Department of Electrical, Electronic and Computer Engineering, The University of Western Australia, Crawley, Western Australia 6009, Australia
[c] Australian Research Council Centre for Transformative Meta-Optical Systems, The University of Western Australia, Crawley, Western Australia 6009, Australia
[d] Flinders Institute for Nanoscale Science and Technology, Flinders University, Bedford Park, South Australia 5042, Australia
[e] School of Molecular Sciences, The University of Western Australia, Crawley, Western Australia 6009, Australia

Table of Contents

Experimental Procedures 4
Supporting Figures 8
Figure S1. Relationship between applied current and ECL photon counts. 8
Figure S2. Photolithographic deposition of photoresists (NLOF 2035 and SU-8 2002) patterns on ITO-glass electrodes 9
Figure S3. Spectroelectrochemical characterization of the ECL on different electrodes. 10
Figure S4. Electrochemical characterization on ITO of the ECL solution. 11
Figure S5. Voltammetry studies on ITO glass for the combined and isolated constituents of the ECL solution. 12
Figure S6. Spectroelectrochemical characterization of the ECL solution on ITO glass at different potentials. 13
Figure S7. Electrochemical and spectroelectrochemical characterization of the ECL solution on ITO glass at low potential sweep rates. 13
Figure S8. Representative surface tension measurement using the rising bubble method. (a) The red color rectangle marks the region of interest. 14
Table S1. The surface tension of water, oxalic acid in different pH values. 15
Figure S9. Electrochemical characterization and ECL mapping in the presence of surface enrichment effects (surface-active ECL co-reactants). 18
Figure S10. X-ray photoelectron spectroscopy (XPS) analysis of NLOF 2035 films. 19
Figure S11. XPS analysis of NLOF 2035 photoresist films incubated with the ECL solution. 20
Figure S12. XPS analysis of SU-8 2002 films. 21

Figure S13. XPS analysis of SU-8 2002 photoresist films incubated with the ECL solution. 22
Figure S14. Spectroelectrochemical measurements towards ECL optimization through electrolyte pH changes. 23
Figure S15. Simultaneous hydrodynamic rotating disk electrode (RDE) and ECL intensity experiments.............. 23
Figure S16. Estimation of ECL enhancement factors (near insulators) through histogram analysis and cross-sectional line profiles. 24
Figure S17. Representative ECL micrographs and region-of-interest ECL intensity histograms............................ 25
Figure S18. Representative ECL micrographs and region of interest ECL intensity histograms. 26
Figure S19. Representative ECL micrographs and region of interest ECL intensity histograms. 27
Figure S20. Representative ECL micrographs and region of interest ECL intensity histograms. 28
Figure S21. Representative ECL micrographs and region of interest ECL intensity histograms. 29
Figure S22. Representative ECL micrographs and region of interest ECL intensity histograms. 30
Figure S23. Near-insulator ECl augmentation as a function of electrolytic support. 31
Figure S24. ECL intensity histograms indicating non-Cottrell decay of ECL rates (emission intensity) near blocked electrode regions. 32
Figure S25. Non-Cottrell decay of the ECL rate (emission intensity) near hydrophobe patterns. 33
Figure S26. Representative time-stamped ECL micrographs.. 34
Figure S27. ECL intensity histograms for electrode regions either near or away from the fouling object............ 35
Table S2. Electrolysis time required for the near–insulator ECL to reach a maximum intensity over the background. 36
Figure S28. Stability assessment of lithographically patterned ITO electrodes towards anodic biasing (+1.4 V vs Ag|AgCl, 3.4 M KCl).. 37
Figure S29. Buoyancy forces and density gradient-driven convection: effect of the insulating feature thickness and width. 38
Table S3. Zeta potential values for the photoresists NLOF 2035 and SU-8 2002. 39
Table S4. Contact Tabled water contact angle data for NLOF 2035 and SU–8 2002 films. 39
Figure S30. Surface zeta potential data for films of SU–8 2002. 40
Figure S31. Surface zeta potential data for films of NLOF 2035. 41
Figure S32. Representative ECL histograms for NLOF 2035 patterns of 3 µm thickness...................................... 42
Figure S33. Representative histograms for SU–8 2002 patterns of 3 µm thickness... 43
Figure S34. Representative ECL histograms for NLOF 2035 patterns of 4 µm thickness...................................... 44
Figure S35. Representative ECL histograms for NLOF 2035 patterns of 6 µm thickness...................................... 45
Table S5. Capacitance and dielectric constant values for the photoresists NLOF 2035 and SU-8 2002 at room temperature (~22 °C). 46
Figure S36. ECL increase percentage variation with different electrolytes. 47
Figure S37. Micrographs and histograms for triplicate ECL microscopy experiments in sulfate-based electrolytes. 48
Figure S38. Micrographs and histograms for triplicate ECL microscopy experiments in phosphate-based electrolytes.. 49

Figure S39. Micrographs and histograms for triplicate ECL microscopy experiments in fluoride-based electrolytes.......... 50

Figure S40. Micrographs and histograms for triplicate ECL microscopy experiments in acetate-based electrolytes.......... 51

Figure S41. ECL intensity augmentation as a function of the line-shaped insulating feature width.......... 52

Table S6. ECL gain-to-loss as a function of the photoresist line width.......... 53

Figure S42. Micrographs and histograms for 50 µm wide lines of NLOF 2035: varying the insulator–insulator distance.......... 53

Figure S43. Micrographs and histograms for 100 µm wide lines of NLOF 2035: varying the insulator–insulator distance.......... 54

Table S7. ECL gain-to-loss as a function of the spacing between adjacent photoresist lines.......... 55

Figure S44. ECL micrographs of ITO electrodes partially fouled by 50 µm wide insulating features separated by clean electrode gaps of different sizes.......... 55

Figure S45. ECL intensity histograms from the micrographs of ITO electrodes partially fouled by 50 µm wide insulating features separated by clean electrode gaps of different size.......... 56

Table S8. ECL gain-to-loss as a function of NLOF and SU–8 2002 photoresist geometry (size, spacing between adjacent photoresist pattern, and shape).......... 57

Figure S46. ECL micrographs of ITO electrodes partially fouled by 25 µm wide insulating features separated by clean electrode gaps of different sizes.......... 57

Figure S47. ECL intensity histograms at partially fouled ITO electrodes with 25 µm wide and different spacing. 58

Table S9. ECL gain-to-loss as a function of the size and the spacing between adjacent NLOF 2035 and SU–8 2002 photoresist squares.......... 59

Figure S48. Near-insulator rates augmentation for alternative ECL systems.......... 60

Figure S49. Electrochemical reduction of levulinic acid (LA) to valeric acid (VA): clean vs. fouled electrodes.......... 61

Figure S50. Four-point probe setup used to measure the resistivity of the ITO glass.......... 62

Figure S51. Custom-designed cells for spectroelectrochemical, microscopy, and cyclic voltammetry studies.......... 62

Figure S52. Spectroelectrochemistry setup.......... 63

Figure S53. Inverted microscope-based ECL setup.......... 64

Figure S54. Vertical cell to validate the density gradient-driven convection in the ECL system.......... 65

Figure S55. Profilometry analysis of the photoresist film thickness using a Dektak 150 surface profiler.......... 66

Figure S56. Photon counting setup. (a) Overview of the photon counting setup.......... 67

Figure S57. Experimental setup used for the measurement of surface tension.......... 68

Figure S58. Surface Zeta potential measurement setup using a Malvern Zetasizer.......... 69

Figure S59. Contact angle measurements setup.......... 69

Figure S60. Image of the cell used for capacitance measurements of the photoresists (dielectric constant estimation).......... 70

Figure S61. Rotating disc electrode (RDE)/ECL setup.......... 71

References.......... 71

Experimental Procedures

Materials. Unless otherwise stated, all reagents and solvents were of analytical grade and used without any further purification. Milli-Q™ water (18.2 MΩ cm resistivity at 25 °C) was used to prepare electrolytic solutions and for cleaning procedures. Tris(2,2′-bipyridyl)dichlororuthenium(II) hexahydrate ($[Ru(bpy)_3]^{2+}$, 99.95% trace metals basis), tripropylamine (TPrA, ≥98%), levulinic acid (LA, 98%), valeric acid (VA, ≥99%), acetonitrile (≥99.9%), 3-Aminophthalhydrazide, 5-Amino-2,3-dihydro-1,4-phthalazinedione (luminol, 97%), Tetra-*n*-butylammonium hexafluorophosphate ($TBAPF_6$, 98%), hydrogen peroxide (H2O2, 30 % w/w) were purchased from Sigma–Aldrich (Australia). Sodium dihydrogen orthophosphate (anhydrous, ≥99.0%) was purchased from ChemSupply (Australia). Sodium hydroxide (NaOH, 99.4%) was purchased from VWR Chemicals BDH (Belgium). Oxalic acid ($H_2C_2O_4$, 99.5%), and potassium chloride (KCl, 99.0%) were purchased from Ajax Finechem (Australia). Aqueous sulfuric acid (H_2SO_4, 95–97%) was obtained from Honeywell (Australia). The negative photoresist AZ® nLOF™ 2035 (phenolic resin with melamine cross-linker, abbreviated NLOF 2035) and its developer AZ 2026 MIF (metal-ion-free) were purchased from Microchemicals GmbH (Germany). The negative photoresist SU-8 2002 (epoxy-based negative photoresist) and its developer SU-8 developer (99.5-100% 1-methoxy-2-propanol acetate) were purchased from Kayaku Advanced Materials, Inc. (United States). Glass slides coated with indium oxide (In_2O_3) and tin oxide (SnO_2) (abbreviated as ITO, 50 × 50 × 1 mm, with a sheet resistance of 7–10 ohms per square) were purchased from Guangzhou Lepond Glass Co., Ltd. (China). The sheet resistance of the ITO film was measured using a four-point probe system (T2001A4, Ossila, UK, Supporting Information Figure S50) by taking measurements from at least 10 points on three different samples for each new batch of ITO slides.

Photolithography. The UV-assisted photolithographic deposition of negative photoresist patterns on the ITO slides followed the manufacturer's procedures of the photoresists, with only minor adjustments of the recommended temperature and baking time. Photolithography (Supporting Information, Figure S2) was performed in a cleanroom (Class 100) and the ITO slides were first cleaned by sequential rinsing with water, acetone, ethanol, and water, followed by drying under nitrogen gas flow (≥99.999%, Coregas Pty Ltd, Australia). Approximately 2 mL of photoresist solution was spin-coated (PWM32 controller spinner, Headway Research, Inc, United States) for 40 s (the rotation speed varied depending on the material and on the target, photoresist thicknesses) on the clean ITO surface. The thickness of the photoresist film was measured through optical profilometry (Dektak 150 surface profiler, Veeco, United States). The photoresist-coated ITO glass was then baked for 5 min at 110 °C and left to cool down at room temperature (22 ± 1 °C) for at least 5 min under ambient air. Photoresist films (NLOF 2035 and SU-8 2002) were exposed to patterned UV light (375 nm and 2.8 W cm^{-2}, maskless aligner; 365 nm, 7.3 mW cm^{-2} l for 2.5 s for photomask) either with a maskless aligner (MLA150, Heidelberg Instruments, Germany) or through a conventional pattern transfer from a photomask (MA6 mask aligner, SÜSS MicroTec, Germany). The patterned samples were then left to stand for at least 5 min under ambient air before being baked at 110 °C for 5 min. The samples were cooled down to room temperature and then developed by immersion (with gentle agitation) for 80 s in the supplier-recommended developer solution. The developing step removes photoresist material not exposed to the UV light patterns mentioned above. Developed samples were then immersed in water for 2 min, dried under a gentle flow of nitrogen gas, and baked for 5 min at 110 °C.

Electrolytic solutions for the electrochemiluminescence (ECL) measurements. For all electrochemical and ECL measurements, $[Ru(bpy)_3]^{2+}$ served as the electrochemiluminescent (ECL) dye. ECL solutions contained $[Ru(bpy)_3]^{2+}$ and oxalic in a 1:6 molar ratio (unless specified otherwise). The $[Ru(bpy)_3]^{2+}$ concentration ranged from 1 to 5 mM. The concentration of the sodium-based supporting salt was 0.1 M, with the anion being either sulfate (SO_4^{2-}), phosphate (PO_4^{3-}), fluoride (F^-), or acetate (CH_3COO^-). The electrolyte pH was adjusted to a specific value (between 4 and 8) by the dropwise addition of concentrated (10 M) aqueous NaOH. Electrolytic solutions for the control ECL experiment in the water contained $[Ru(bpy)_3]^{2+}$ (1 mM), TPrA as a co-reactant (10 mM), in a phosphate buffer solution (0.15 M) as the supporting electrolyte. ECL solutions were shielded from ambient light before use and used within a day of preparation.

Spectroelectrochemistry. Spectroelectrochemical measurements were conducted on unpatterned glassy carbon plates, on a platinum mesh, and on ITO electrodes to investigate changes in ECL emission as a function of electrode material, ECL emitter concentration, and electrolyte pH. The glassy carbon electrodes were 3 mm thick and had a diameter of 25 mm (Ted Pella, Inc., United States). The platinum mesh had a 7 × 6 mm overall size (EF-1355 SEC-C Gauze, 80 mesh, wire diameter of 80 µm) and was sourced from BASi (United States). Details on the ITO working electrodes are found in the Materials section. An Ag|AgCl "leakless" electrode (ET072-1, eDAQ, Australia), stored between experiments in aqueous 3.4 M KCl, was used as the potential reference electrode. A platinum wire (0.5 mm diameter) served as the counter electrode. The concentration of the ECL emitter, $[Ru(bpy)_3]^{2+}$, varied between 1 and 5 mM, and the electrolyte pH from 4 to 8. A quartz glass cuvette of 10 mm optical-path length (external dimensions 45 × 12.5 × 12.5 mm, QG10204-4, Aireka Cells, Hong Kong), fitted with a perforated PTFE cap/electrode holder, was used as a cell for the spectroelectrochemical experiments with the platinum mesh electrode. A small volume (1.75 mL) single-compartment electrochemical flow cell designed for microscopy experiments (Redoxme AB, Sweden) was used with the glassy carbon disk, while a custom PTFE

single-compartment cell (~12 mL capacity) was used for the measurements with ITO glass (Supporting Information, Figure S51). ITO was cleaned before the experiments as detailed above in the Photolithography section. The platinum mesh was cleaned through prolonged cyclic voltammetry experiments in 0.1 M H_2SO_4 (500 cycles, potential range set from 0 to 1.5 V, with a 0.25 V s^{-1} potential sweep rate). The glassy carbon electrode was mechanically cleaned by polishing it on micro cloth pads with alumina slurry in water, first for 10 minutes with 0.3 µm slurry and then for 10 min with 0.05 µm slurry. The polished electrodes were sonicated in water for 10 min, blown under nitrogen gas flow (≥99.999%, Coregas Pty Ltd, Australia), and stored in water before the experiments. Fixed bias (chronoamperometry) runs were performed with an EmStat3 potentiostat (PalmSens BV, Netherlands) holding a +1.4 V (vs reference) working electrode bias for 150 s. ECL emission spectra were recorded using a fiber-coupled diode array spectrometer (Flame Miniature Spectrometer, Ocean Optics, United States) (Supporting Information, Figure S52). Further details on the optical setup, the collection geometry, and the spectroelectrochemical cells are in the Supporting Information (Figures S51 and 52). The specific reactant and co-reactant concentrations, and electrolyte pH values are listed in the Supporting Information (Figure S14). All ECL solutions were left to equilibrate in the air for at least 5 min before the spectroelectrochemical measurement.

Electrochemiluminescence microscopy. All the ECL microscopy experiments were performed at room temperature (22 ± 1 °C). Time-lapse microscopy videos were recorded in a dark room using a Nikon Eclipse Ti2-U inverted microscope (Supporting Information, Figure S53). The microscope was equipped with a 5.9-megapixel CMOS color camera (DS-Fi3 Nikon, 2880 × 2048 pixels, 256 gray levels) and was controlled using the Nikon NIS imaging software. ECL generation across insulating photoresist-fouled ITO glass electrodes were mapped using either a 4×/0.13 NA (Plan Fluor, WD 17.2, Nikon) or a 2×/0.1 NA (Plan APO, WD 8.5, Nikon) objective. The camera gain was set to 64 dB, and the exposure time was set to 1 s. Frames of the time-lapse videos were recorded at a frequency of 1 Hz. The partially fouled ITO electrodes, produced through the photolithographic strategy described above, served as working electrodes and were rinsed with copious amounts of water before being blown dry under nitrogen gas flow before the ECL imaging procedure. A "leakless" electrode (ET072-1, eDAQ) was used as the reference electrode, and a coiled platinum wire (0.5 mm diameter, approximately 8 cm in length, 99.99+% purity, sourced from Goodfellow Cambridge Ltd., England) served as the counter electrode. The vertical distance between the counter and working electrodes was greater than 20 mm. Electrical connection to the ITO glass was achieved by pressing copper tape (3M 1245 conductive metallic tape, T124519, RS Components, Australia) on a portion of the ITO film not in contact with the electrolyte. The three-electrode system was controlled with a PalmSens4 potentiostat (PalmSens BV, Netherlands).

Unless it is specified otherwise, ECL microscopy was performed using a single-compartment PTFE cell (Supporting Information, Figure S51) filled with ~12 mL of ECL solution and the ECL solution contained 4 mM $[Ru(bpy)_3]^{2+}$ and 24 mM oxalate. The electrolyte pH was 6, and the anion of the sodium supporting salt was either SO_4^{2-}, PO_4^{3-}, F^- or CH_3COO^-. Once filled with electrolyte, the ECL cell was placed on the microscope stage, capped, and the cell's cap fitted with the reference and counter electrodes. The wet surface of the working electrode was placed in focus, and the system was left to stand undisturbed for at least 5 min before starting the microscopy experiment.

Experiments with vertically mounted ITO electrodes were conducted in a single-compartment PEEK cell with a capacity of ~12 mL total liquid capacity (Supporting Information, Figure S54). The ITO was partially fouled with a single line-shaped feature (1 mm wide, 40 mm long) of insulating photoresist (NLOF 2035). The photoresist thickness was varied between 3 and 6 µm and the thickness was measured using a Dektak 150 surface profiler (Veeco, United States) (Supporting Information, Figure S55). The cell was mounted on a XYZ stage, and the camera, a Nikon D850 DSLR camera (35.9 × 23.9 mm CMOS sensor), fitted with a 60 mm AF-S Micro-NIKKOR F/2.8 G ED lens, was then focused on the plane where the photoresist line met the ITO surface (Supporting Information, Figure S54). The three-electrode system was connected to a potentiostat (EmStat3) and left to equilibrate undisturbed for at least 5 min. The potential of the ITO working electrode was then stepped from the open circuit to +1.4 V (vs Ag|AgCl, 3.4 M KCl), and the ECL emission was imaged for approximately 150 s, setting the exposure under aperture priority, the detector sensitivity to ISO 25600, the JPEG quality as 3840 × 2160 "fine image" quality, and selecting a 25 p movie frame rate option.

Mapping and quantitation of the ECL augmentation. Micrographs (frames) of the time-lapse videos capturing the real-time progression of the ECL were analyzed using the open-source image processing package Fiji.[82] Unless specified otherwise the region of interest (ROI) was selected to include both "fouled" (areas with insulating photoresist patterns) and "clean" (unpatterned) electrode regions. The ROI, defined using the software's selection tool, was typically a rectangle measuring 10 × 1200 px (see for example Figure 2), with its long side aligned parallel with the longer dimension of the line-shaped photoresist feature. Before analyzing the ECL patterns (only the red channel was evaluated) the software was used to rotate ECL micrographs so to ensure a vertical alignment of the rectangular photoresist feature. For the analysis of each dataset (including profiles and histograms), the size and location of the ROI were held constant.

Photon counting experiments. The quantitative relationship between the total electrochemical current measured during the galvanostatic (current-controlled) experiment at unpatterned ITO electrodes and the ECL intensity was investigated using photon counting experiments. These experiments were conducted with an avalanche

photodiode (PerkinElmer SPCM-AQR-14 single photon counting module, based on a silicon APD operating in Geiger mode). The experimental setup is described in more detail in the Supporting Information (Figure S56). The photon counting experiments were conducted at 22 ± 1 °C, anodizing an ECL solution consisting of 4 mM $[Ru(bpy)_3]^{2+}$ and 24 mM oxalic acid in 0.1 M sulfate at pH 6.

Surface tension measurements. To demonstrate that the near-insulator electrolysis augmentation happens regardless of the reactant's surface activity we performed rising bubble tensiometry experiments to evaluate the surface tension of the ECL solution–nitrogen gas interface.[83, 84, 85] Oxalic acid (1 mM) solutions were prepared at pH values ranging from 2 to 8, by dropwise addition of aqueous NaOH (10 M). ECL solutions were prepared with a fixed concentration of $[Ru(bpy)_3]^{2+}$ (4 mM) varying the concentration of oxalic acid (12, 24, 48, 72, and 96 mM) and the pH adjusted to 7 using dropwise addition of aqueous NaOH (10 M). The base of a polystyrene cuvette (10 × 10 × 45 mm) was perforated and fitted with a borosilicate glass capillary (~1.6 mm outer diameter, Kimble, United States) to allow gas bubbles to rise through the liquid sample contained within the cuvette (see Supporting Information, Figure S57). A short section of silicone tubing served as an adapter between the capillary and the tip of a Luer slip-type threaded plunger, gas-tight syringe (Hamilton, 81341, United States). Before the surface tension measurement, the modified plastic cuvette was immersed for 10 min in an aqueous KOH solution (0.2 M) in order to clean the glass capillary. The cuvette was then rinsed with water and kept submerged before use. The shape and size of the nitrogen bubble entering via the glass capillary pure water samples, or samples of electrolytic solutions, were captured in time-lapse videos recorded with a Nikon D850 DSLR camera fitted with a 60 mm macro lens (Nikon, AF-S Micro Nikkor 60 mm f/2.8G) mounted on a multi-axis precision translation stage. The movie quality was set to 1920 × 1080, 30p × 4 ("slow-motion"), the image sensor to DX 24 × 16 mm, and the exposure set as shutter priority (the actual frame rate was 6.6 ms/frame). The temperature of the solution in the cuvette was recorded with a thermocouple before and after releasing the bubble. Bubbles used for the surface tension analysis were of such a volume that they remained stationary for approximately 10 s before convective and buoyant forces caused their detachment from the capillary. The setup was held inside a vibration-isolated metallic box. Relevant frames were selected using the image processing package Fiji,[82] and analyzed through the open-source OpenDrop software (Supporting Information, Figure S8).[86] The outer diameter of the glass capillary served as an internal reference for the surface tension analysis and was first measured with a Vernier caliper (ca. 1.67 mm) and then validated through surface tension measurements of pure water by assuming a surface tension of 72.98 mN m^{-1} at 22.5 °C.[87]

Zeta potential measurements. Zeta potential (ζ) measurements of the photoresist–ECL solution (i.e., the potential difference between the bulk electrolyte and the slipping plane) were performed at 22 ± 1 °C using laser Doppler velocimetry (LDV) in phase analysis light scattering (PALS) mode. The motion of charged tracer particles (transfer standard ZTS1240, Malvern Panalytical, United Kingdom) was tracked as the position of the laser of the dynamic light scattering (DLS) spectrometer (Malvern Zetasizer Nano ZS, Malvern Panalytical, United Kingdom) was incrementally displaced from the film surface into the bulk electrolyte. The procedure is based on the work of Corbett et al. and Mateos et al.,[88, 89], and at least six samples were prepared and analyzed for each photoresist type. Photoresist samples were spin-coated as thin films on a 4 × 5 mm PEEK sample holder, and the films were cured overnight at room temperature before analysis. The film-coated sample holder was mounted on the z-axis stage of a commercially available electroosmotic flow mapping dip cell designed for ζ potential measurements of solid samples (ZEN1020, Malvern) and positioned between two palladium plate electrodes (Supporting Information, Figure S58). All the experiments were performed with tracer particles (~0.01 wt %) dispersed in a solution of 0.4 mM $[Ru(bpy)_3]^{2+}$ and 2.4 mM oxalic acid in 0.1 M H_2SO_4 corrected to pH 7 by dropwise addition of 10 M NaOH solution. Initially, the laser–surface distance was zeroed by identifying the minimum vertical position at which the beam, in forward scattering mode, is no longer blocked by the sample. The mobility (direction and magnitude) of the tracer particles was subsequently mapped at six distinct vertical positions as the sample stage was incrementally displaced (125 µm steps) from the beam. Mobility measurements, based on phase changes, were conducted at five vertical positions, with each measurement comprising 15 sub-runs and a delay time of 15 s. Given that, unlike in the case of emulsions, the solid ζ is inferred indirectly through the impact of electroosmotic flows on the electrophoretic mobility of the tracer, it is also necessary to assess the electrophoretic mobility at a vertical position where electroosmosis is minimal. This condition was met by conducting fast field reversal (FFR) measurements with the laser beam positioned 1 mm from the photoresist sample.

Contact angle measurements. Water contact angle values of photoresist films were determined at room temperature, in ambient air and with a commercial contact angle goniometer (model CAM101, KSV Instruments, Finland, Supporting Information, Figure S59). Samples were prepared in triplicate, with at least four separate spots being measured for each sample. Glassy carbon plates (25 mm diameter, 3 mm thick, Ted Pella, Inc., United States) were polished sequentially with 0.3 and 0.05 µm alumina slurry (20 min in total), sonicated in water for 10 min, then dried under a flow of nitrogen gas. NLOF 2035 and SU-8 2002 photoresists were spin-coated onto the polished carbon plates at 1500 and 4000 rpm, respectively. The photoresist films were baked for 5 min at 110 °C and then left to stand for at least 5 min prior to the contact angle measurement. Milli-Q™ water droplets of approximately 5 µL were dispensed onto the samples. Sample imaging and analysis were performed using the CAM100 v2.01 software.

Dielectric constant measurements. Dielectric constants were estimated through capacitance measurements. SU-8 2002 and NLOF 2035 photoresist films were spin–coated onto polished and clean glassy carbon plates (cleaning procedure as for the contact angle measurements section) selecting rotation speeds of 1500 rpm and 4000 rpm, respectively. Spin–coated glassy carbon plates were baked for 5 min at 110 °C. The PTFE cell for film capacitance measurement is shown in Supporting Information (Figure S60). Samples were prepared in triplicate and measurements were performed at 22 ± 1 °C. Approximately 1 mL of Galinstan (Ga/In/Sn, 62:22:16 wt %, Thermo Fisher Scientific, United States), a liquid metal alloy of gallium, indium, and tin, was used as the "soft" top electrical contact of the glassy carbon–photoresist–Galinstan capacitor.[90] Galinstan was stored under an inert atmosphere prior to use. Back contact with the glassy carbon electrode was ensured by pressing its back against a copper plate, while a platinum wire (0.5 mm diameter) was used to contact the top portion of the Galinstan layer. Capacitance values were collected using a four-wire precision LCR meter (E4980A, Keysight, Keysight Technologies Australia Pty Ltd) at frequencies of 1, 10, and 100 kHz, selecting 0 V as DC bias and 100 mV as AC bias. The calculated photoresist dielectric constant was tabulated in Table S5 (Supporting Information)

Hydrodynamic ECL experiments. ECL measurements at a platinum rotating disk electrode (RDE, 5.0 mm disk outer diameter, 12.0 mm PTFE shroud outer diameter, Pine Research Instrumentation, Inc., United States) were performed inside a grounded and light-proof Faraday cage using a stepped voltage waveform supplied by a source measure unit (SMU, Keysight, B2902A, Keysight Technologies Australia, Pty Ltd) to control the speed of the RDE rotator. A glass cell with a capacity of ~15 mL was used for the experiments (Supporting Information, Figure S61). The cell was filled with the solution containing $[Ru(bpy)_3]^{2+}$ (4 mM) and oxalic acid (24 mM) in H_2SO_4 (0.1 M) corrected to pH 6 through dropwise addition of aqueous NaOH (10 M). The cell was placed inside the Faraday cage and left to stand for 5 min prior to the measurement. The photometric detector (IPR–T TS2, Inphora Inc., United States) was placed underneath the cell holder as shown in Figure S61 (Supporting Information) and connected to a universal trans–impedance signal amplifier (P–9202–5, Gigahertz-Optik GmbH, Germany). The voltage output of the amplifier was logged with a benchtop multimeter (Keithley 2701 data acquisition system, Keithley Instruments, Inc., United States). The platinum RDE served as the working electrode, a platinum coil as the counter electrode, and a "leakless" Ag|AgCl in 3.4 M KCl as the reference electrode. The photodiode, amplifier, and voltage logging started approximately 30 s before the electrolysis. The working electrode bias was stepped from the open circuit to +1.4 V using a PalmSens4 potentiostat (PalmSens BV, Netherlands) to initiate the ECL reaction under stationary conditions. The rotation speed of the RDE was increased in 100 rpm increments, starting from 100 rpm and reaching up to 2000 rpm, with each step held for 20 s.

X-ray photoelectron spectroscopy (XPS). XPS measurements were conducted using an AXIS Ultra DLD spectrometer from Kratos Analytical Inc., Manchester, UK. The device was equipped with a monochromatic Al Kα radiation source (1486.6 eV) and a hemispherical analyzer with a 165 mm radius, operating in Fixed Analyzer Transmission (FAT) mode at approximately 2×10^{-8} Torr. Photoelectrons emitted at a 90° take-off angle were collected from a 300 × 700 μm analysis area. The pass energy of the narrow scans was set to 20 eV with a step size of 0.1 eV for F 1s (678–700 ev), O 1s (526−546 eV), N 1s (393−415 eV), Si 2p (96−108 eV), C 1s (278–302 eV), Cl 2p (196−216 eV), S 2p (159−179 eV), Ru 3p (446−506 eV) accumulating between 3 and 10 scans. Spectral regions shown in Figure S10–S13 (Supporting Information) were adjusted for clarity. Survey spectra were recorded over the 0−1120 eV range, in 0.5 eV steps and accumulating 3 scans at a pass energy of 160 eV. XPS data were processed using CasaXPS software, by applying a linear background subtraction. The spectral lines were asymmetric Lorentzian peak shapes (LA(50), 50% Gaussian and 50% Lorentzian). All reported energies are binding energies (eV) and were corrected by applying a rigid shift to align the carbon–carbon bound C 1s emission to 285.0 eV.

Conductivity measurements. Measurements of the ECL solutions' conductivity were performed inside a grounded Faraday cage using a two-electrode probe (ET902, eDAQ) connected to a precision LCR meter (E4980A, Keysight). Impedance measurements were taken at an AC frequency of 1 kHz, with a 20 mV excitation signal and no DC bias offset. The cell constant of the probe was calibrated prior to the measurements. A resistor–capacitor (R_s–C_s) series equivalent circuit model was used for impedance analysis.

Electrochemical reduction of levulinic acid (LA) to valeric acid (VA). Electrosynthetic experiments were conducted with a CHI650 workstation (CH Instruments, United States) and a three-electrode setup consisting of a glassy carbon plate (25 × 2 mm, high purity vitreous carbon planchet, Ted Pella, Inc., United States) as the working electrode, an Ag|AgCl (3.4 M KCl) "leakless" electrode (ET072-1, eDAQ, Australia) as reference, and a platinum wire (0.5 mm diameter) as counter electrode. The electrodes were housed in a custom-made PTFE cell with the cathode and anode compatments separated by a glass frit (Figure 49a). Prior to the electrosynthetic procedure, the glassy carbon plate was mechanically cleaned by polishing with alumina slurry (aluminum oxide suspended in water) on micro cloth pads (10 min with a 0.3 μm slurry, followed by 10 min with a 0.05 μm slurry). The electrode geometric area was defined by a 25 mm (inner diameter) nitrile O-ring. The clean GC electrode was then either left unmodified or photolithographically patterned with NLOF 2035 features (50 μm-wide squares separated by 60 μm gaps of clean GC surface). The electrosynthetic conversion of levulinic acid (LA) into valeric acid (VA) was performed by applying a constant potential of −1.8 V (chronoamperometry) for one hour, at room temperature (22 ± 1 °C). The working electrode compartment (cathode) was filled with 5 mL of aqueous 0.5 M H_2SO_4 containing

0.2 M LA. The counter electrode compartment (anode) contained 3 mL of 0.5 M H_2SO_4. Experiments were conducted without external stirring.

Chromatographic analysis. Quantitative analysis of the valeric acid (VA) produced from the electrolysis of levulinic acid (LA) was conducted via high-performance liquid chromatography (HPLC) on a Shimadzu (Shimadzu Corporation, Japan) system consisting of a DGU-20A degassing unit, a LC-20AD liquid chromatography pump, a SIL-20AC autosampler, a CTO-20AC column oven, a SPD-M20A photodiode array detector, a RID-20A refractive index detector, and a CMB-20A communication BUS module. The HPLC was fitted with a C18 column (Pursuit, Agilent Technologies, United States, 150 × 2 mm, 5 µm particle size, reverse phase) and the samples were eluted with a mobile phase composed of 10% acetonitrile and 90% water. The flow rate was set to 0.2 mL min^{-1}, and the column temperature was maintained at 30 °C during elution. Sample elution was monitored at 212 nm. The total run time was 30 min, preceded by a 2-minute equilibration period before injection. The injection volume was 5 µL. All samples were filtered through a 0.45 µm syringe PTFE filter before analysis. Chromatograms were processed through the Postrun Analysis module part of the LabSolutions software suite (Shimadzu Corporation, Japan). Retention times were verified by co-injection with pure standards.

Supporting Figures

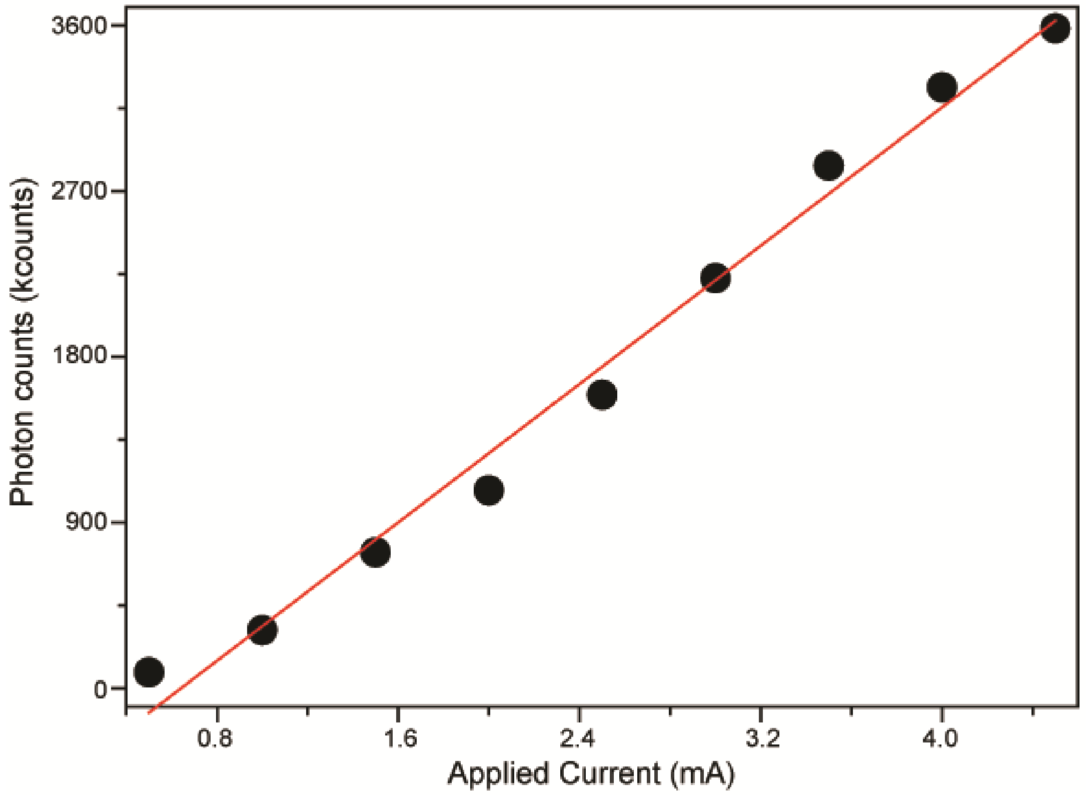

Figure S1. Relationship between applied current and ECL photon counts. Photon counting experiments were performed during galvanostatic experiments at ITO electrodes, and the applied current varied between 0.5 and 4.5 mA. The ECL solution contained $[Ru(bpy)_3]^{2+}$ (4 mM) and oxalate (24 mM) in aqueous H_2SO_4 (0.1 M, corrected to pH 6). Experimental details are outlined in the main text experimental section and the experiment setup is in the Supporting Information, Figure S56.

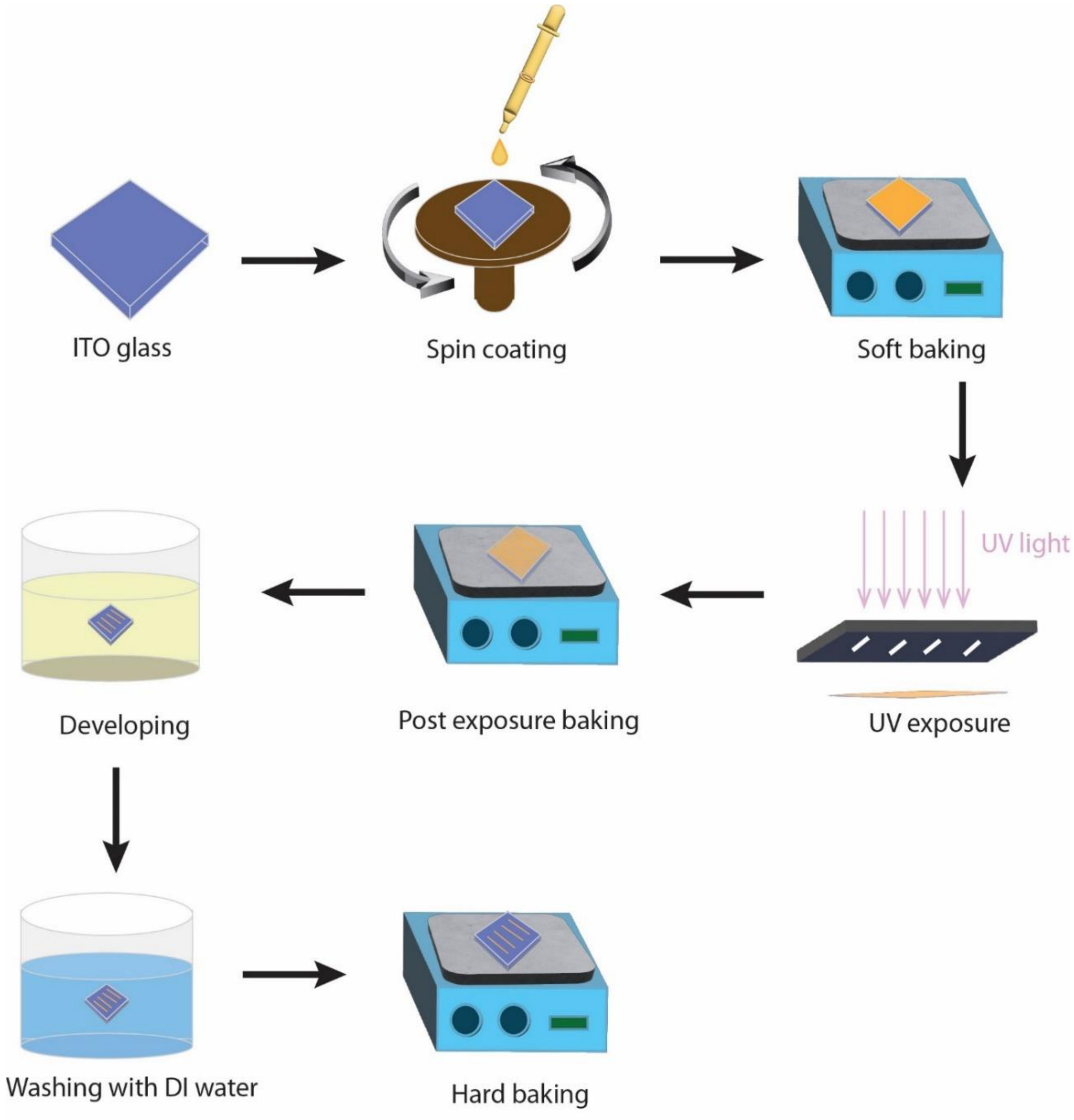

Figure S2. Photolithographic deposition of photoresists (NLOF 2035 and SU-8 2002) patterns on ITO-glass electrodes. Schematic overview of the photolithography process, with details on the materials, equipment, and conditions, described in the main text experimental section. The process was performed inside a clean room kept at a temperature of 22 ± 1 °C.

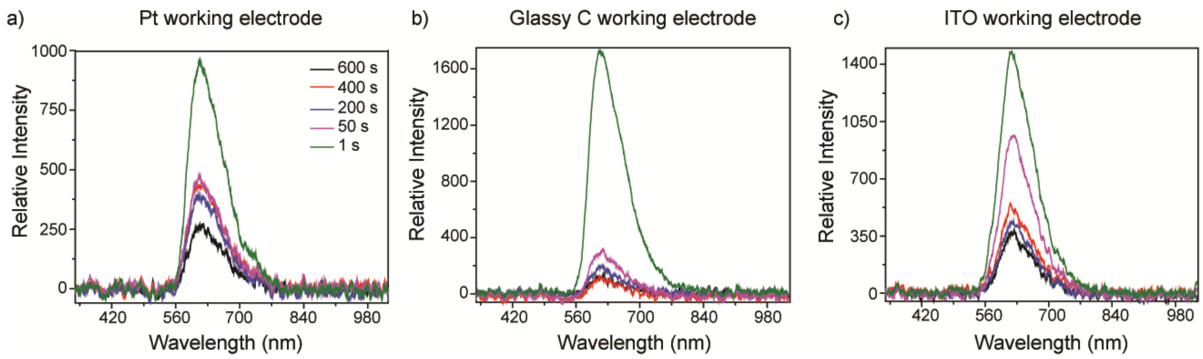

Figure S3. Spectroelectrochemical characterization of the ECL on different electrodes. The ECL solution was prepared as described in the main text experimental section. Two different ECL cells were used, depending on the working electrode material and geometry. For the data shown in (a), the ECL cell was a three-electrode, single-compartment 10-mm path-length quartz cuvette (10×10×40 mm, L×W×H) fitted with the Ag/AgCl "leakless" reference electrode described in the main experimental section, a platinum wire (0.5 mm diameter wire, 99.99+%, Goodfellow, Cambridge Limited) serving as counter electrode, and a platinum mesh (~7 × 6 mm overall size, EF-1355 SEC-C Gauze, 80 mesh, wire diameter of 80 µm, BASi) as working electrode. The Pt mesh was pre-cleaned by repeated cyclic voltammetric scans (at least 500 cycles) in 0.5 M H_2SO_4 with the potential swept from –0.4 V to 1.2 V at a scan rate of 0.5 V s^{-1}. For the data in (b) and (c) we used a back microscopy electrochemical flow cell (Redoxme AB, Sweden) although the solution was not circulated during the experiments. (b) A glassy carbon electrode (3 mm thick, 25 mm diameter, Ted Pella, Inc., United States) served as the working electrode, an Ag/AgCl "leakless" as the reference electrode, and a platinum wire as the counter electrode. (c) An ITO electrode (25 × 25 × 1 mm, with a sheet resistance of 7–10 ohms per square, Guangzhou Lepond Glass Co., Ltd., China) was used as the working electrode, a platinum wire as auxiliary electrode (35H X 15 0.6/235 mm, BASi, United States) and an Ag/AgCl "leakless" as reference electrode. Potentiostatic experiments were performed at room temperature (22 ± 1 °C) by applying a voltage bias of 1.4 V (versus reference) to the working electrode. The ECL emission spectra were acquired through a UV grade fused silica plano-convex lens (LA4647, Thorlabs) interfaced with an optical fiber patch cord (QP600-1-SR, Ocean Optics), using a linear silicon CCD array spectrometer (Flame-S-VISNIR-ES, Ocean Optics Inc., Florida). Emission spectra were recorded continuously, at an integration rate of 500 ms, for 600 s.

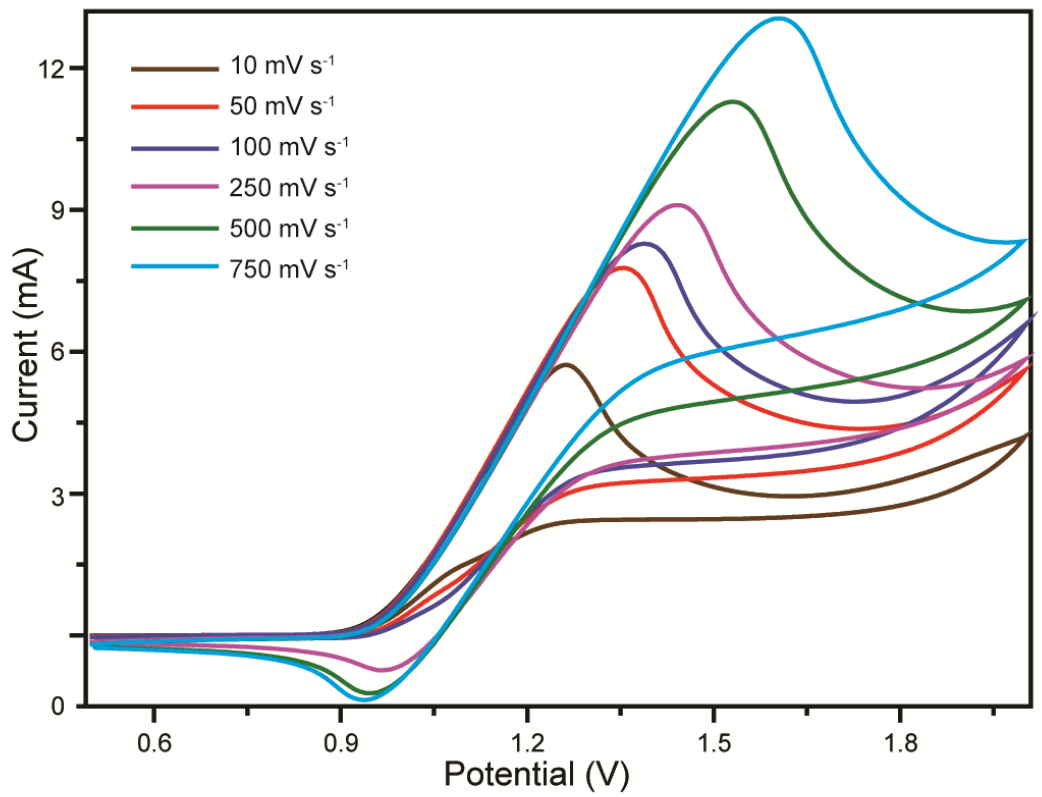

Figure S4. Electrochemical characterization on ITO of the ECL solution. The ECL solution was prepared as described in the main text experimental section. The pH value of the ECL electrolyte is ~6.2 and the cyclic voltammograms were collected at room temperature (22 ± 1 °C) using the same ECL cell as described for the ECL microscopy experiments. ITO slides served as the working electrode (50 × 50 × 1 mm, with a sheet resistance of 7–10 ohms per square) and the cyclic voltammetry potential window was from 0.5 V to 2.0 V (vs Ag|AgCl, 3.4 M KCl). The voltage sweep rate was varied between 10 and 750 mV s^{-1}.

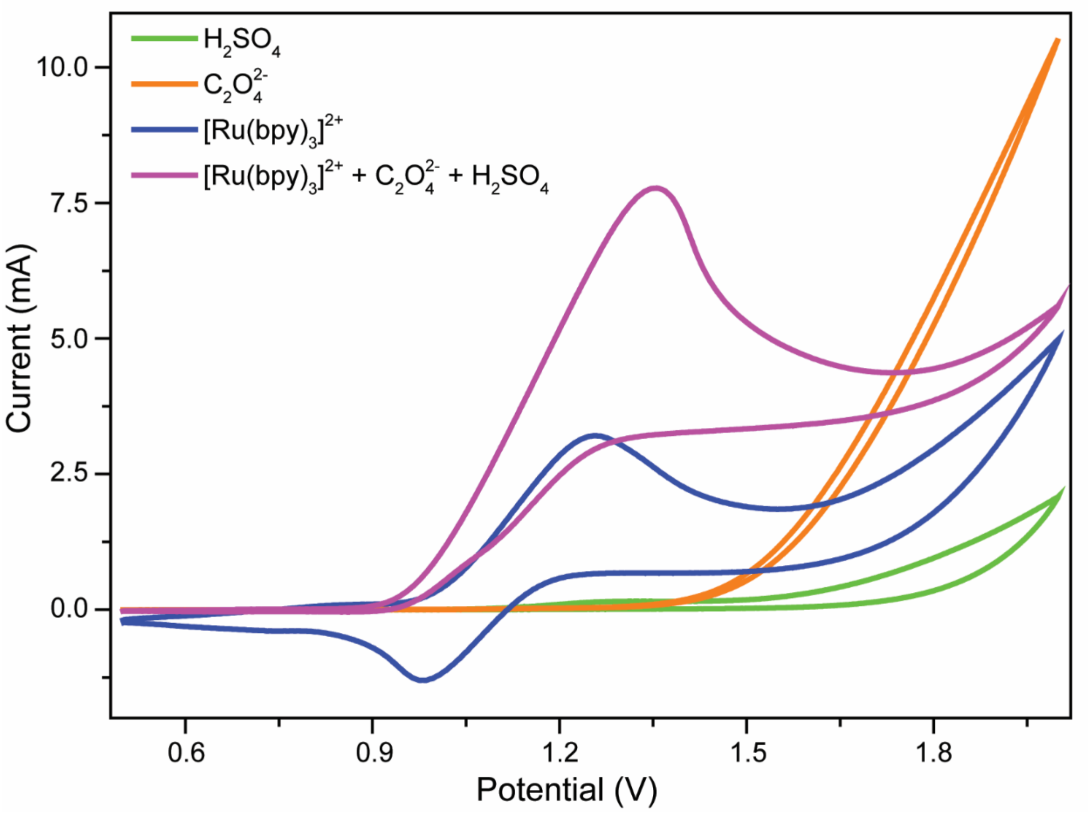

Figure S5. Voltammetry studies on ITO glass for the combined and isolated constituents of the ECL solution. Cyclic voltammograms of oxalic acid (24 mM), $[Ru(bpy)_3]^{2+}$ (4 mM), H_2SO_4 (0.1 M), and ECL solution recorded at pH 6 at the scan rate of 50 mV s^{-1}. The pH of all solutions was adjusted using 10 M NaOH and the measurements were carried out at room temperature (22 ± 1 °C). H_2SO_4 (0.1 M) served as the electrolyte in all cases. Measurements were carried out using an ITO glass electrode (50 × 50 × 1 mm, with a sheet resistance of 7–10 ohms per square) as the working electrode, over a 0.5–2.0 V (vs Ag|AgCl, 3.4 M KCl) potential window and with a platinum wire (0.5 mm diameter wire, 99.99+%, Goodfellow Cambridge Limited) as the counter electrode. The data provide insights into the electrochemical behavior and interactions of the individual and combined components. No clear Faradaic features can be observed when for experiments with only H_2SO_4 or with only oxalic acid and electrolyte. With $[Ru(bpy)_3]^{2+}$ both oxidation and reduction waves can be observed, while for the complete ECL solution only the oxidation feature can be seen as the oxidized $[Ru(bpy)_3]^{3+}$ is consumed by reductive species derived from oxalic acid.

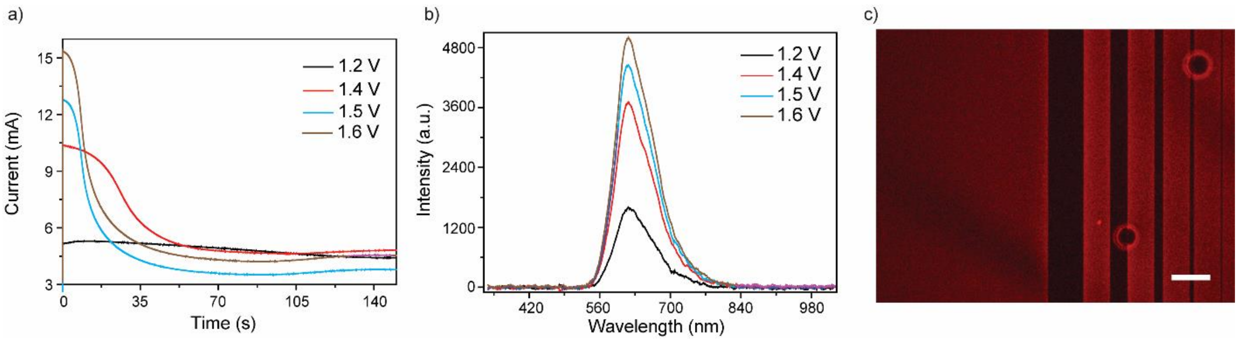

Figure S6. Spectroelectrochemical characterization of the ECL solution on ITO glass at different potentials. The ECL solution was 4 mM $[Ru(bpy)_3]^{2+}$ and 24 mM oxalic acid in 0.1 M H_2SO_4 at pH 6. ITO slides served as the working electrode (50 × 50 × 1 mm, with a sheet resistance of 7–10 ohms per square), a platinum wire (0.5 mm diameter wire, 99.99+%, Goodfellow Cambridge Limited) served as the counter electrode, and an Ag|AgCl “leakless” electrode was the potential reference. ECL starts to be sizable at 1.2 V. (a) Chronoamperograms recorded at different bias potential (1.2, 1.4, 1.5, and 1.6 V), and (b) the corresponding ECL emission spectra. (c) Representative ECL micrograph obtained at 1.5 V (scale bar is 400 µm). At an anodic bias of 1.5 V the stability of the lithographically fouled electrodes becomes poor and significant gas evolution is observed.

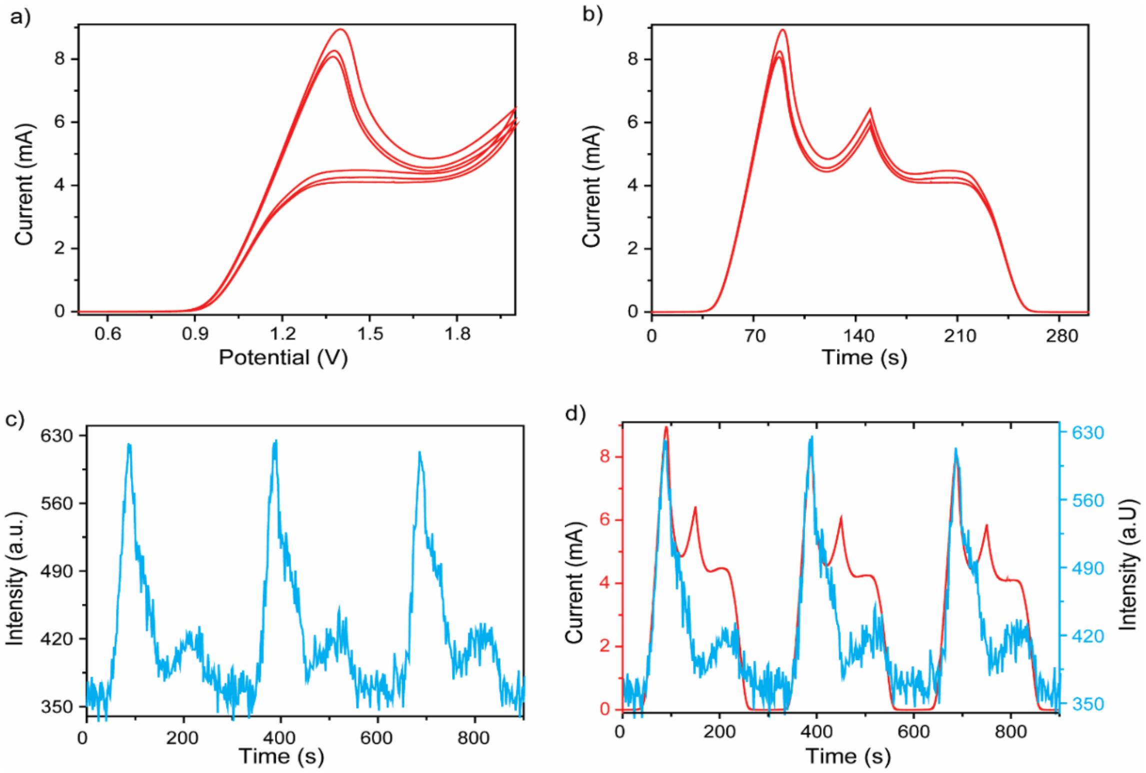

Figure S7. Electrochemical and spectroelectrochemical characterization of the ECL solution on ITO glass at low potential sweep rates. The ECL solution was prepared as described in the main text experimental section. The pH value of the ECL system is ~ 6.2 and the cyclic voltammograms were collected at room temperature (22 ± 1 °C) using the same ECL cell described for the ECL microscopy experiments. ITO electrode served as the working electrode (50 × 50 × 1 mm, with a sheet resistance of 7–10 ohms per square), and a platinum wire (0.5 mm diameter wire, 99.99+%, Goodfellow Cambridge Limited) served as the counter electrode. (a) Cyclic voltammetry was performed by sweeping the working electrode potential from 0.5 V to 2.0 V (vs Ag|AgCl, 3.4 M KCl) at a scan rate of 10 mV s^{-1} (3 cycles). (b) Cyclic voltammograms of panel (b) but plotted after the x-axis variable was converted from voltage bias to time. (c) ECL intensity data (at the wavelength corresponding to the ECL maximum) recorded during the voltametric experiments. The ECL integration time was of 1 s. (d) Superimposed ECL intensity and cyclic voltammetry data (as a function of time).

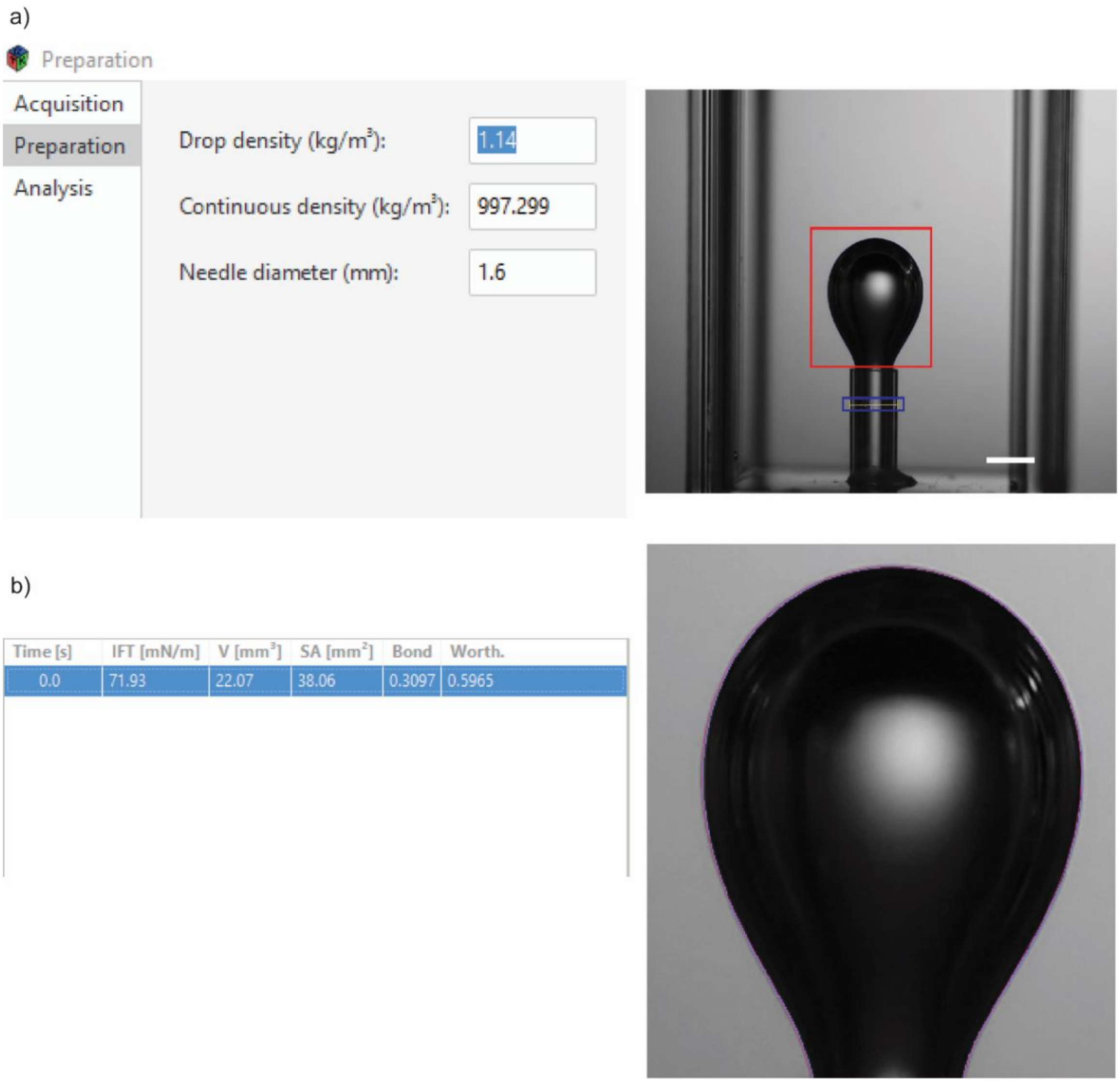

Figure S8. Representative surface tension measurement using the rising bubble method. (a) The red color rectangle marks the region of interest. The blue rectangle identifies the capillary (needle), and the purple color border defines the droplet's border as determined by the OpenDrop software (image analysis software for performing drop tensiometry measurements). The scale bar is 1.6 mm. (b) The software refines a model and suggests a value of surface tension according to the drop density, the continuous density, capillary diameter and drop shape. Before measuring the surface tension of the system, the capillary diameter was calibrated for bubbles in pure water at room temperature. Since the surface tension of water at a specific temperature can be obtained through literature data, the needle diameter can be cross-checked through the surface tension fitting. The needle diameter optimized for pure water surface tension was used to measure the surface tension of oxalic acid and ECL solutions. The experimental setup is further described and illustrated in Figure S57.

Table S1. The surface tension of water, oxalic acid in different pH values. The pH values (2 to 8) were adjusted by dropwise addition of aqueous 10 M NaOH and the ECL system prepared with a fixed concentration of $[Ru(bpy)_3]^{2+}$ (4 mM) varying the concentration of oxalic acid (12, 24, 48, 72, and 96 mM) and the pH was adjusted using dropwise addition of aqueous NaOH. Measurements were obtained at room temperature (~22 °C).

Sample name		Needle diameter (mm)	Surface Tension(mN m^{-1})	Volume (mm^3)	Temperature ˚C
Water	Bubble 1	1.6	71.91	21.63	23.0
	Bubble 2	1.6	71.96	22.03	23.0
	Bubble 3	1.61	71.97	21.81	23.0
	Bubble 4	1.6	71.92	21.67	23.0
	Bubble 5	1.6	71.92	21.03	23.0
	Bubble 6	1.6	71.95	22.2	23.0
	Bubble 7	1.59	71.97	20.95	23.0
	Bubble 8	1.59	71.97	21.29	23.0
	Bubble 9	1.6	71.95	21.44	23.0
	Bubble 10	1.6	71.93	21.63	23.0
**Average**		**1.6**	**71.95**	**21.57**	23.0
Oxalic pH 2	Bubble 1	1.6	71.95	20.16	23.0
	Bubble 2	1.6	71.57	21.4	23.0
	Bubble 3	1.6	71.71	19.86	23.0
	Bubble 4	1.6	72.13	20.28	23.0
	Bubble 5	1.6	72.3	19.9	23.0
	Bubble 6	1.6	71.91	21.73	23.0
	Bubble 7	1.6	71.96	19.79	23.0
	Bubble 8	1.6	72.13	21.56	23.0
**Average**		**1.6**	**71.95**	**20.79**	23.0
Oxalic pH 3	Bubble 1	1.6	71.49	19.43	23.0
	Bubble 2	1.6	71.59	21.01	23.0
	Bubble 3	1.6	71.36	21.32	23.0
	Bubble 4	1.6	71.44	21.31	23.0
	Bubble 5	1.6	71.67	21.07	23.0
	Bubble 6	1.6	71.54	20.76	23.0
	Bubble 7	1.6	71.65	20.82	23.0
	Bubble 8	1.6	71.53	20.99	23.0
**Average**		**1.6**	**71.64**	**20.91**	23.0
oxalic pH 4	Bubble 1	1.6	70.99	20.94	23.0
	Bubble 2	1.6	71.44	21.6	23.0
	Bubble 3	1.6	71.19	21.74	23.0
	Bubble 4	1.6	70.65	21.54	23.0
	Bubble 5	1.6	70.94	21.47	23.0
	Bubble 6	1.6	71.52	21.8	23.0
	Bubble 7	1.6	71.94	20.92	23.0
	Bubble 8	1.6	71.05	21.74	23.0
**Average**		**1.6**	**71.29**	**21.36**	23.0
Oxaic pH 5	Bubble 1	1.6	71.75	20.99	23.0
	Bubble 2	1.6	70.91	21.1	23.0
	Bubble 3	1.6	71.05	21.49	23.0

	Bubble 4	1.6	71.48	21.88	23.0
	Bubble 5	1.6	70.91	21.26	23.0
	Bubble 6	1.6	71.1	21.41	23.0
	Bubble 7	1.6	71.11	21.76	23.0
	Bubble 8	1.6	71.08	20.98	23.0
**Average**		**1.6**	**71.17**	**21.40**	23.0
Oxallic pH 6	Bubble 1	1.6	71.17	21.44	23.0
	Bubble 2	1.6	71.18	21.52	23.0
	Bubble 3	1.6	70.89	21.6	23.0
	Bubble 4	1.6	70.86	21.64	23.0
	Bubble 5	1.6	70.86	21.55	23.0
	Bubble 6	1.6	70.47	21.33	23.0
	Bubble 7	1.6	71.27	21.82	23.0
	Bubble 8	1.6	70.97	21.32	23.0
**Average**		**1.6**	**70.99**	**21.46**	23.0
Oxalic pH 7	Bubble 1	1.6	71.1	21.44	23.0
	Bubble 2	1.6	71.22	20.91	23.0
	Bubble 3	1.6	70.63	20.94	23.0
	Bubble 4	1.6	71.2	20.73	23.0
	Bubble 5	1.6	70.9	21.45	23.0
	Bubble 6	1.6	70.64	20.3	23.0
	Bubble 7	1.6	71.05	21.31	23.0
	Bubble 8	1.6	70.9	21.51	23.0
**Average**		**1.6**	**70.96**	**21.14**	23.0
Oxalic pH 8	Bubble 1	1.6	71.15	20.17	23.0
	Bubble 2	1.6	70.65	21.05	23.0
	Bubble 3	1.6	70.77	19.89	23.0
	Bubble 4	1.6	70.2	20.28	23.0
	Bubble 5	1.6	70.75	21.53	23.0
	Bubble 6	1.6	70.94	21.23	23.0
	Bubble 7	1.6	70.28	20.77	23.0
	Bubble 8	1.6	70.63	20.96	23.0
**Average**		**1.6**	**70.72**	**20.85**	23.0
4 mM Ru 12 mM oxalic	Bubble 1	1.6	72.68	21.03	23.0
	Bubble 2	1.6	72.48	21.83	23.0
	Bubble 3	1.6	72.47	21.31	23.0
	Bubble 4	1.6	72.39	21.63	23.0
	Bubble 5	1.6	72.52	1.04	23.0
	Bubble 6	1.6	72.42	22.08	23.0
	Bubble 7	1.6	72.49	21.34	23.0
	Bubble 8	1.6	72.6	21.53	23.0
	Bubble 9	1.6	72.47	20.16	23.0
	Bubble 10	1.6	72.43	20.56	23.0
**Average**		**1.6**	**72.50**	**19.25**	23.0
4 mM Ru 24 mM Oxalic	Bubble 1	1.6	72.29	20.81	23.0
	Bubble 2	1.6	72.33	22.36	23.0
	Bubble 3	1.6	72.15	20.22	23.0

	Bubble 4	1.6	72.27	22.52	23.0
	Bubble 5	1.6	72.31	20.56	23.0
	Bubble 6	1.6	72.13	21.5	23.0
	Bubble 7	1.6	72.21	20.51	23.0
	Bubble 8	1.6	72.19	21.28	23.0
	Bubble 9	1.6	72.13	21.71	23.0
	Bubble 10	1.6	72.17	21.08	23.0
**Average**		**1.6**	**72.22**	**21.26**	23.0
4 mM Ru 48 mM Oxalic	Bubble 1	1.6	71.79	22.26	23.0
	Bubble 2	1.6	71.81	20.88	23.0
	Bubble 3	1.6	72.08	21.47	23.0
	Bubble 4	1.6	71.93	22.27	23.0
	Bubble 5	1.6	72.06	21.81	23.0
	Bubble 6	1.6	71.9	20.74	23.0
	Bubble 7	1.6	71.95	21.52	23.0
	Bubble 8	1.6	71.93	20.23	23.0
	Bubble 9	1.6	71.96	17.39	23.0
	Bubble 10	1.6	71.92	21.62	23.0
**Average**		**1.6**	**71.93**	**21.02**	23.0
4 mM Ru 72 mM Oxalic	Bubble 1	1.6	71.82	21.19	23.0
	Bubble 2	1.6	71.83	19.72	23.0
	Bubble 3	1.6	71.52	20.28	23.0
	Bubble 4	1.6	71.58	21.51	23.0
	Bubble 5	1.6	71.66	21.92	23.0
	Bubble 6	1.6	71.78	20.7	23.0
	Bubble 7	1.6	71.69	20.74	23.0
	Bubble 8	1.6	71.86	20.8	23.0
	Bubble 9	1.6	71.67	21.54	23.0
	Bubble 10	1.6	71.82	22.3	23.0
**Average**		**1.6**	**71.72**	**21.07**	23.0
4 mM Ru 96 mM Oxalic	Bubble 1	1.6	71.56	18.34	23.0
	Bubble 2	1.6	71.42	20.75	23.0
	Bubble 3	1.6	71.49	21.18	23.0
	Bubble 4	1.6	71.31	19.39	23.0
	Bubble 5	1.6	71.37	20.82	23.0
	Bubble 6	1.6	71.51	20.85	23.0
	Bubble 7	1.6	71.43	20.59	23.0
	Bubble 8	1.6	71.6	21.04	23.0
	Bubble 9	1.6	71.64	21.17	23.0
	Bubble 10	1.6	71.55	20.89	23.0
**Average**		**1.6**	**71.49**	**20.50**	23.0
TPrA pH 10	Bubble 1	1.6	53.16	14.45	22.0
	Bubble 2	1.6	53.18	14.7	22.0
	Bubble 3	1.6	53.1	15.61	22.0
	Bubble 4	1.6	53.31	15.55	22.0
	Bubble 5	1.6	53.23	15.77	22.0
	Bubble 6	1.6	53.31	13.71	22.0

	Bubble 7	1.6	53.12	14.73	22.0
	Bubble 8	1.6	53.27	15.79	22.0
	Bubble 9	1.6	53.26	15.51	22.0
	Bubble 10	1.6	53.16	15.75	22.0
**Average**		**1.6**	**53.21**	**15.16**	**22.0**

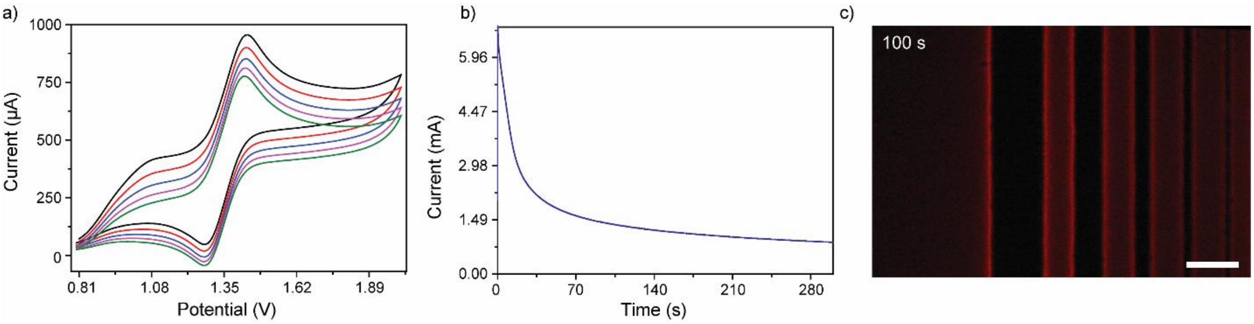

Figure S9. Electrochemical characterization and ECL mapping in the presence of surface enrichment effects (surface-active ECL co-reactants). Unlike all the other experiments of this study, in this control the ECL solution was 1 mM $[Ru(bpy)_3]^{2+}$ as the luminophore, 10 mM TPrA (surface active molecule) as ECL co-reactant in aqueous sodium dihydrogen orthophosphate (0.15 M) at pH 10. The pH was adjusted by adding 10 M NaOH. The working electrode was ITO glass photolithographed with SU-8 2002 photoresist (50 × 50 × 1 mm, with a sheet resistance of 7–10 ohms per square), the counter electrode was a platinum wire (0.5 mm diameter wire, 99.99+%, Goodfellow Cambridge Limited) and an Ag|AgCl "leakless" electrode served as the potential reference electrode. (a) Five cyclic voltammetry cycles were collected with the working electrode bias swept from 0.80 V to 2.0 V (Ag|AgCl, 3.4 M KCl) at the sweep rate of 10 mV s^{-1}. Since the oxidation rate peaks near 1.4 V, ECL experiments were done at that potential. (b) Chronoamperogram obtained at an applied working electrode bias of 1.4 V (Ag|AgCl, 3.4 M KCl), and (c) ECL micrograph obtained after 100 s of the working electrode bias having been stepped from open circuit to 1.4 V. The scale bar is 400 µm.

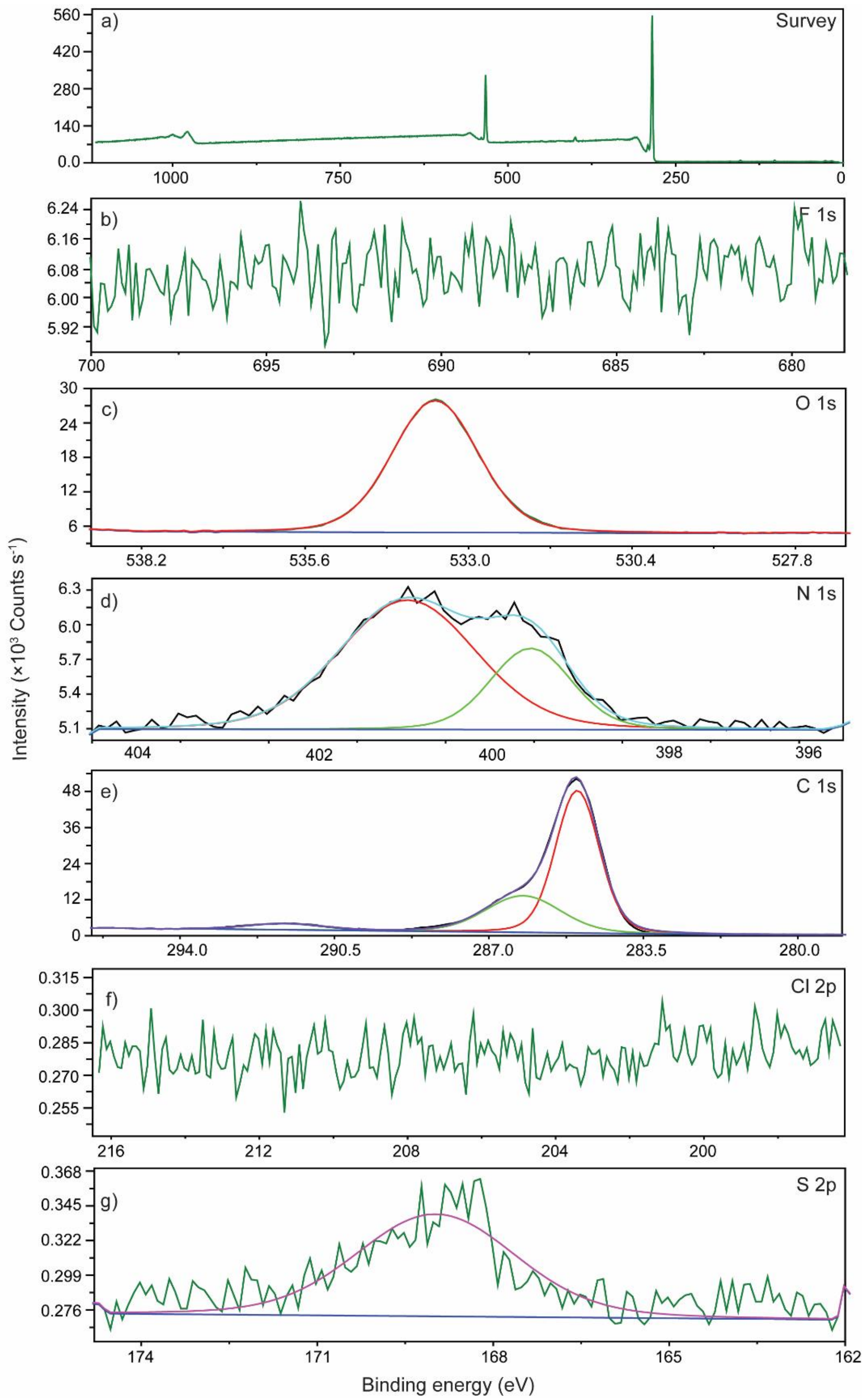

Figure S10. X-ray photoelectron spectroscopy (XPS) analysis of NLOF 2035 films. XPS measurements conducted to investigate the elemental composition of the photoresist film. Films of the photoresist (thickness of 3 µm) were deposited through spin coating on transparent ITO glass, selecting a rotating speed of 3500 rpm. (a) XPS survey spectrum, and high-resolution spectra of the (b) F 1s, (c) O 1s, (d) N 1s, (e) C 1s, (f) Cl 2p, and (g) S 2p regions.

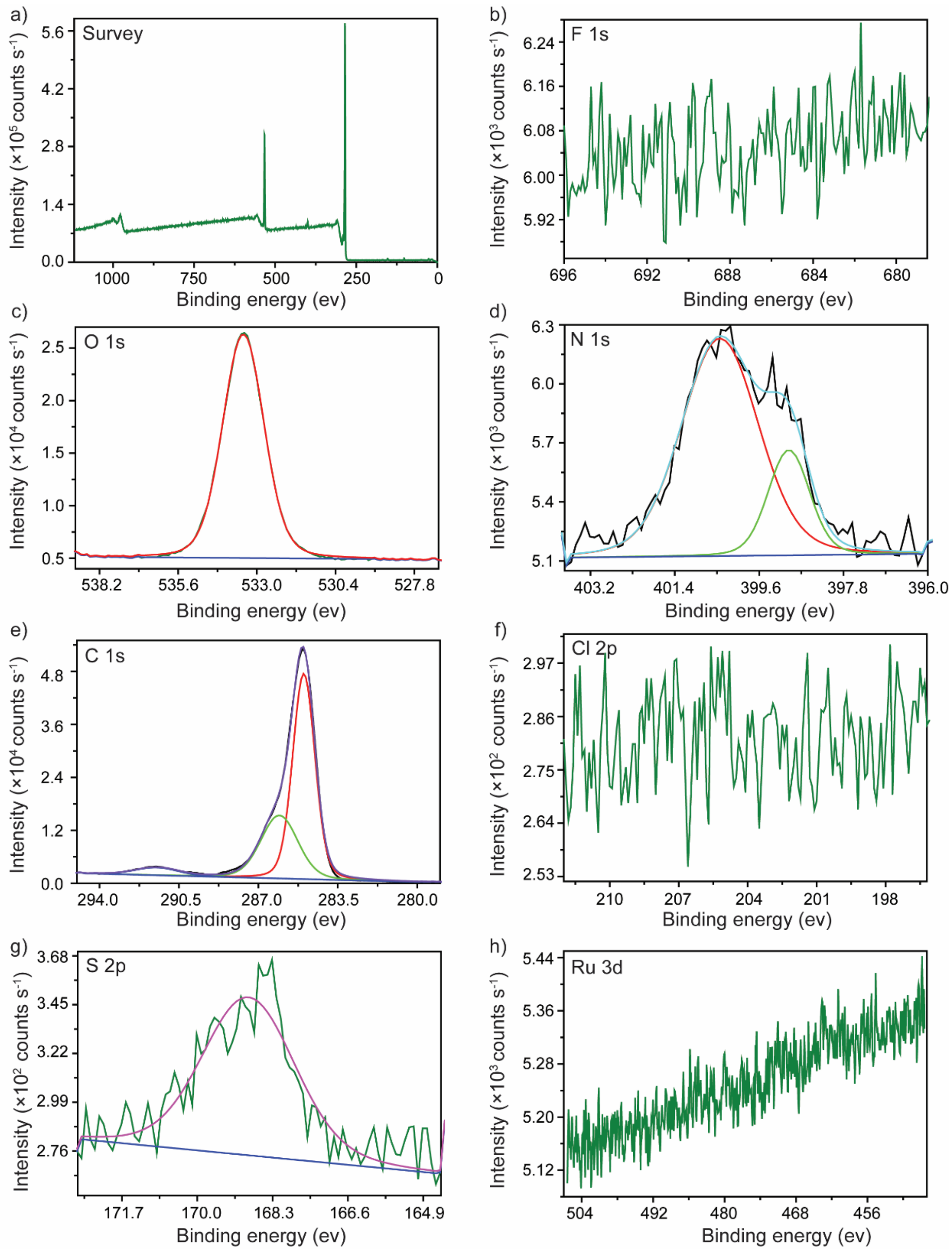

Figure S11. XPS analysis of NLOF 2035 photoresist films incubated with the ECL solution. XPS analysis to assess the adsorption of oxalic acid and $[Ru(bpy)_3]^{2+}$ on NLOF 2035 films. The photoresist-coated ITO slide (film thickness of 3 µm) was immersed in the ECL solution and rested in it for at least 15 min. The sample was then rinsed copiously with distilled water, and then analyzed by XPS. (a) XPS survey spectrum and narrow scans of the (b) F 1s, (c) O 1s, (d) N 1s, (e) C 1s, (f) Cl 2p, (g) S 2p, and (h) Ru 3d regions.

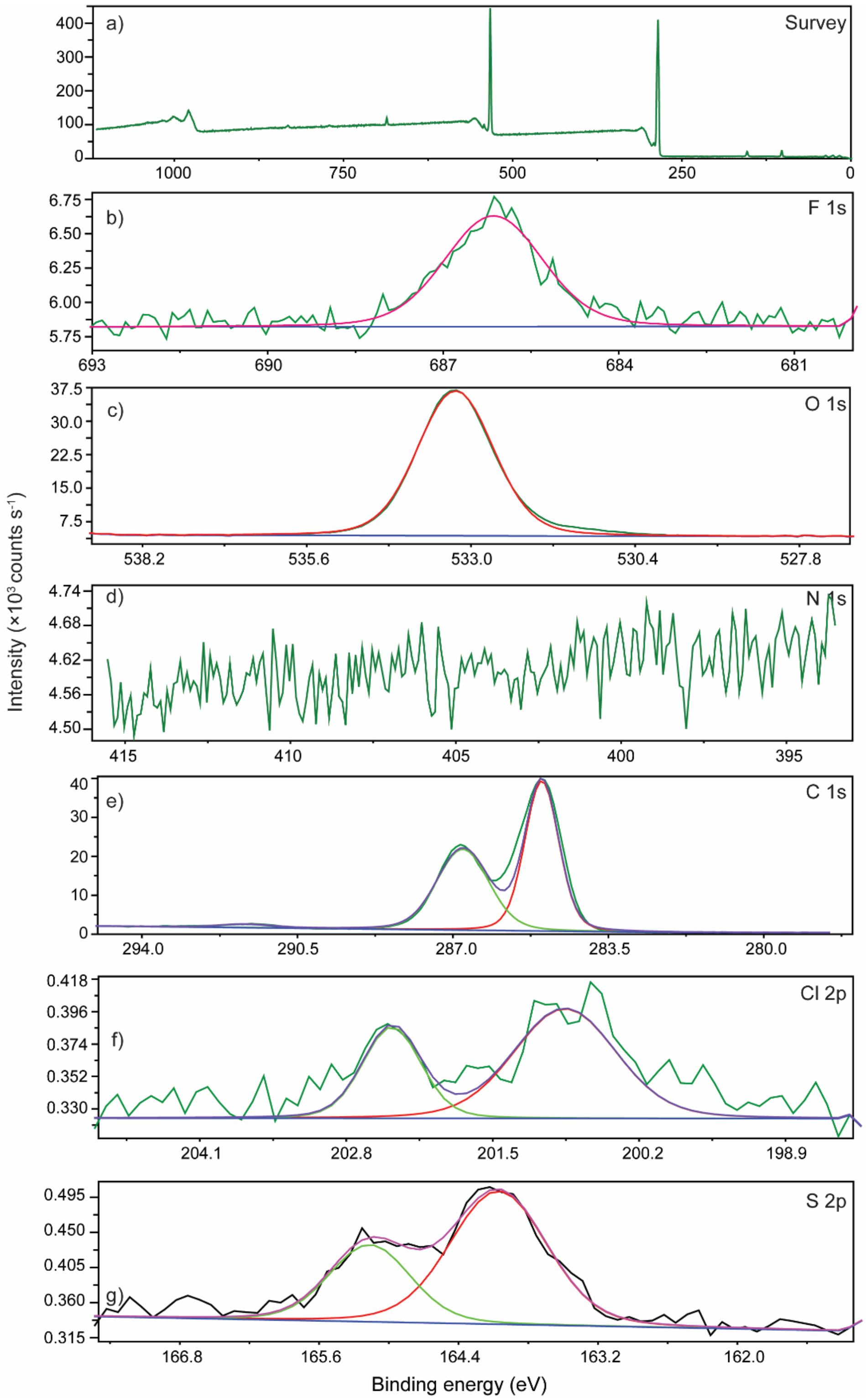

Figure S12. XPS analysis of SU-8 2002 films. XPS measurements conducted to investigate the photoresist elemental composition. Films of the photoresist (thickness of 3 µm) were deposited through spin coating on transparent ITO glass using a rotating speed of 100 rpm. (a) XPS survey spectrum, and high-resolution spectra of the (b) F 1s, (c) O 1s, (d) N 1s, (e) C 1s, (f) Cl 2p, and (g) S 2p regions.

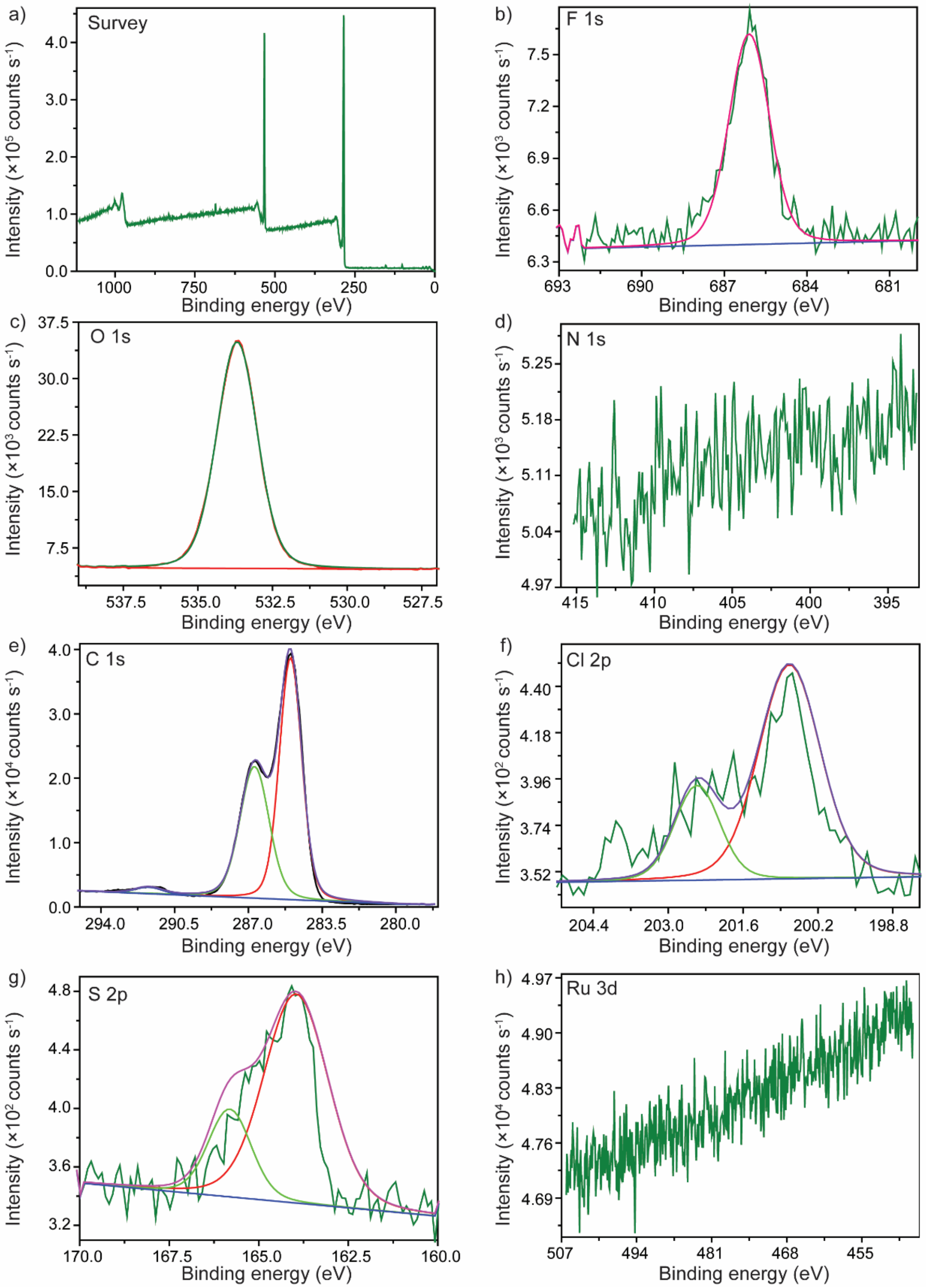

Figure S13. XPS analysis of SU-8 2002 photoresist films incubated with the ECL solution. XPS analysis to assess the adsorption of oxalic acid and $[Ru(bpy)_3]^{2+}$ on SU-8 2002 films. The photoresist-coated ITO slide (film thickness of 3 µm) was immersed in the ECL solution and rested in it for at least 15 min. The sample was then removed from the solution and rinsed copiously with distilled water prior to the XPS measurement. (a) XPS survey spectrum and narrow scans of the (b) F 1s, (c) O 1s, (d) N 1s, (e) C 1s, (f) Cl 2p, (g) S 2p, and (h) Ru 3d regions.

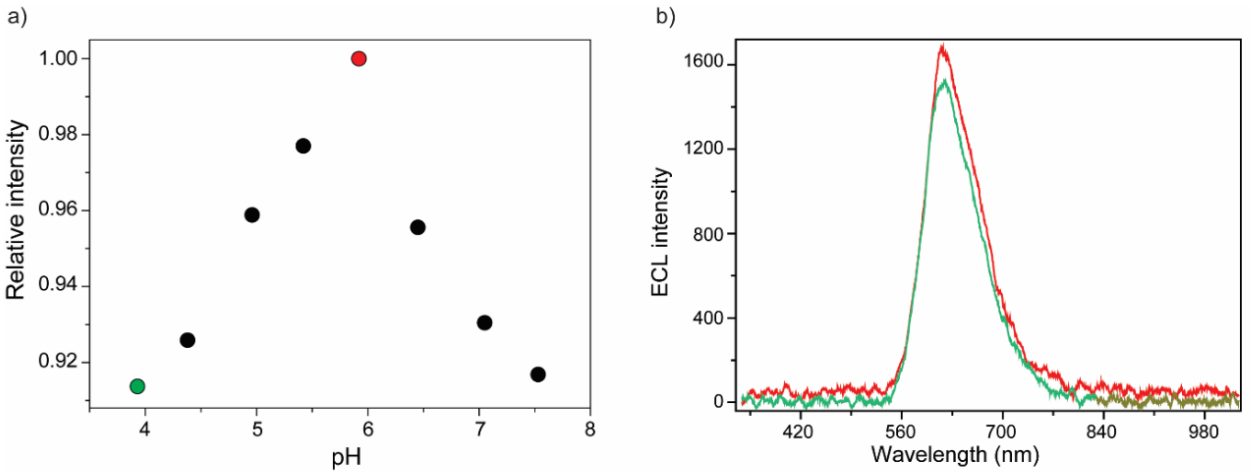

Figure S14. Spectroelectrochemical measurements towards ECL optimization through electrolyte pH changes. The electrolyte pH plays a decisive role in determining ECL intensities and as shown in (a) the brightest ECL was observed at a pH close to 6, in agreement with previous reports by Bard and Rubinstein.[91] The relatively poor ECL emission in acidic pH values can be attributed to less efficient redox reactions due to co-reactants not being in their optimal protonation state for the ECL mechanism. As the pH increases beyond 6, a decrease in ECL intensity is likely due to competing side and solvent reactions. The ECL solution contained 4 mM $[Ru(bpy)_3]^{2+}$ and 24 mM oxalic acid in 0.1 M H_2SO_4. The pH was adjusted by dropwise addition of 10 mM NaOH. The relative intensity plotted in (a) refers to the ECL intensity sampled after 1 s of applying the anodizing voltage (1.4 V vs Ag|AgCl, 3.4 M KCl). The relative intensity was calculated by dividing the observed intensity for the overall highest ECL intensity measured (pH 6). (b) Representative ECL spectra for electrolytes of pH either 3.9 (green) or 5.9 (red). Experiments were carried out at room temperature, with a working ITO glass electrode (50 × 50 × 1 mm, with a sheet resistance of 7–10 ohms per square) and using a platinum wire (0.5 mm diameter wire, 99.99+%, Goodfellow Cambridge Limited) as counter electrode.

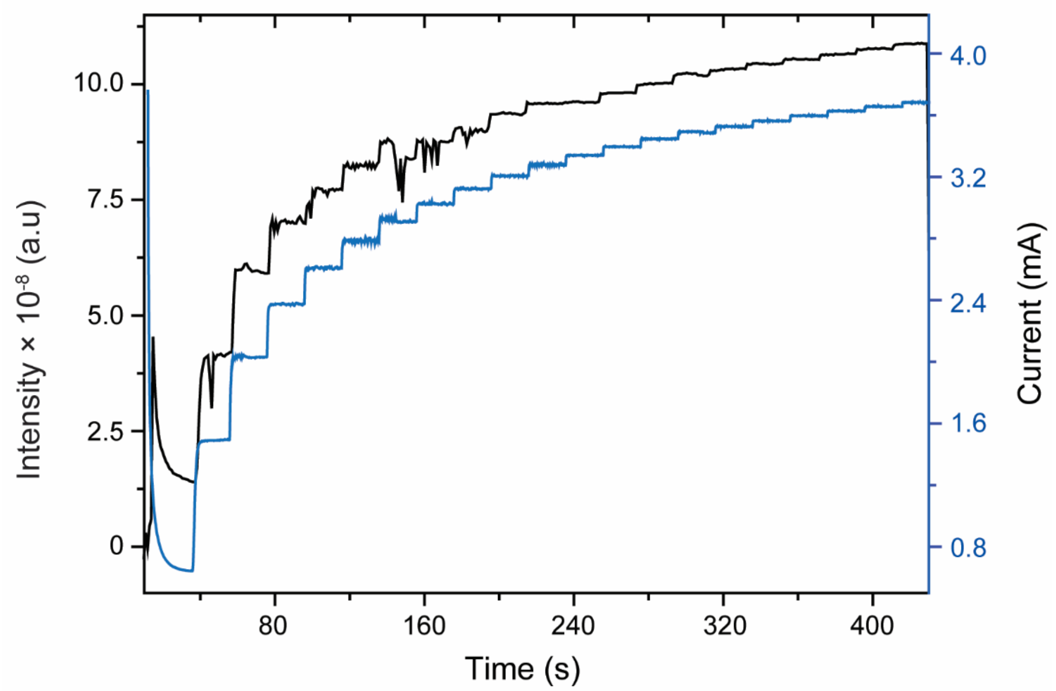

Figure S15. Simultaneous hydrodynamic rotating disk electrode (RDE) and ECL intensity experiments. Electrolyte stirring effects on the electrochemiluminescent emission from an anodized solution of 4 mM $Ru(bpy)_3]^{2+}$ and 24 mM oxalate in aqueous H_2SO_4 (0.1 M, corrected to pH 6). The measurements were conducted with the platinum disk electrode (RDE) rotation speed being first increased from stationary to 100 rpm, and then on to 2000 rpm in 100 rpm increments. The plot superimposes the measured ECL intensity and the corresponding hydrodynamic current. The platinum RDE served as the working electrode, a platinum coil as the counter electrode, and a "leakless" Ag|AgCl in 3.4 M KCl as the reference electrode. The working electrode bias was stepped from the open circuit to +1.4 V while the system was still quiescent. The experimental setup is further described in Figure S61.

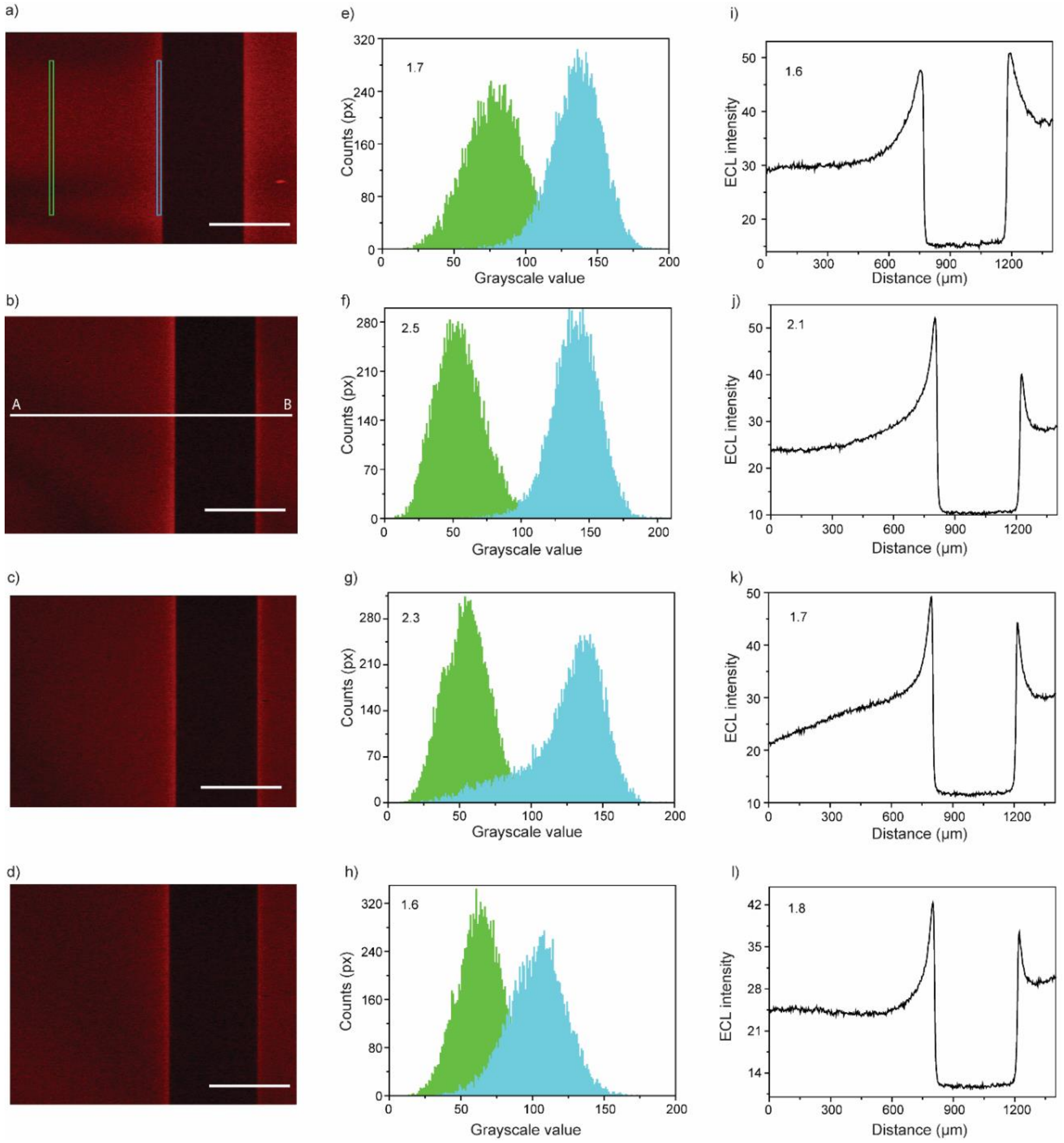

Figure S16. Estimation of ECL enhancement factors (near insulators) through histogram analysis and cross-sectional line profiles. The ECL solution was prepared as mentioned in the experimental section and was 4 mM $[Ru(bpy)_3]^{2+}$ and 24 mM oxalate in aqueous H_2SO_4 (0.1 M, corrected to pH 6). The working electrode bias was +1.4 V (Ag|AgCl in 3.4 M KCl). (a–d) Representative ECL micrographs, from independent experiments, sampled at the electrolysis time corresponding to the maximum recorded near-insulator enhancement (as assessed from ECL histograms). This electrolysis time was 80, 110, 100, and 90 s in (a–d), respectively. The green box shown in (a) represents the area of interest used to build ECL histograms representative of the "clean" ITO region (i.e. away from the photoresist line). The blue box indicates the "near-insulator" region of interest. The scale bars are 400 µm. (e–h) Corresponding "clean" and "near-insulator" histograms for the micrographs in (a–d). The ECL enhancement factor (fold increase) near versus away from the insulating feature is indicated in figure (upper left corner). (i–l) Corresponding ECL intensity plot profiles, sampled in correspondence of the A–B line (shown in (b) only) and the corresponding near-insulator enhancement factors.

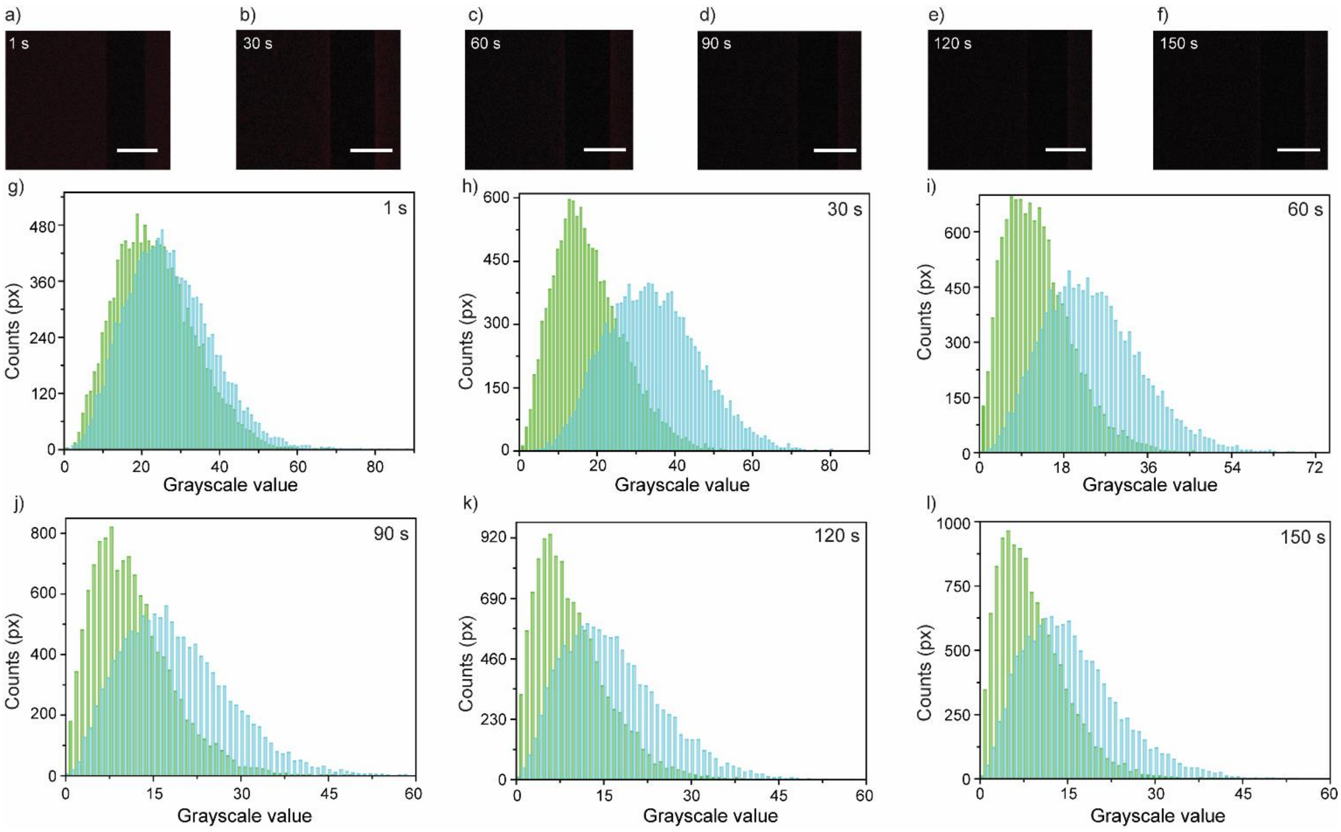

Figure S17. Representative ECL micrographs and region-of-interest ECL intensity histograms. Representative ECL microscopy data (a–f), and their descriptive (histograms) data analysis (g–l) used to compute the ECL augmentation versus luminophore concentration data as shown in Figure 3e of the main text. The ECL solution contained 1 mM $Ru(bpy)_3]^{2+}$ and 6 mM oxalate in aqueous H_2SO_4 (0.1 M, corrected to pH 6). ECL micrographs were collected at an ITO electrode (50 × 50 × 1 mm, with a sheet resistance of 7–10 ohms per square) using a Nikon inverted microscope (2× magnification) during potentiostatic (chronoamperometry) experiments with the working electrode bias set to 1.4 V (vs Ag|AgCl, 3.4 M KCl). The duration of the experiments was generally equal or greater than 150 s. A platinum wire (0.5 mm diameter wire, 99.99+%, Goodfellow Cambridge Limited) served as the counter electrode. The red channel histograms, built from the ECL data (micrographs shown in a–f) with the open-source image processing software Fiji, refer to specific regions of interest (ROI). The ROIs were in the shape of vertical rectangles, as shown in Figure 3a of the main text (10 × 1200 px dimensions). The scale bars in figure indicate 400 µm. The blue color data correspond to ECL intensity sampled in ROIs near the insulating object (dark vertical lines in the ECL micrographs), while green color data corresponds to the ECl emission sampled away from the insulator (see Figure 3a). The intensity data shown in Figure 3e are taken as the area under the histogram, obtained by integrating the counts over the relevant range. The electrolysis time is shown in each panel (both in the micrographs as well as in the histograms).

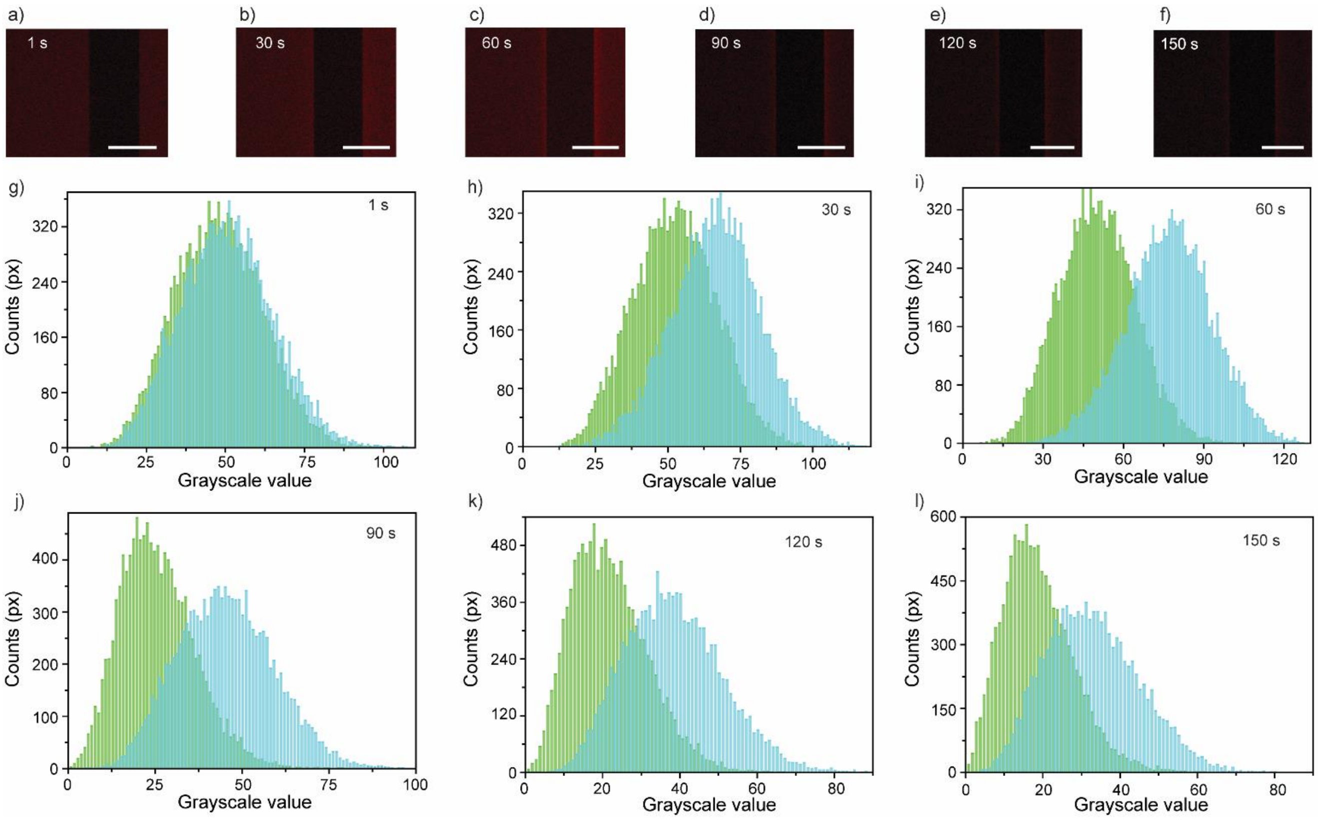

Figure S18. Representative ECL micrographs and region of interest ECL intensity histograms. Representative ECL micrographs and region-of-interest ECL intensity histograms. Representative ECL microscopy data (a–f), and their descriptive (histograms) data analysis (g–l) used to compute the ECL augmentation versus luminophore concentration data as shown in Figure 3e of the main text. The ECL solution contained 2 mM $Ru(bpy)_3]^{2+}$ and 12 mM oxalate in aqueous H_2SO_4 (0.1 M, corrected to pH 6). ECL micrographs were collected at an ITO electrode (50 × 50 × 1 mm, with a sheet resistance of 7–10 ohms per square) using a Nikon inverted microscope (2× magnification) during potentiostatic (chronoamperometry) experiments with the working electrode bias set to 1.4 V (vs Ag|AgCl, 3.4 M KCl). The duration of the experiments was generally equal or greater than 150 s. A platinum wire (0.5 mm diameter wire, 99.99+%, Goodfellow Cambridge Limited) served as the counter electrode. The red channel histograms, built from the ECL data (micrographs shown in a–f) with the open-source image processing software Fiji, refer to specific regions of interest (ROI). The ROIs were in the shape of vertical rectangles, as shown in Figure 3a of the main text (10 × 1200 px dimensions). The scale bars in figure indicate 400 µm. The blue color data correspond to ECL intensity sampled in ROIs near the insulating object (dark vertical lines in the ECL micrographs), while green color data corresponds to the ECl emission sampled away from the insulator (see Figure 3a). The intensity data shown in Figure 3e are taken as the area under the histogram, obtained by integrating the counts over the relevant range. The electrolysis time is shown in each panel (both in the micrographs as well as in the histograms).

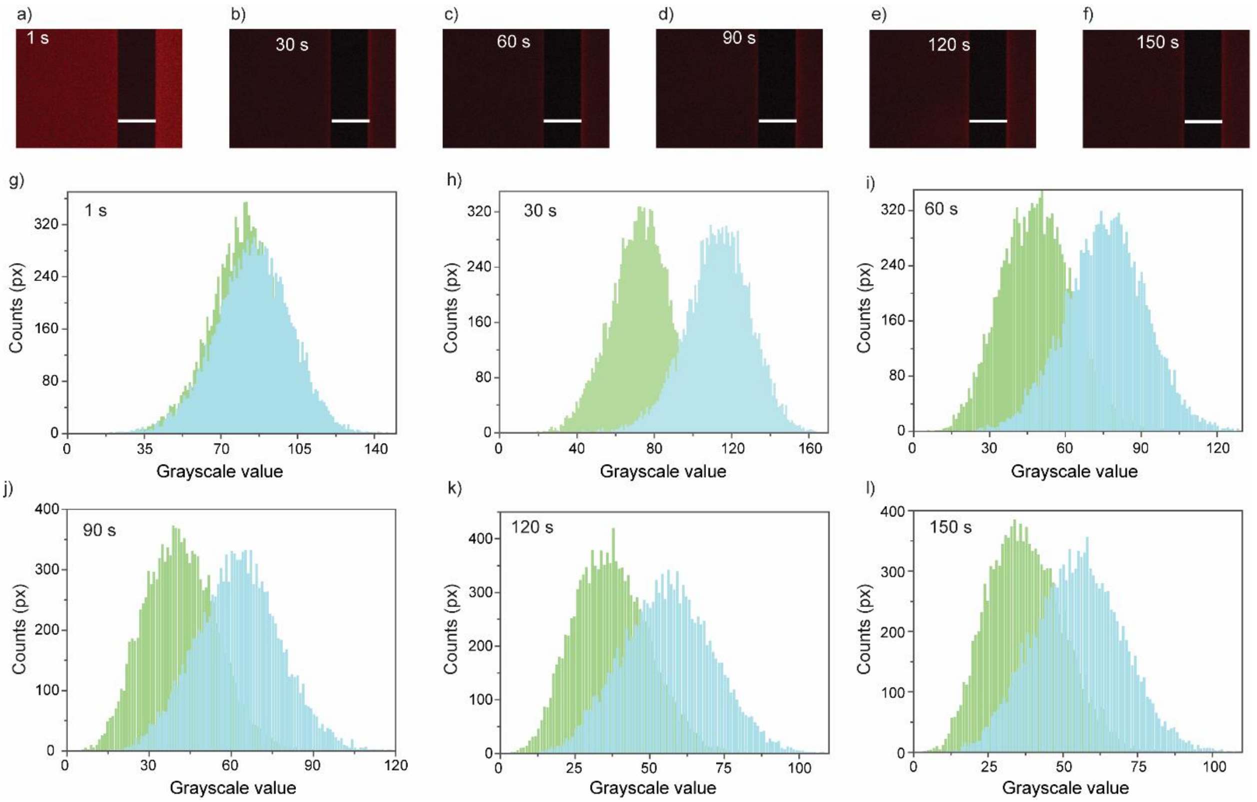

Figure S19. Representative ECL micrographs and region of interest ECL intensity histograms. Representative ECL micrographs and region-of-interest ECL intensity histograms. Representative ECL microscopy data (a–f), and their descriptive (histograms) data analysis (g–l) used to compute the ECL augmentation versus luminophore concentration data as shown in Figure 3e of the main text. The ECL solution contained 3 mM $Ru(bpy)_3]^{2+}$ and 18 mM oxalate in aqueous H_2SO_4 (0.1 M, corrected to pH 6). ECL micrographs were collected at an ITO electrode (50 × 50 × 1 mm, with a sheet resistance of 7–10 ohms per square) using a Nikon inverted microscope (2× magnification) during potentiostatic (chronoamperometry) experiments with the working electrode bias set to 1.4 V (vs Ag|AgCl, 3.4 M KCl). The duration of the experiments was generally equal or greater than 150 s. A platinum wire (0.5 mm diameter wire, 99.99+%, Goodfellow Cambridge Limited) served as the counter electrode. The red channel histograms, built from the ECL data (micrographs shown in a–f) with the open-source image processing software Fiji, refer to specific regions of interest (ROI). The ROIs were in the shape of vertical rectangles, as shown in Figure 3a of the main text (10 × 1200 px dimensions). The scale bars in figure indicate 400 µm. The blue color data correspond to ECL intensity sampled in ROIs near the insulating object (dark vertical lines in the ECL micrographs), while green color data corresponds to the ECl emission sampled away from the insulator (see Figure 3a). The intensity data shown in Figure 3e are taken as the area under the histogram, obtained by integrating the counts over the relevant range. The electrolysis time is shown in each panel (both in the micrographs as well as in the histograms).

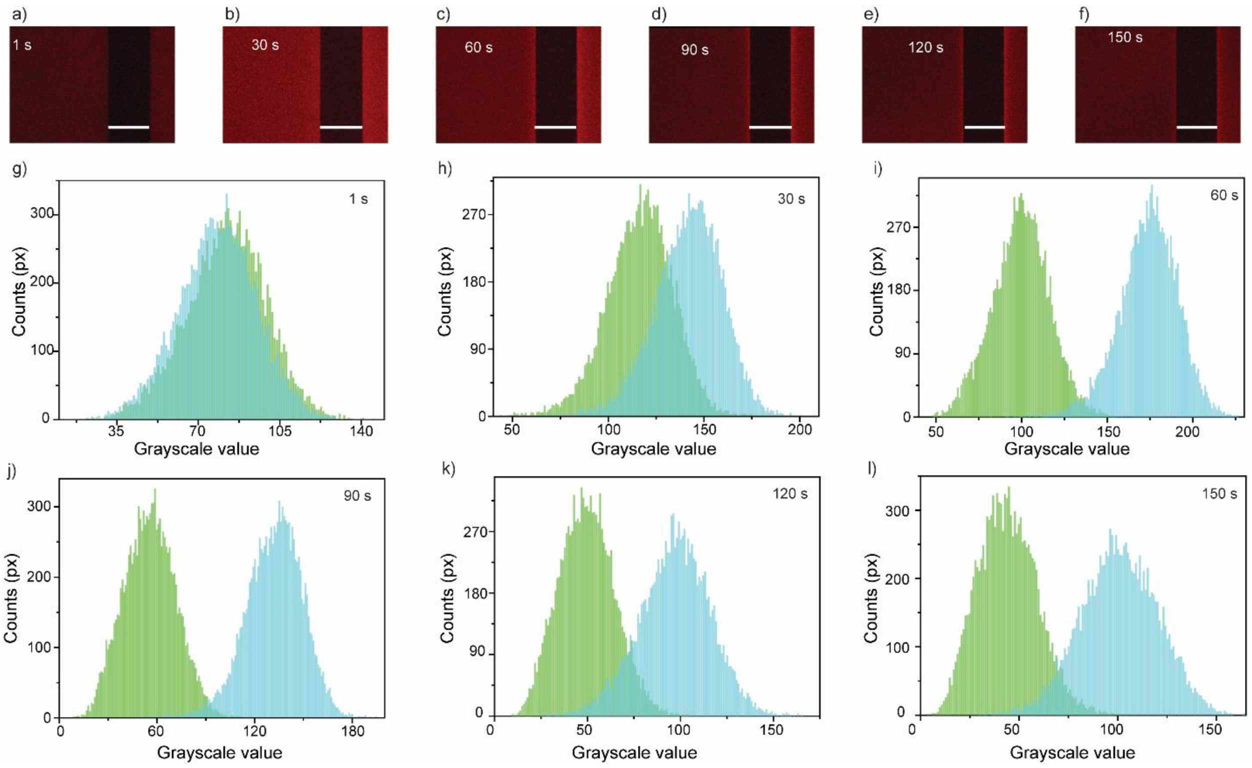

Figure S20. Representative ECL micrographs and region of interest ECL intensity histograms. Representative ECL micrographs and region-of-interest ECL intensity histograms. Representative ECL microscopy data (a–f), and their descriptive (histograms) data analysis (g–l) used to compute the ECL augmentation versus luminophore concentration data as shown in Figure 3e of the main text. The ECL solution contained 4 mM $Ru(bpy)_3]^{2+}$ and 24 mM oxalate in aqueous H_2SO_4 (0.1 M, corrected to pH 6). ECL micrographs were collected at an ITO electrode (50 × 50 × 1 mm, with a sheet resistance of 7–10 ohms per square) using a Nikon inverted microscope (2× magnification) during potentiostatic (chronoamperometry) experiments with the working electrode bias set to 1.4 V (vs Ag|AgCl, 3.4 M KCl). The duration of the experiments was generally equal or greater than 150 s. A platinum wire (0.5 mm diameter wire, 99.99+%, Goodfellow Cambridge Limited) served as the counter electrode. The red channel histograms, built from the ECL data (micrographs shown in a–f) with the open-source image processing software Fiji, refer to specific regions of interest (ROI). The ROIs were in the shape of vertical rectangles, as shown in Figure 3a of the main text (10 × 1200 px dimensions). The scale bars in the figure indicate 400 µm. The blue color data correspond to ECL intensity sampled in ROIs near the insulating object (dark vertical lines in the ECL micrographs), while the green color data corresponds to the ECl emission sampled away from the insulator (see Figure 3a). The intensity data shown in Figure 3e are taken as the area under the histogram, obtained by integrating the counts over the relevant range. The electrolysis time is shown in each panel (both in the micrographs as well as in the histograms).

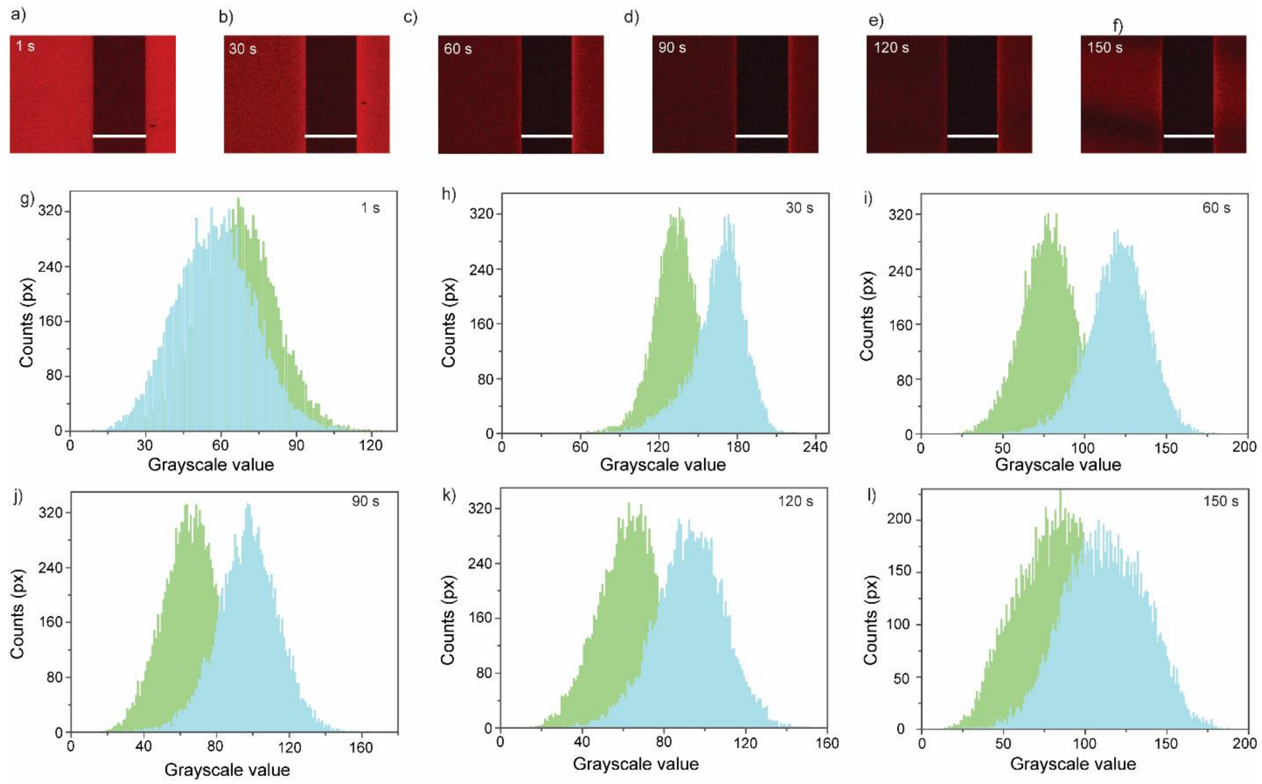

Figure S21. Representative ECL micrographs and region of interest ECL intensity histograms. Representative ECL micrographs and region-of-interest ECL intensity histograms. Representative ECL microscopy data (a–f), and their descriptive (histograms) data analysis (g–l) used to compute the ECL augmentation versus luminophore concentration data as shown in Figure 3e of the main text. The ECL solution contained 4.5 mM $Ru(bpy)_3]^{2+}$ and 27 mM oxalate in aqueous H_2SO_4 (0.1 M, corrected to pH 6). ECL micrographs were collected at an ITO electrode (50 × 50 × 1 mm, with a sheet resistance of 7–10 ohms per square) using a Nikon inverted microscope (2× magnification) during potentiostatic (chronoamperometry) experiments with the working electrode bias set to 1.4 V (vs Ag|AgCl, 3.4 M KCl). The duration of the experiments was generally equal or greater than 150 s. A platinum wire (0.5 mm diameter wire, 99.99+%, Goodfellow Cambridge Limited) served as the counter electrode. The red channel histograms, built from the ECL data (micrographs shown in a–f) with the open-source image processing software Fiji, refer to specific regions of interest (ROI). The ROIs were in the shape of vertical rectangles, as shown in Figure 3a of the main text (10 × 1200 px dimensions). The scale bars in figure indicate 400 µm. The blue color data correspond to ECL intensity sampled in ROIs near the insulating object (dark vertical lines in the ECL micrographs), while green color data corresponds to the ECl emission sampled away from the insulator (see Figure 3a). The intensity data shown in Figure 3e are taken as the area under the histogram, obtained by integrating the counts over the relevant range. The electrolysis time is shown in each panel (both in the micrographs as well as in the histograms).

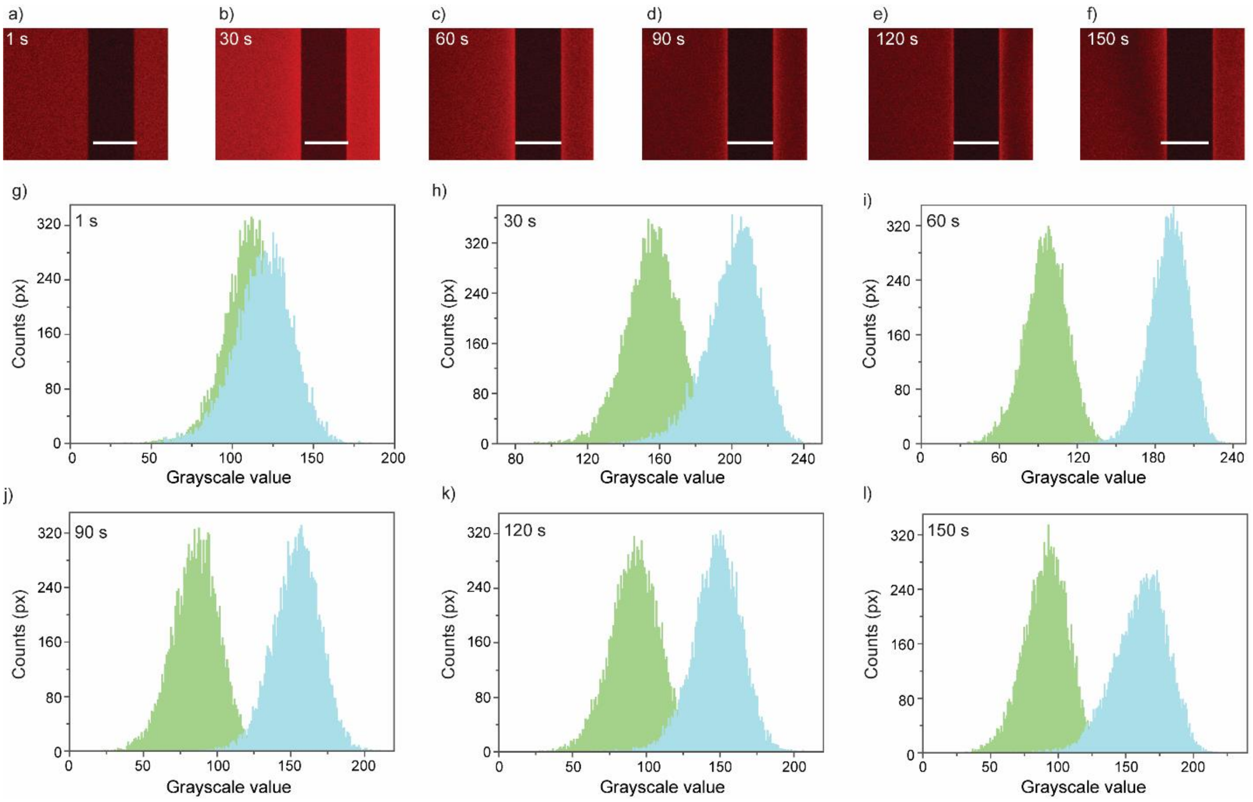

Figure S22. Representative ECL micrographs and region of interest ECL intensity histograms. Representative ECL micrographs and region-of-interest ECL intensity histograms. Representative ECL microscopy data (a–f), and their descriptive (histograms) data analysis (g–l) used to compute the ECL augmentation versus luminophore concentration data as shown in Figure 3e of the main text. The ECL solution contained 5 mM $Ru(bpy)_3]^{2+}$ and 30 mM oxalate in aqueous H_2SO_4 (0.1 M, corrected to pH 6). ECL micrographs were collected at an ITO electrode (50 × 50 × 1 mm, with a sheet resistance of 7–10 ohms per square) using a Nikon inverted microscope (2× magnification) during potentiostatic (chronoamperometry) experiments with the working electrode bias set to 1.4 V (vs Ag|AgCl, 3.4 M KCl). The duration of the experiments was generally equal or greater than 150 s. A platinum wire (0.5 mm diameter wire, 99.99+%, Goodfellow Cambridge Limited) served as the counter electrode. The red channel histograms, built from the ECL data (micrographs shown in a–f) with the open-source image processing software Fiji, refer to specific regions of interest (ROI). The ROIs were in the shape of vertical rectangles, as shown in Figure 3a of the main text (10 × 1200 px dimensions). The scale bars in figure indicate 400 µm. The blue color data correspond to ECL intensity sampled in ROIs near the insulating object (dark vertical lines in the ECL micrographs), while green color data corresponds to the ECl emission sampled away from the insulator (see Figure 3a). The intensity data shown in Figure 3e are taken as the area under the histogram, obtained by integrating the counts over the relevant range. The electrolysis time is shown in each panel (both in the micrographs as well as in the histograms).

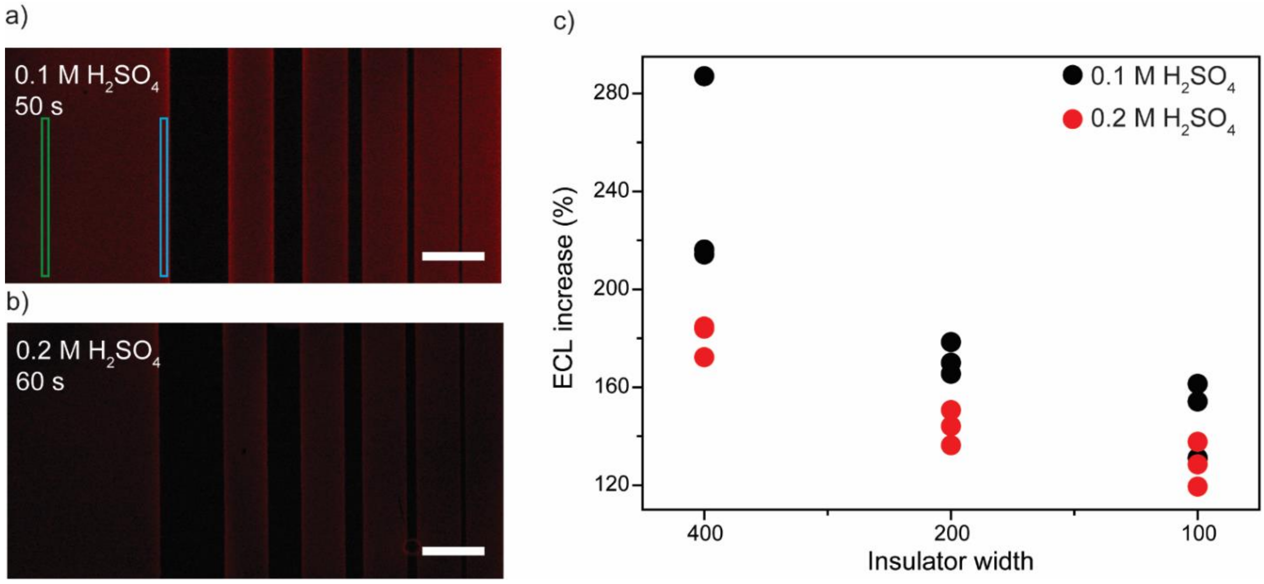

Figure S23. Near-insulator ECl augmentation as a function of electrolytic support. (a,b) ECL micrographs (2×, inverted microscope; scale bars: 400 µm) acquired at ITO electrodes partially masked with NLOF 2035 line-shaped patterns (5 µm thick and of width ranging from 25 to 400 µm). The electrode bias is +1.4 V (vs Ag|AgCl, 3.4 M KCl) and the ECL solution is 4 mM $[Ru(bpy)_3]^{2+}$ and 24 mM oxalate, in either (a) aqueous 0.1 M H_2SO_4, or (b) aqueous 0.2 M H_2SO_4. The solution pH was adjusted to 6 through the dropwise addition of aqueous 10 M NaOH. ECL micrographs in (a,b) were obtained at electrolysis times ranging between 50 and 60 s. (c) Plot of the maximum percentage increase in near–insulator ECL intensity relative to ECL at clear electrode regions (e.g., blue (near insulator) vs. green (clean) regions of interest marked in (a), each measuring 10 × 1200 px). The entries in (c) are the weighted summations of ECL microscopy grayscale intensity histograms for the above mentioned regions of interest.

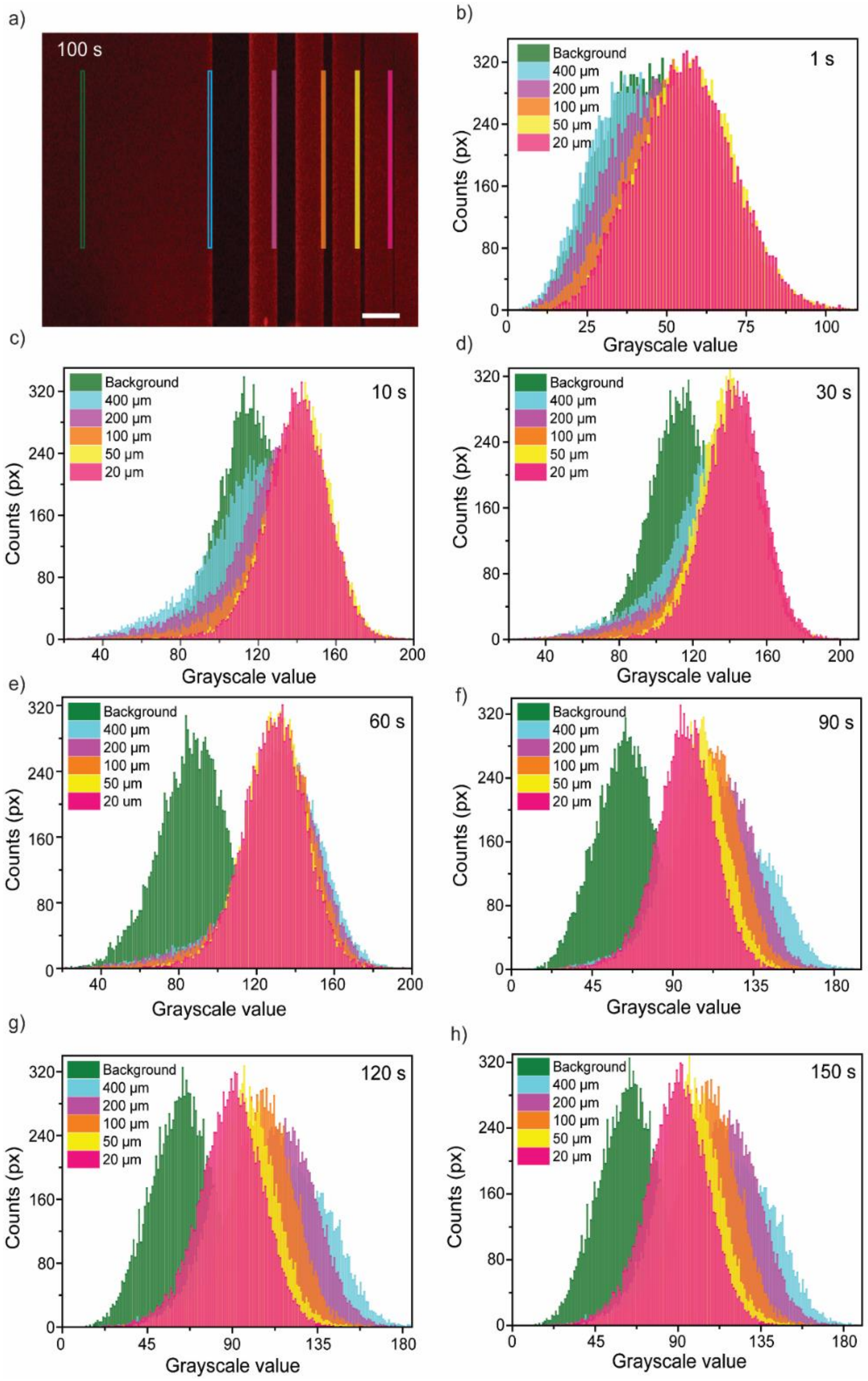

Figure S24. ECL intensity histograms indicating non-Cottrell decay of ECL rates (emission intensity) near blocked electrode regions. (a) ECL micrograph (2×, inverted microscope, 1100 × 1500 px) for the anodic electrolysis of $[Ru(bpy)_3]^{2+}$ (4 mM) and oxalate (24 mM) in aqueous H_2SO_4 (0.1 M), adjusted to pH 6 using 10 M NaOH captured 100 s after applying a bias voltage of +1.4 V (vs Ag|AgCl, 3.4 M KCl). The scale bar is 400 µm. The green color box corresponds to the background area (region of interest, ROI) while the blue, purple, orange, yellow, and pink boxes define the near-insulator the ROIs for blocks of 400, 200, 100, 50, and 20 µm width, respectively. (b–f) Red channel histograms (Fiji) built from ECL data sampled at the ROIs marked in (a) for different electrolysis times (times indicated in each panel). The ECL histograms for each width were obtained by averaging the counts from both sides of the pattern, and the ROI on the right of the pattern is not shown in (a) for clarity purpose.

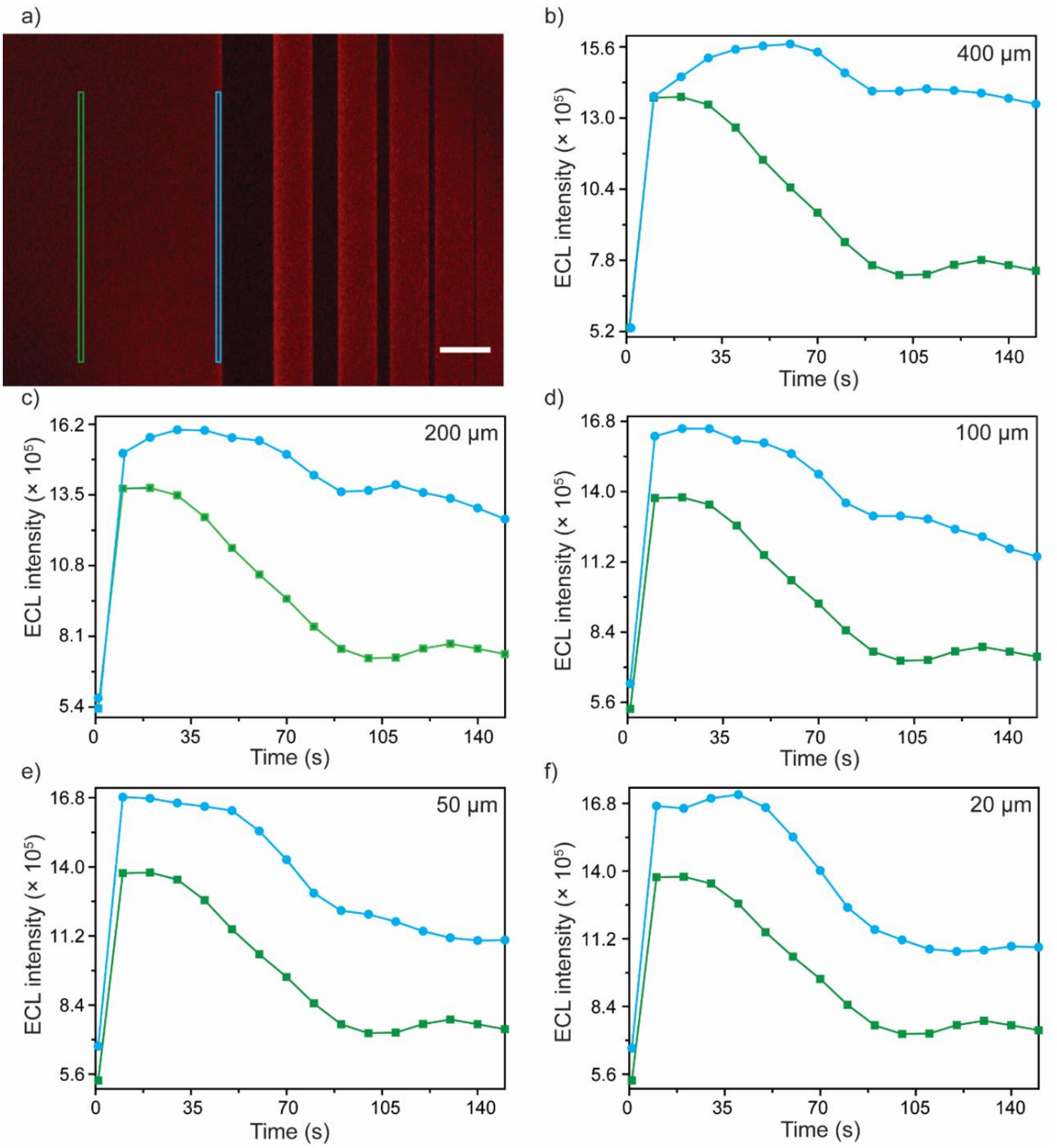

Figure S25. Non-Cottrell decay of the ECL rate (emission intensity) near hydrophobe patterns. (a) ECL micrograph captured 100 s after applying a bias voltage of +1.4 V (vs Ag|AgCl, 3.4 M KCl). The ECL solution consisted of $[Ru(bpy)_3]^{2+}$ (4 mM) and oxalate (24 mM) in aqueous H_2SO_4 (0.1 M), adjusted to pH 6 using 10 M NaOH. The scale bar is 400 µm and the hydrophobe thickness is 3µm. The green and blue boxes (ROI), with the dimensions of 10 × 1200 px, represent the areas corresponding to the background and the near-hydrophobe ROIs, respectively. (b–f) ECL intensity vs time plots for different blocks (line-shaped) with widths of 400, 200, 100, 50, and 20 µm (background, green; near-insulator, blue). For each width, the ECL intensity is the average of left- and right-hand sides ROIs (only left ROIs shown in (a) for clarity).

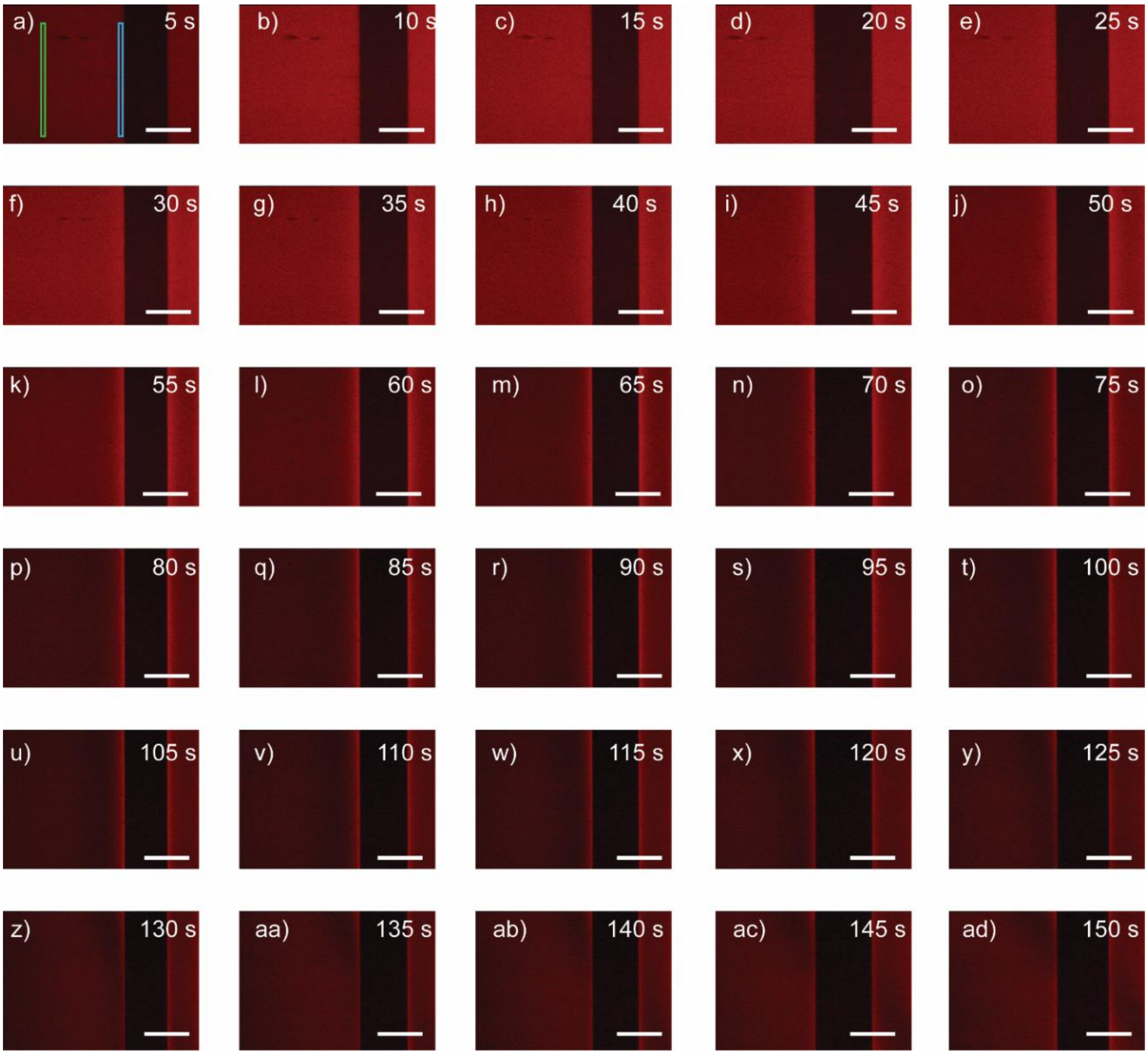

Figure S26. Representative time-stamped ECL micrographs. Evolution of the ECL over a 150-second electrolysis (5-second time intervals between frames). Micrographs collected through ECL microscopy (Nikon, 2×, inverted microscope). The blue and green boxes (10 × 1200 px) represent the regions of interest (ROI) considered for the comparison of near versus away from insulator rates, respectively. The electrolysis was done in a solution containing $[Ru(bpy)_3]^{2+}$ (4 mM) and oxalate (24 mM) in aqueous H_2SO_4 (0.1 M), adjusted to pH 6. The images highlight spatial variations in ECL intensity near and away from ITO electrodes partially fouled with hydrophobic regions (NLOF 2035 photoresist line-shaped patterns). ECL micrographs were captured after applying a bias voltage of +1.4 V (vs Ag|AgCl, 3.4 M KCl). The scale bar is 400 µm.

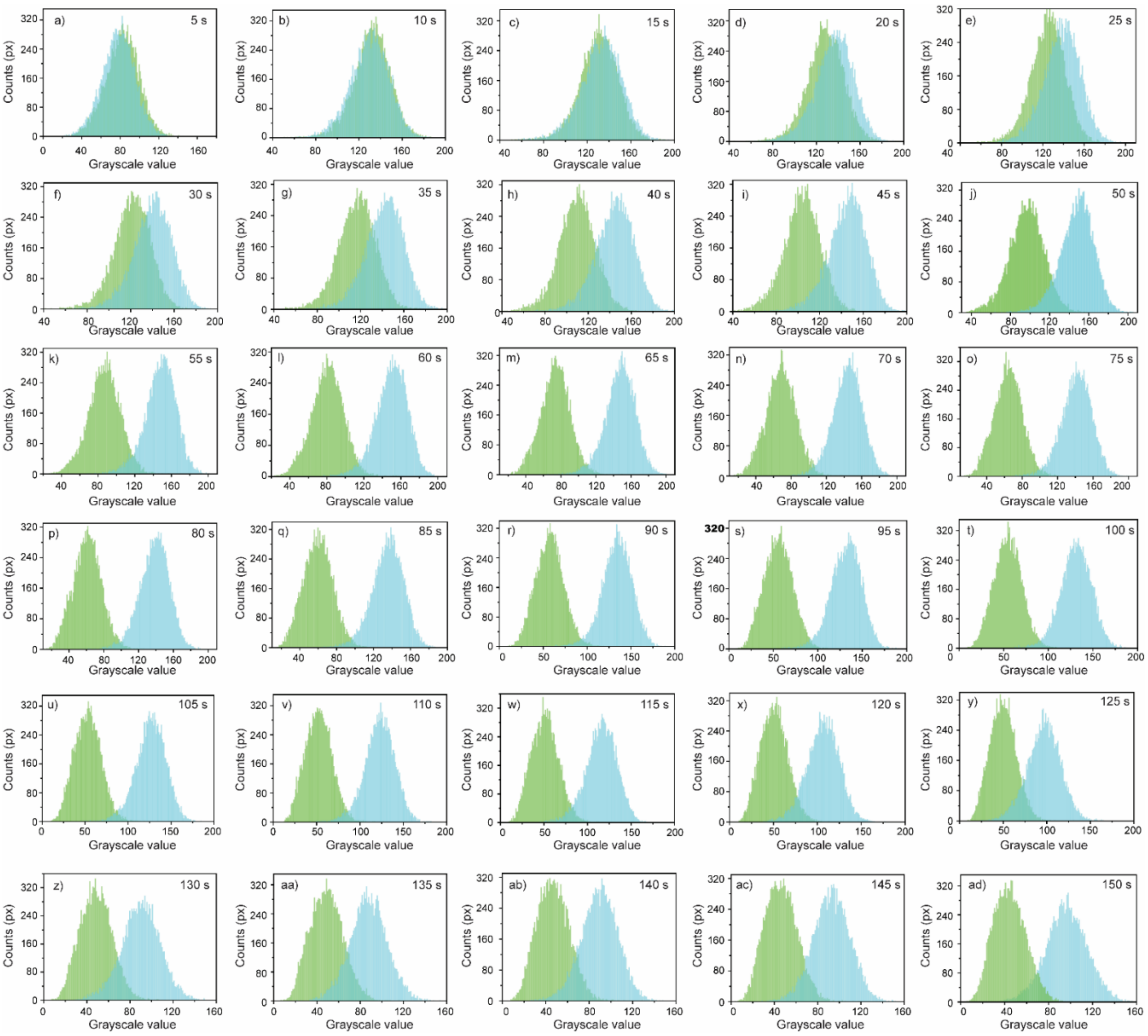

Figure S27. ECL intensity histograms for electrode regions either near or away from the fouling object. ECL intensity histograms corresponding to the data in Figure S26 (near-insulator ECL, blue; away from the insulator, green).

Table S2. Electrolysis time required for the near–insulator ECL to reach a maximum intensity over the background. The ECL system consisted with the luminophore $Ru(bpy)_3^{2+}$, and the co–reactant, $C_2O_4^{2-}$ at pH 6 (adjusted by adding 10 M NaOH). Preparation of the ECL solution was mentioned in the experimental section.

The concentration of the ECL solution and the thickness of the photoresist	Time (s)					
	E 1	E 2	E 3	E 4	E 5	E 6
1 mM $Ru(bpy)_3^{2+}$: 6 mM $C_2O_4^{2-}$ NLOF 2035 Thickness 3 µm	80	80	60	80	90	90
	80	70	70	70	70	70
	60	80	80	100	80	90
2 mM $Ru(bpy)_3^{2+}$: 12 mM $C_2O_4^{2-}$ NLOF 2035 Thickness 3 µm	80	80	80	100	80	90
	70	70	70	100	100	80
	100	100	100	100	100	100
3 mM $Ru(bpy)_3^{2+}$: 18 mM $C_2O_4^{2-}$ NLOF 2035 Thickness 3 µm	70	70	80	80	100	100
	40	80	60	70	70	70
	60	60	70	70	80	100
4 mM $Ru(bpy)_3^{2+}$: 24 mM $C_2O_4^{2-}$ NLOF 2035 Thickness 3 µm	70	100	70	70	90	90
	100	90	80	70	80	80
	100	80	80	80	100	100
4 mM $Ru(bpy)_3^{2+}$: 24 mM $C_2O_4^{2-}$ NLOF 2035 Thickness 4 µm	20	90	20	20	20	20
	60	60	60	60	80	80
	40	90	80	30	40	40
4 mM $Ru(bpy)_3^{2+}$: 24 mM $C_2O_4^{2-}$ NLOF 2035 Thickness 5 µm	100	70	100	70	100	100
	60	80	80	100	80	90
	70	80	80	80	90	90
4 mM $Ru(bpy)_3^{2+}$: 24 mM $C_2O_4^{2-}$ NLOF 2035 Thickness 6 µm	100	100	100	100	90	100
	80	80	80	80	90	90
	60	70	60	60	60	60
4.5 mM $Ru(bpy)_3^{2+}$: 27 mM $C_2O_4^{2-}$ NLOF 2035 Thickness 3 µm	80	70	70	70	80	80
	60	60	60	60	70	80
	60	60	70	60	80	80
5 mM $Ru(bpy)_3^{2+}$: 30 mM $C_2O_4^{2-}$ NLOF 2035 Thickness 3 µm	90	90	80	80	90	90
	70	90	80	70	70	70
	90	90	90	90	60	70

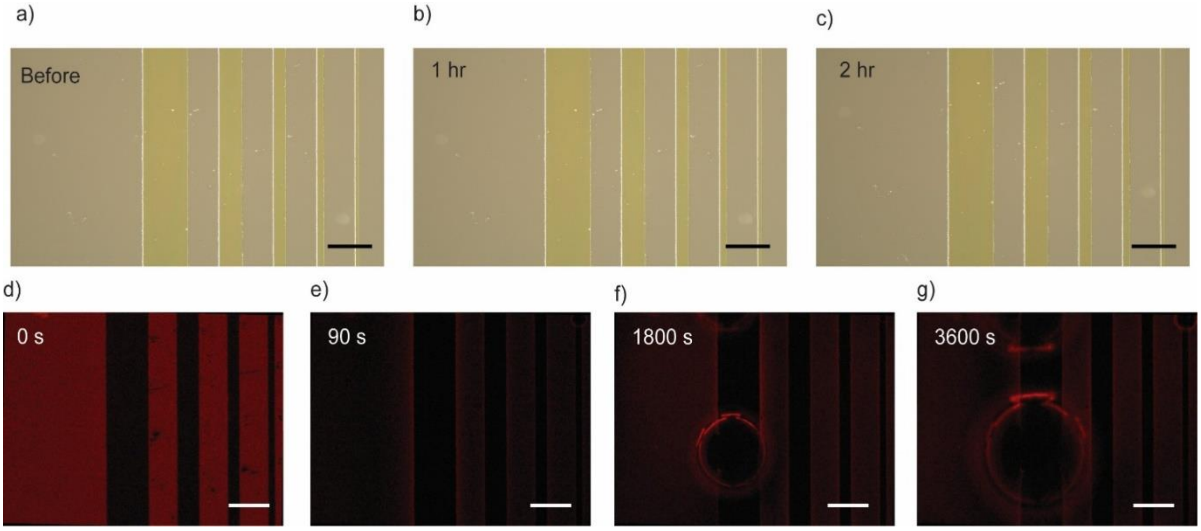

Figure S28. Stability assessment of lithographically patterned ITO electrodes towards anodic biasing (+1.4 V vs Ag|AgCl, 3.4 M KCl). Electrodes were decorated with line-shaped features of NLOF 2035 (variable widths, 6 µm thick). Bright field microscopy images of the patterned electrode (2×, inverted microscope), (a) before, and (b) after a 1-hour long ECL experiment, followed by extensive water rinsing and drying under a stream of N_2 gas. (c) Bright field image after an additional 1-hour long ECL experiment, again followed by electrode rinsing and drying. (d–g) Time-stamped ECL microscopy images (2×, inverted microscope, 1100 × 1500 px) for the anodic electrolysis of $[Ru(bpy)_3]^{2+}$ (4 mM) and oxalate (24 mM) in aqueous H_2SO_4 (0.1 M, corrected to pH 6) sampled after (d) 0 s, (e) 90 s, (f) 1800 s, and (g) 3600 s of continuous electrode biasing. Scale bars are 400 µm. Despite prolonged ECL operation and visible gas bubble formation, the hydrophobic layer remained structurally intact, indicating strong stability under anodic regimes.

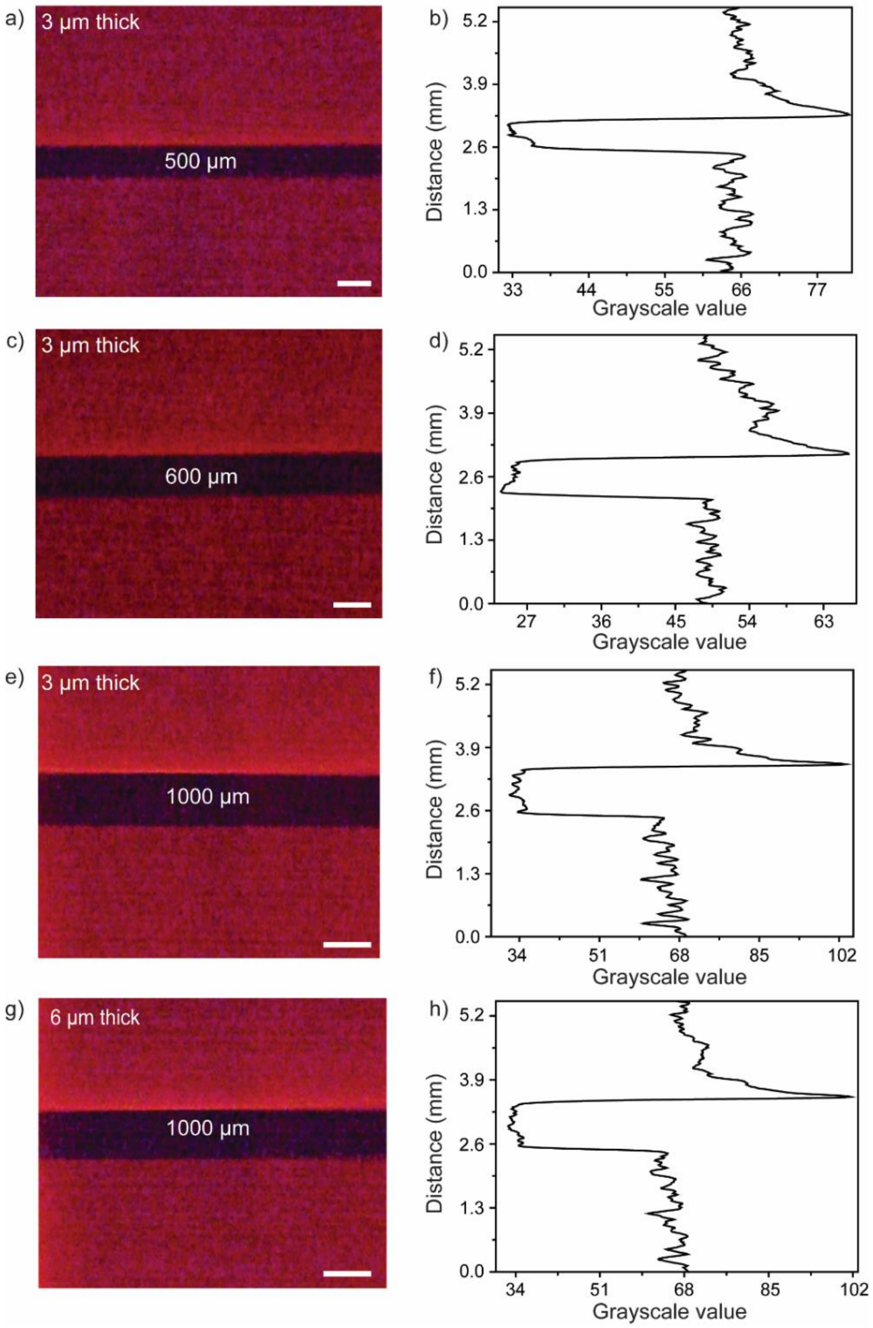

Figure S29. Buoyancy forces and density gradient-driven convection: effect of the insulating feature thickness and width. The ECL solution was prepared using the same composition of the luminophore and the co-reactant as described in the experimental section. ECL micrographs (left column), and their corresponding plot profiles (right panel), were acquired at an electrolysis time ranging from 60 to 100 s. The insulating feature was a NLOF 3035 line-shaped pattern (width and thickness indicated in the figure). Scale bars are (a) 500 µm, (c) 600 µm, (e) 1000 µm, (g) 1000 µm. ECL micrographs were captured by applying a bias voltage of +1.4 V (vs Ag|AgCl, 3.4 M KCl). The ITO electrodes served as a working electrode and a Pt coil as the counter electrode.

Table S3. Zeta potential values for the photoresists NLOF 2035 and SU-8 2002.

	NLOF 2035 (mV)	SU–8 2002 (mV)
	-28.7	-9.7
	-25.5	-9.2
	-21.2	-14.0
	-24.1	-9.2
	-26.5	-5.0
	-23.4	-18.5
average	-24.9	-10.9
SD	2.4	4.3

Table S4. Contact Tabled water contact angle data for NLOF 2035 and SU–8 2002 films.

	NLOF (˚)	SU (˚)
	86.1	91.0
	84.4	106.6
	86.1	89.9
	86.0	93.3
	86.2	92.5
	87.6	94.2
	87.0	93.5
	85.3	96.9
	80.2	93.2
	86.0	98.5
	83.6	96.2
	79.3	96.5
	78.8	96.0
	83.5	98.6
	84.0	98.4
	85.4	105.5
	84.4	100.2
	83.9	98.4
	84.0	104.0
	86.2	99.3
average	84.4	97.1
SD	2.4	4.4

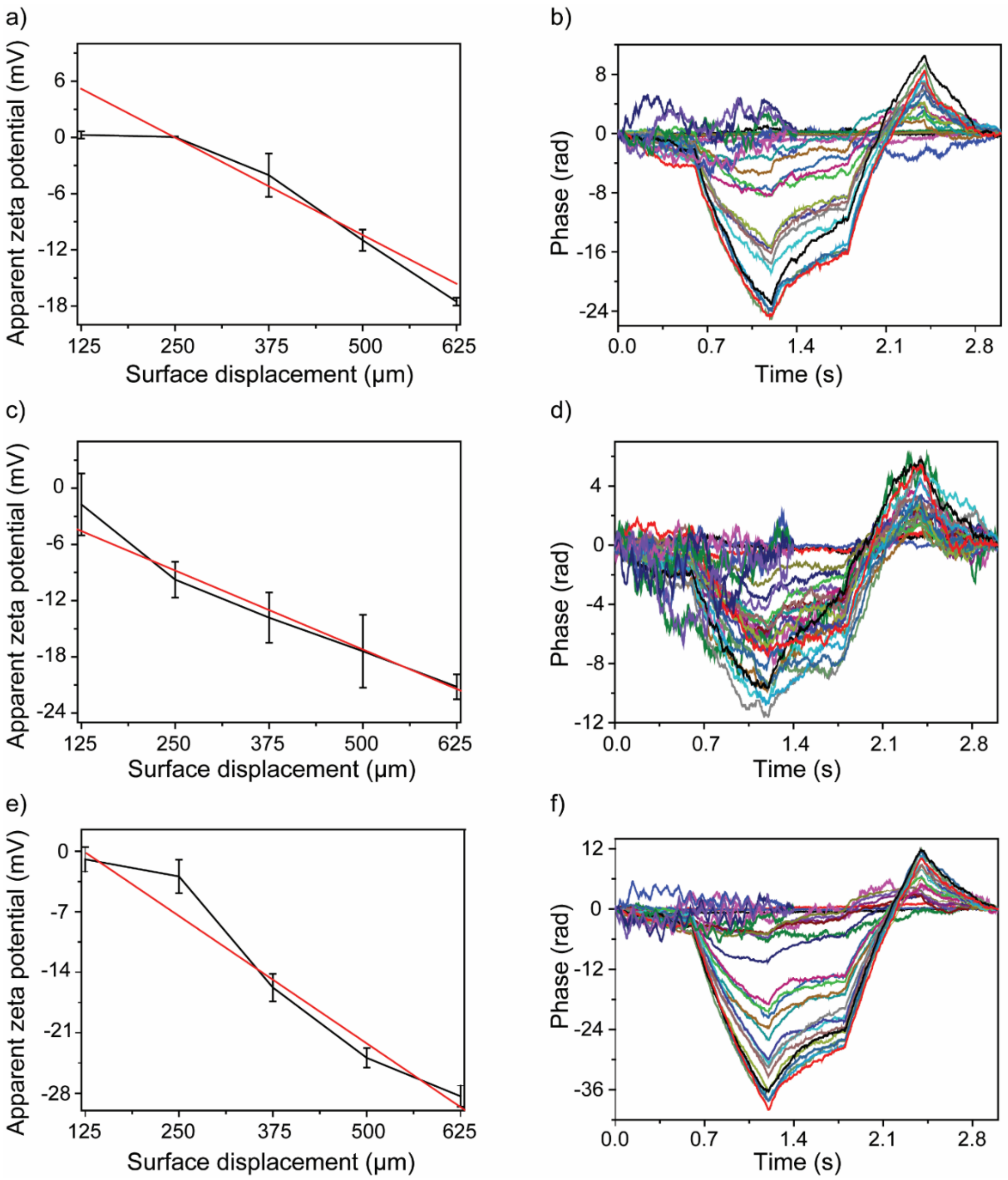

Figure S30. Surface zeta potential data for films of SU–8 2002. The ECL solution was analogous to that described in the experimental section (main text) except for a 10× dilution factor (0.4 mM, $[Ru(bpy)_3]^{2+}$; 2.4 mM oxalate). The photoresist (SU–8 2002) was spin-coated on a PEEK sample holder to achieve a with 3 µm film thickness. Then spin-coated samples were then left to dry overnight prior to analysis. (a, c, e) Apparent zeta potential versus surface displacement (average of three independent measurements). (b, d, f) Phase plots for the experiments in (a, c, e). The samples were prepared, and the experiments performed at room temperature and pressure. Further details on the surface zeta potential measurements are in the experimental section.

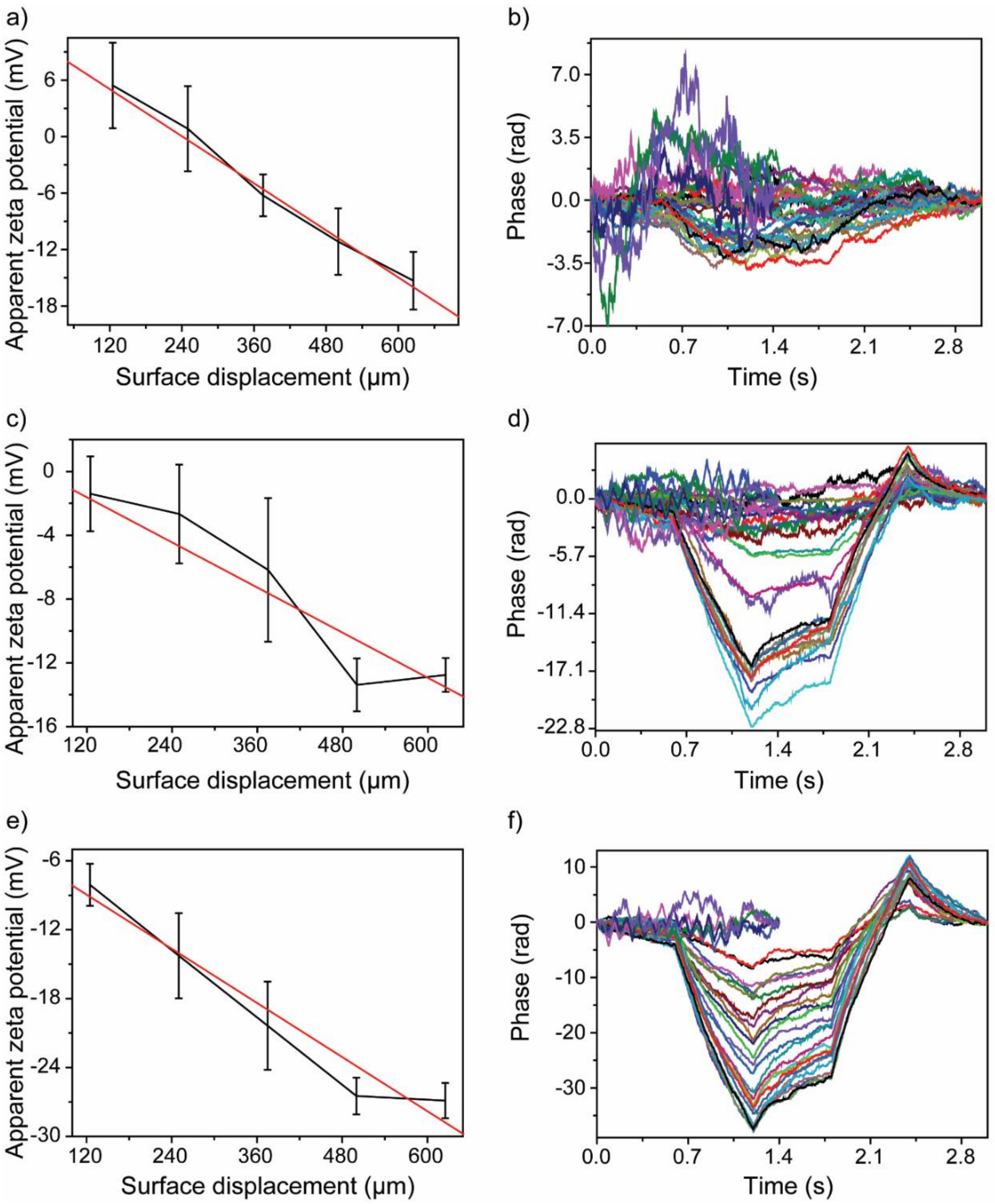

Figure S31. Surface zeta potential data for films of NLOF 2035. The ECL solution was analogous to that described in the experimental section (main text) except for a 10× dilution factor (0.4 mM, $[Ru(bpy)_3]^{2+}$; 2.4 mM oxalate). The photoresist (NLOF 2035) was spin-coated on a PEEK sample holder to achieve a with 3 µm film thickness. Then spin-coated samples were then left to dry overnight prior to analysis. (a, c, e) Apparent zeta potential versus surface displacement (average of three independent measurements). (b, d, f) Phase plots for the experiments in (a, c, e). The samples were prepared, and the experiments performed at room temperature and pressure. Further details on the surface zeta potential measurements are in the experimental section.

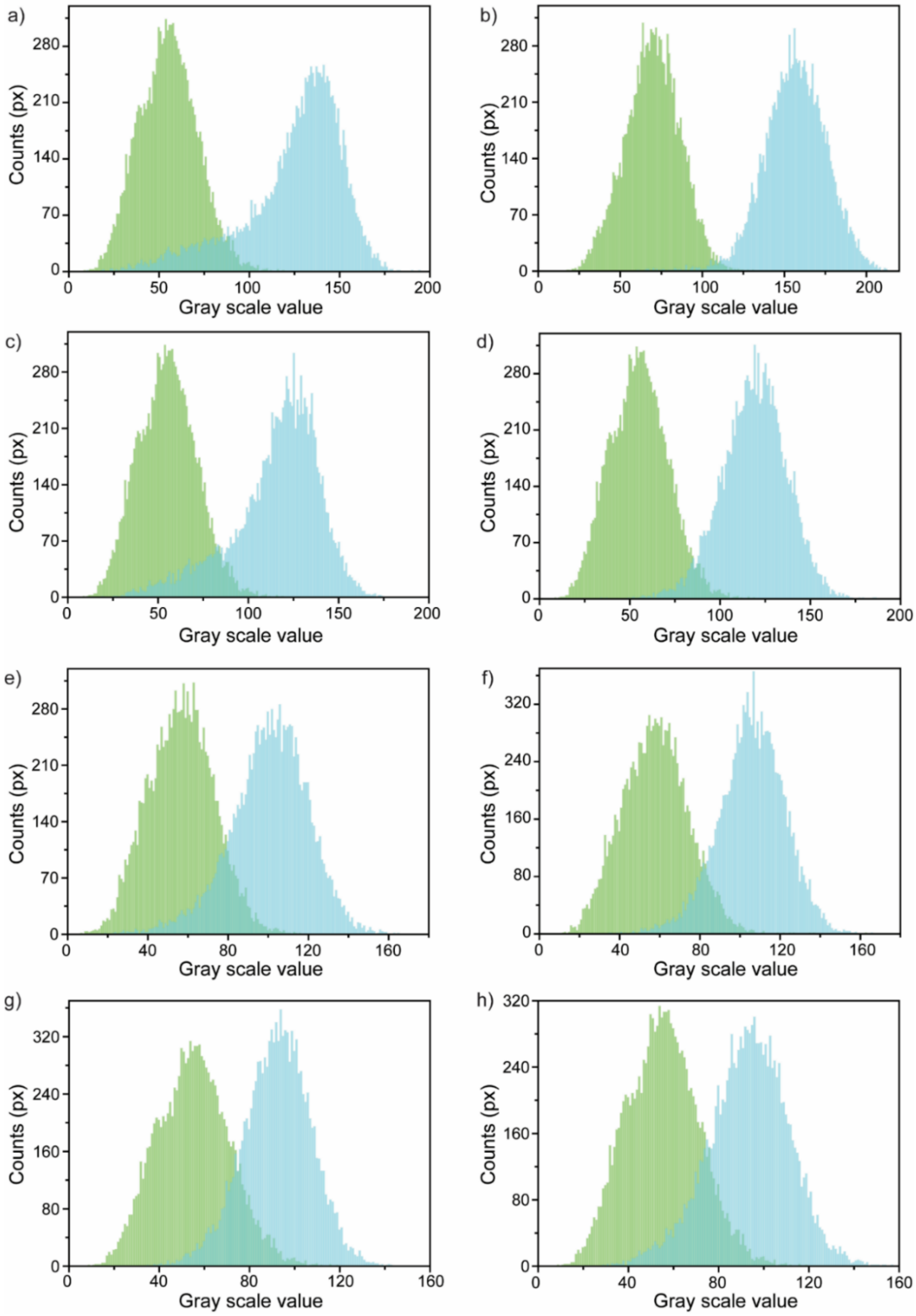

Figure S32. Representative ECL histograms for NLOF 2035 patterns of 3 µm thickness. The ECL solution consisted of 4 mM $[Ru(bpy)_3]^{2+}$ and 24 mM oxalate in 0.1 M H_2SO_4 at pH 6, adjusted using 10 M NaOH. The histograms were derived from the plot in Figure 6c and represent data for different photoresist widths within the range of 60 to 100 s after applying a +1.4 V bias voltage (vs Ag|AgCl, 3.4 M KCl). (a, b) Left and right sides of the considered areas for a 400 µm wide line. (c, d) Left and right sides for a 200 µm wide line. (e, f) Left and right sides for a 100 µm wide line. (g, h) Left and right sides for a 50 µm wide line. The values in Figure 6c correspond to the average of both sides for each width.

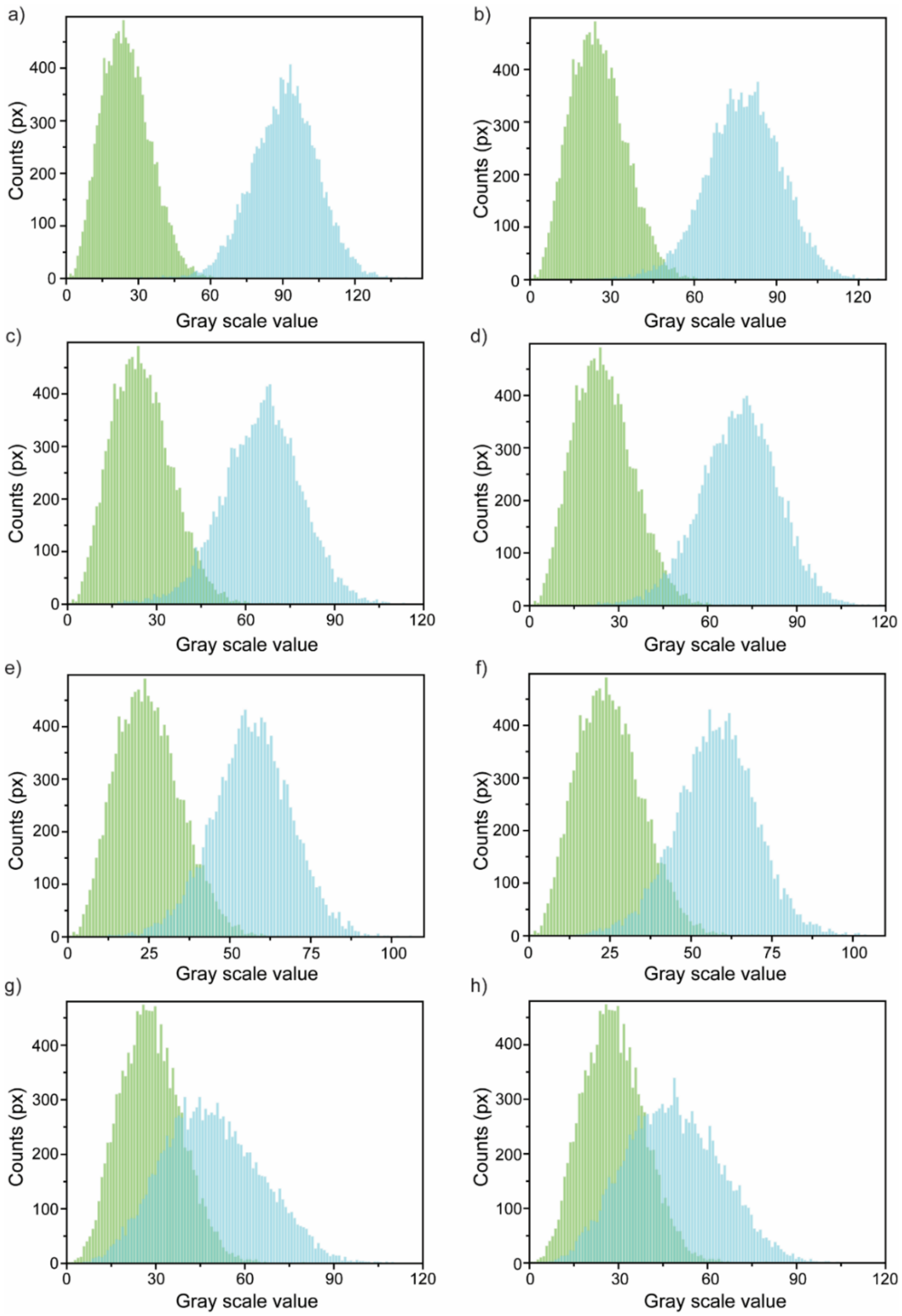

Figure S33. Representative histograms for SU–8 2002 patterns of 3 µm thickness. All the histograms were measured after within the range of 60 to 100 s of applying a +1.4 V bias (vs Ag|AgCl, 3.4 M KCl). The ECL solution consisted of 4 mM $[Ru(bpy)_3]^{2+}$ and 24 mM oxalate in 0.1 M H_2SO_4 at pH 6, adjusted using 10 M NaOH. The six histograms correspond to different photoresist widths: (a, b) left and right sides of the 400 µm wide line, (c, d) left and right sides of the 200 µm wide line, (e, f) left and right sides of the 100 µm wide line, and (g, h) left and right sides of the 50 µm wide line. The values reported in Figure 6c represent the average of the two sides for each width.

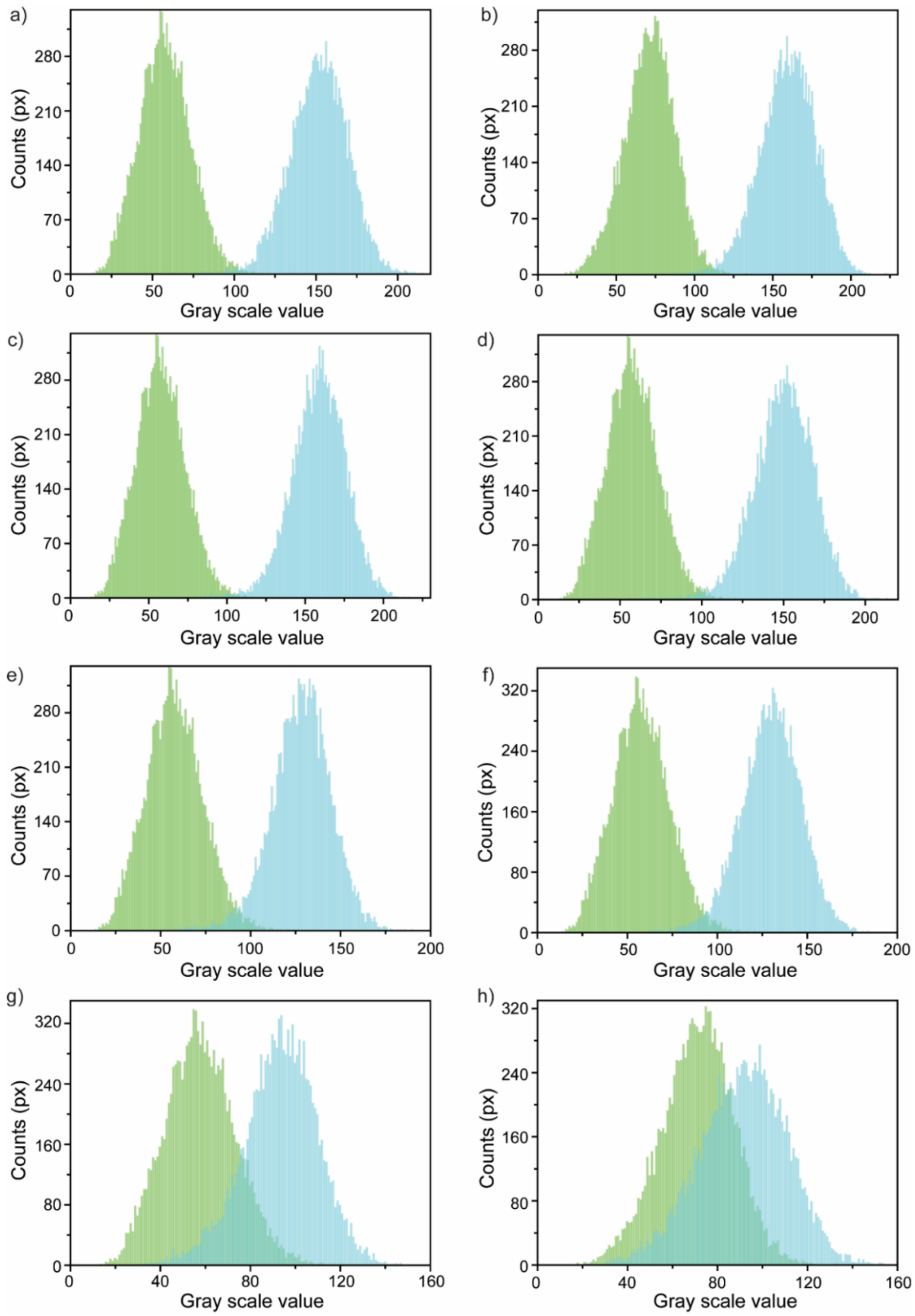

Figure S34. Representative ECL histograms for NLOF 2035 patterns of 4 µm thickness. All histograms correspond to the maximum ECL increase percentage obtained after applying a +1.4 V bias (vs Ag|AgCl, 3.4 M KCl). The maximum values were obtained within the range of 60 to 100 s. The ECL solution consisted of 4 mM $[Ru(bpy)_3]^{2+}$ and 24 mM oxalate in 0.1 M H_2SO_4 at pH 6, adjusted using 10 M NaOH. The six histograms represent different photoresist widths: (a, b) left and right sides of the 400 µm wide line, (c, d) left and right sides of the 200 µm wide line, (e, f) left and right sides of the 100 µm wide line, and (g, h) left and right sides of the 50 µm wide line. The values reported in Figure 6c represent the average of the two sides for each width.

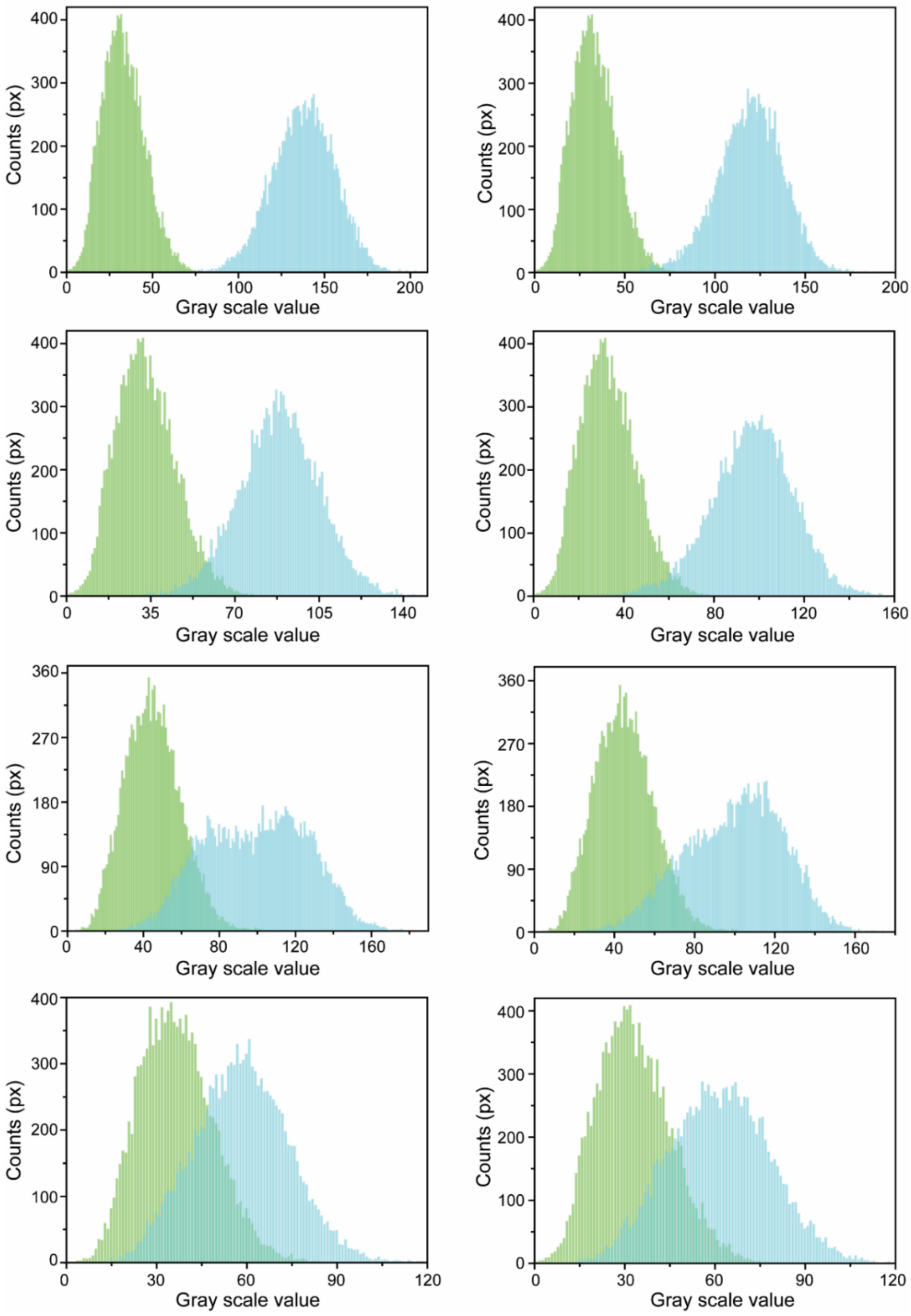

Figure S35. Representative ECL histograms for NLOF 2035 patterns of 6 µm thickness. All histograms correspond to the maximum ECL increase percentage obtained after applying a +1.4 V bias (vs Ag|AgCl, 3.4 M KCl). The maximum values were obtained within the range of 60 to 100 s. The ECL solution consisted of 4 mM $[Ru(bpy)_3]^{2+}$ and 24 mM oxalate in 0.1 M H_2SO_4 at pH 6, adjusted using 10 M NaOH. The six histograms correspond to different photoresist widths: (a, b) left and right sides of the 400 µm wide line, (c, d) left and right sides of the 200 µm wide line, (e, f) left and right sides of the 100 µm wide line, and (g, h) left and right sides of the 50 µm wide line. The values reported in Figure 6c represent the average of the two sides for each width.

Table S5. Capacitance and dielectric constant values for the photoresists NLOF 2035 and SU-8 2002 at room temperature (~22 °C).

Hydrophobe	electrode	Frequency (kHz)	Capacitance (pF)	Dielectric constant	Capacitanace/area (F m^{-2})
NLOF	GC	10	215.9	2.7	7.1E-06
NLOF	GC	10	214.0	2.7	7.1E-06
NLOF	GC	10	214.0	2.7	7.1E-06
NLOF	GC	10	210.3	2.7	7.0E-06
NLOF	GC	100	210.3	2.7	7.0E-06
NLOF	GC	1	224.6	2.9	7.4E-06
NLOF	GC	10	217.9	2.8	7.2E-06
NLOF	GC	10	207.3	2.6	6.9E-06
NLOF	GC	10	258.1	3.3	8.5E-06
NLOF	GC	10	259.2	3.3	8.6E-06
NLOF	GC	1	289.6	3.7	9.6E-06
NLOF	GC	1	289.6	3.7	9.6E-06
Average				2.8	
SU	GC	10	331.2	4.3	1.1E-05
SU	GC	10	332.8	4.4	1.1E-05
SU	GC	10	333.1	4.4	1.1E-05
SU	GC	10	334.1	4.4	1.1E-05
SU	GC	10	334.0	4.4	1.1E-05
SU	GC	10	334.0	4.4	1.1E-05
SU	GC	10	333.7	4.4	1.1E-05
SU	GC	10	327.8	4.3	1.1E-05
SU	GC	10	327.3	4.3	1.1E-05
SU	GC	10	327.3	4.3	1.1E-05
SU	GC	10	327.3	4.3	1.1E-05
SU	GC	10	327.3	4.3	1.1E-05
Average				4.3	

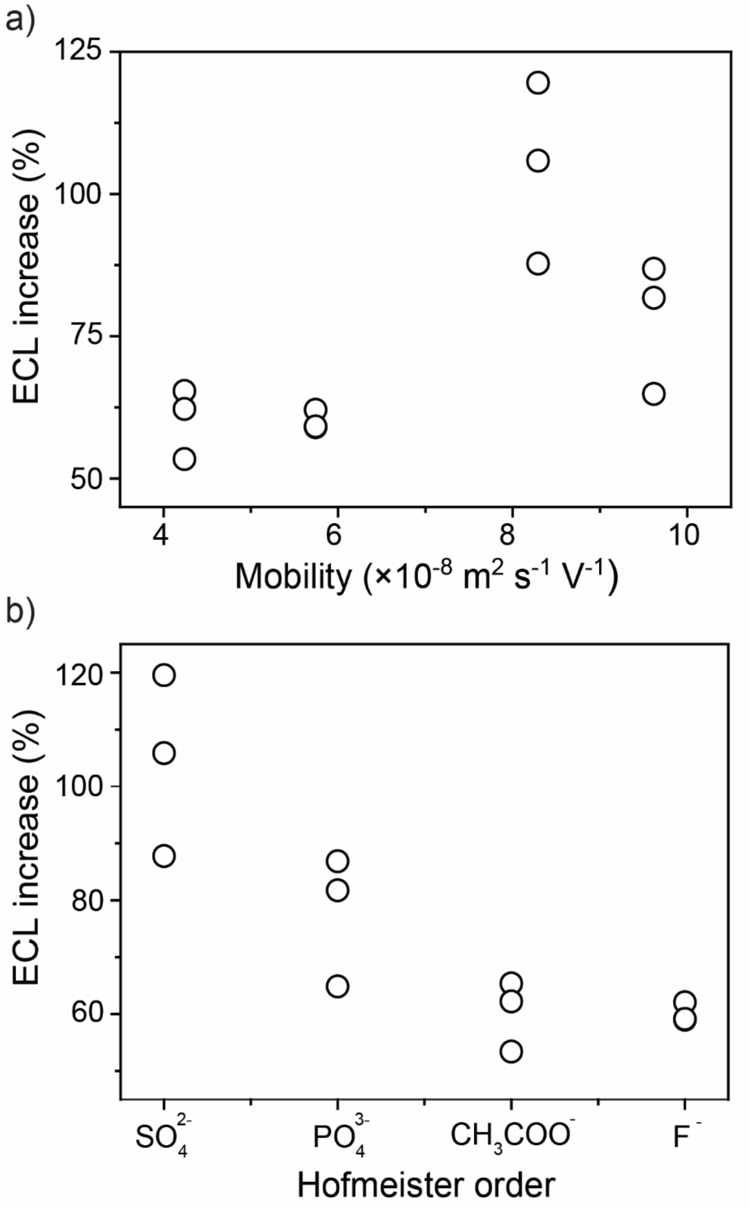

Figure S36. ECL increase percentage variation with different electrolytes. The ECL solution consisted of 4 mM $[Ru(bpy)_3]^{2+}$ and 24 mM oxalate in 0.1 M different salts at pH 6, adjusted using 10 M NaOH. The working electrode was ITO glass, and the reference and counter electrodes were Ag|AgCl in 3.4 M KCl and a Pt coil, respectively. The ECL intensity was calculated from the integrations of histograms relative to ROI of 10 × 1200 px size. (a) The plot of ECL increases percentage change (near the insulator versus away from it) as a function of the mobility of the electrolyte anion (CH_3COO^-,F^-, SO_4^{2-}, PO_4^{3-}). (b) The ECL increase percentage tracks the position of the anion in the Hofmeister series. All experiments were performed at room temperature (22 ± 1 °C).

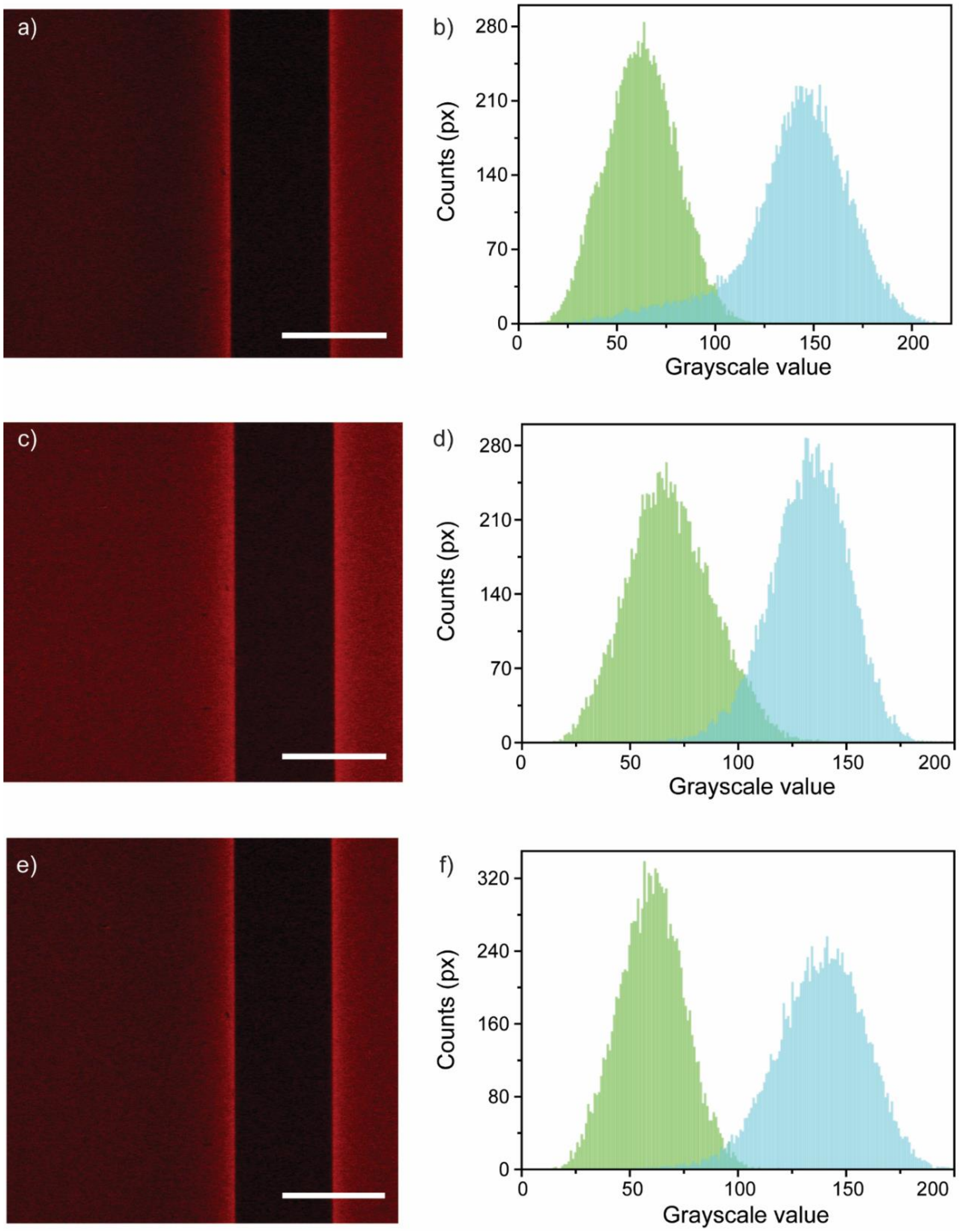

Figure S37. Micrographs and histograms for triplicate ECL microscopy experiments in sulfate-based electrolytes. The ECL solution was 4 mM $[Ru(bpy)_3]^{2+}$ and 24 mM oxalate in 0.1 M H_2SO_4 at pH 6 (adjusted by dropwise addition of 10 M NaOH). ITO glass partially fouled with NLOF 2035 photoresist line-shaped features (thickness 3 µm) is serving as working electrode. The reference and counter electrodes are Ag|AgCl in 3.4 M KCl and a Pt coil, respectively. (a, c, e) ECL micrographs (2×, inverted microscope) of independently prepared and analyzed samples, corresponding to electrolysis times of 90, 120, and 110 s (+1.4 V Ag|AgCl). The scale bars are 400 µm. (b, d, f) ECL histograms for the micrographs in (a), (c), and (e), respectively (green vs. blue, away vs close to the insulator regions of interest, 10 × 1200 px).

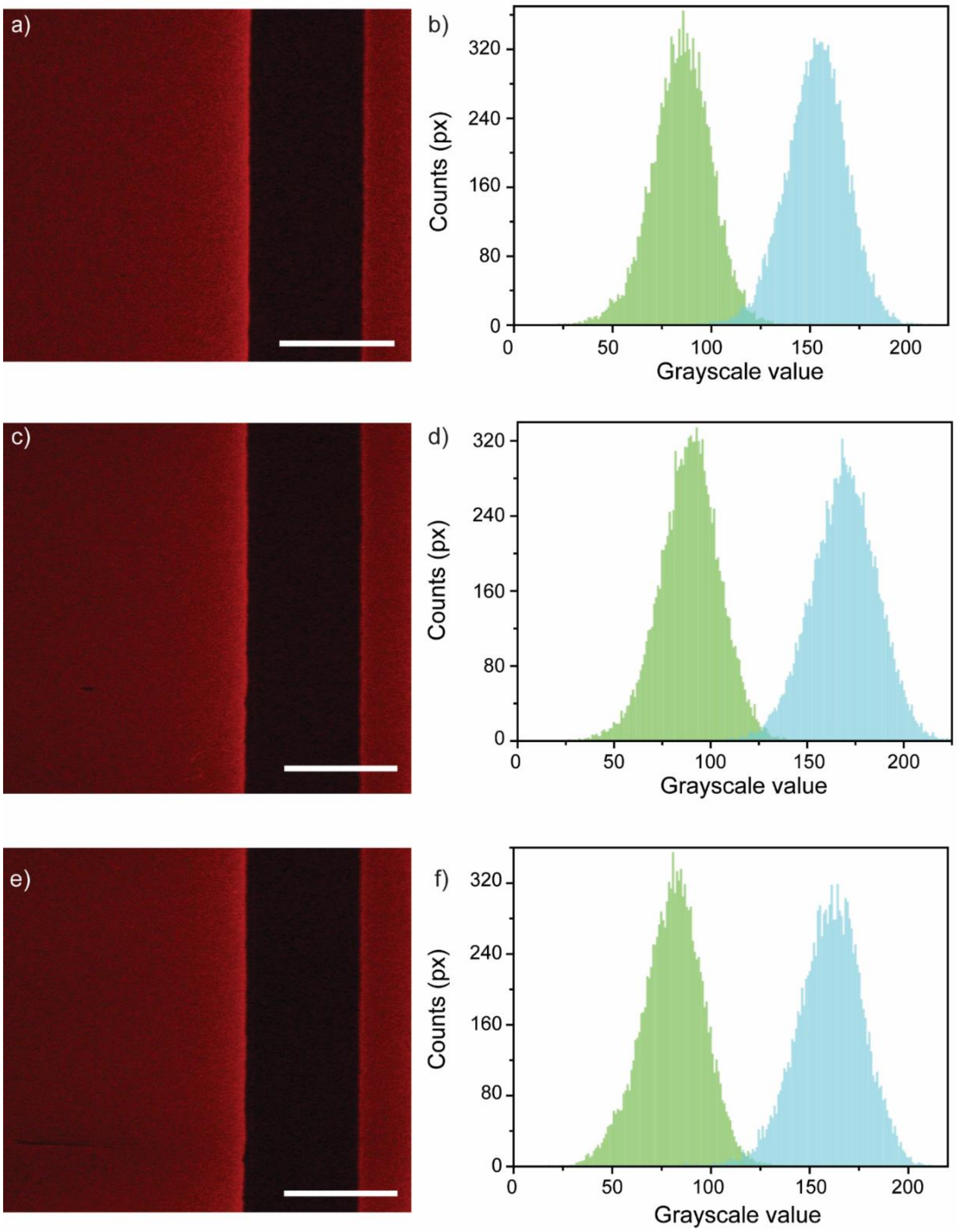

Figure S38. Micrographs and histograms for triplicate ECL microscopy experiments in phosphate-based electrolytes. The ECL solution was 4 mM $[Ru(bpy)_3]^{2+}$ and 24 mM oxalate in 0.1 M H_3PO_4 at pH 6 (adjusted by dropwise addition of 10 M NaOH). ITO glass partially fouled with NLOF 2035 photoresist line-shaped features (thickness 3 µm) is serving as working electrode. The reference and counter electrodes are Ag|AgCl in 3.4 M KCl and a Pt coil, respectively. (a, c, e) ECL micrographs (2×, inverted microscope) of independently prepared and analyzed samples, corresponding to electrolysis times of 80, 60, and 100 s (+1.4 V Ag|AgCl). The scale bars are 400 µm. (b, d, f) ECL histograms for the micrographs in (a), (c), and (e), respectively (green vs. blue, away vs close to the insulator regions of interest, 10 × 1200 px).

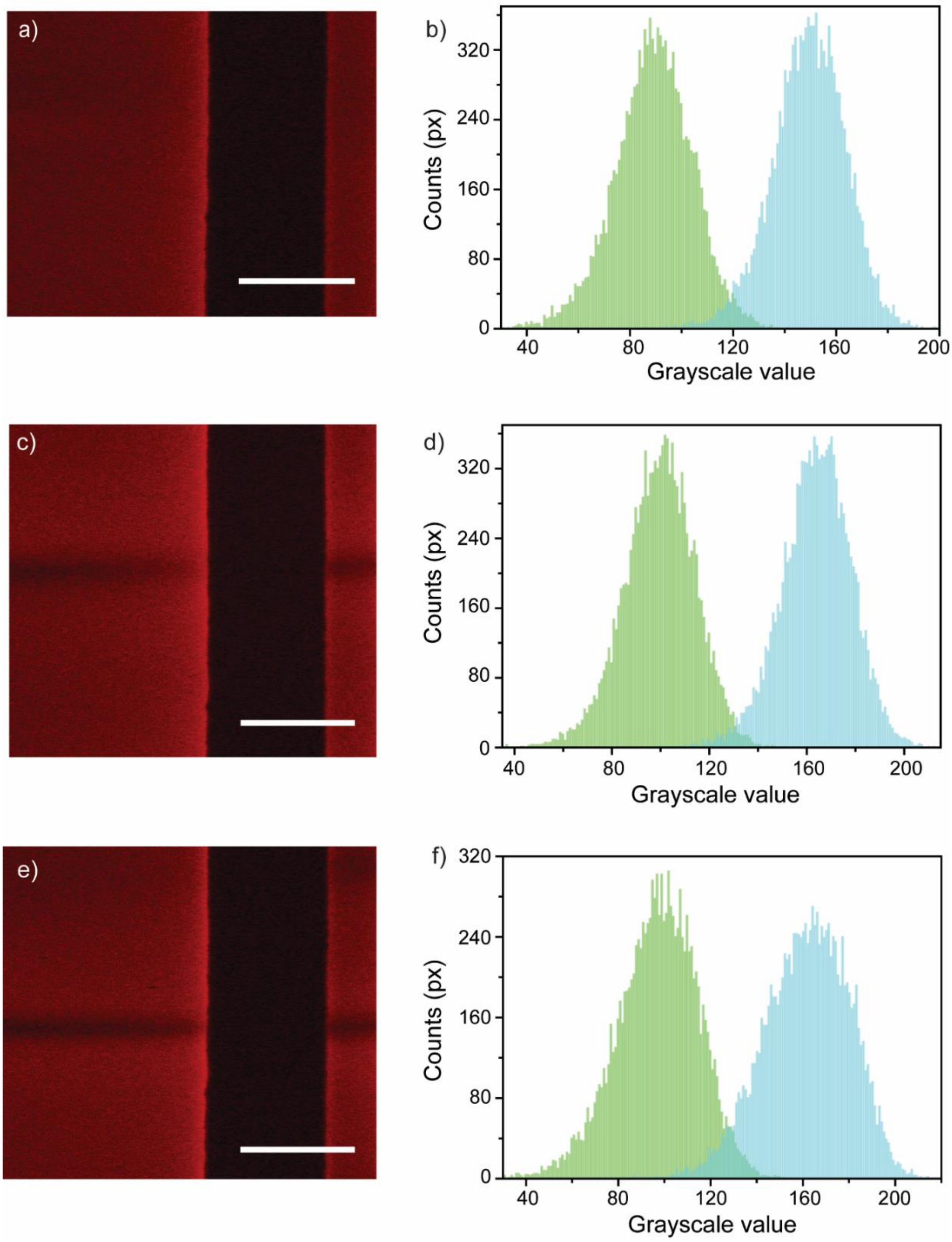

Figure S39. Micrographs and histograms for triplicate ECL microscopy experiments in fluoride-based electrolytes. The ECL solution was 4 mM $[Ru(bpy)_3]^{2+}$ and 24 mM oxalate in 0.1 M NaF at pH 6 (adjusted by dropwise addition of 10 M NaOH). ITO glass partially fouled with NLOF 2035 photoresist line-shaped features (thickness 3 µm) serves as a working electrode. The reference and counter electrodes are Ag|AgCl in 3.4 M KCl and a Pt coil, respectively. (a, c, e) ECL micrographs (2×, inverted microscope) of independently prepared and analyzed samples, corresponding to electrolysis times of 100, 90, and 110 s (+1.4 V Ag|AgCl). The scale bars are 400 µm. (b, d, f) ECL histograms for the micrographs in (a), (c), and (e), respectively (green vs. blue, away vs close to the insulator regions of interest, 10 × 1200 px).

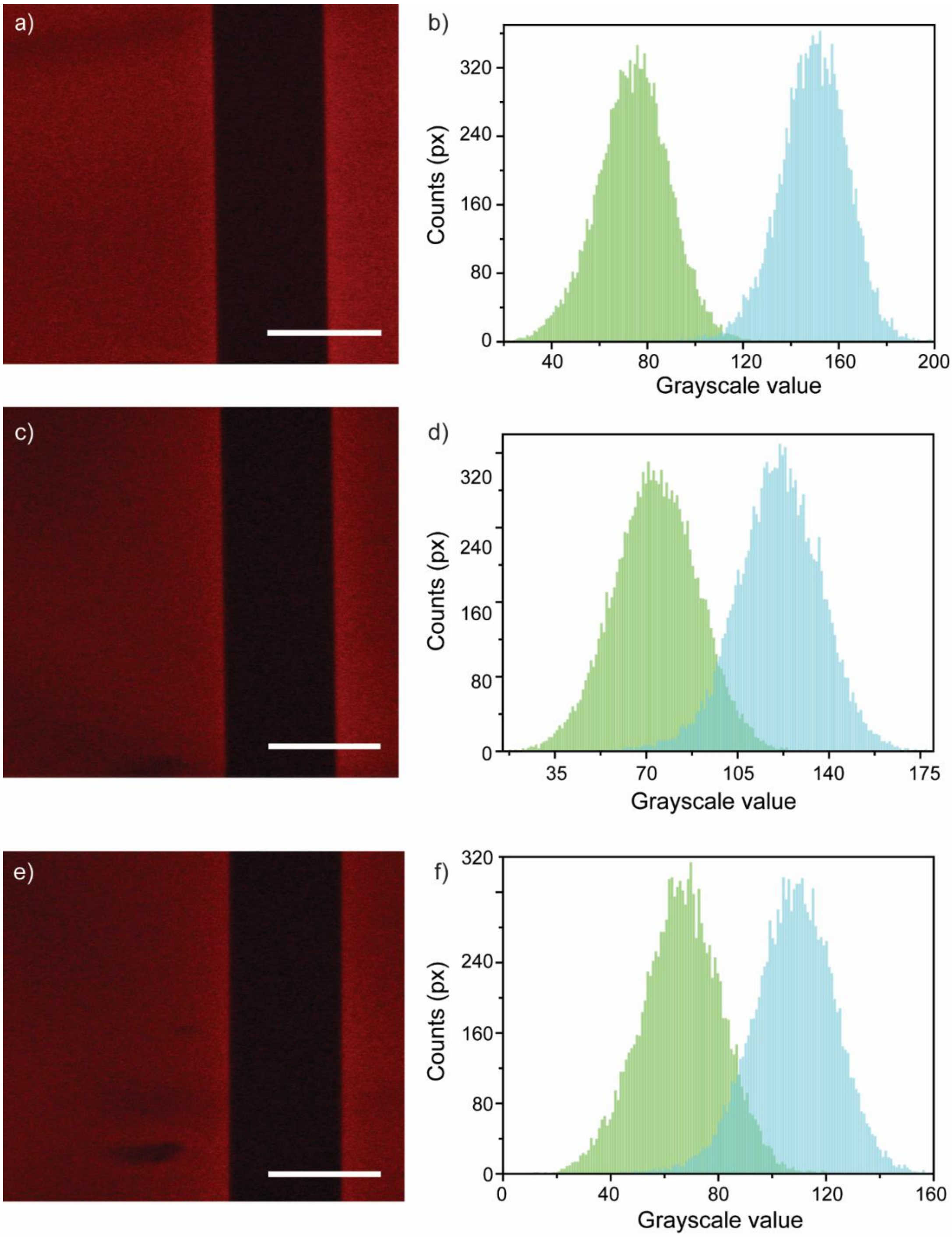

Figure S40. Micrographs and histograms for triplicate ECL microscopy experiments in acetate-based electrolytes. The ECL solution was 4 mM $[Ru(bpy)_3]^{2+}$ and 24 mM oxalate in 0.1 M CH_3COOH at pH 6 (adjusted by dropwise addition of 10 M NaOH). ITO glass partially fouled with NLOF 2035 photoresist line-shaped features (thickness 3 µm) serves as a working electrode. The reference and counter electrodes are Ag|AgCl in 3.4 M KCl and a Pt coil, respectively. (a, c, e) ECL micrographs (2×, inverted microscope) of independently prepared and analyzed samples, corresponding to electrolysis times of 70, 80, and 100 s (+1.4 V Ag|AgCl). The scale bars are 400 µm. (b, d, f) ECL histograms for the micrographs in (a), (c), and (e), respectively (green vs. blue, away vs close to the insulator regions of interest, 10 × 1200 px).

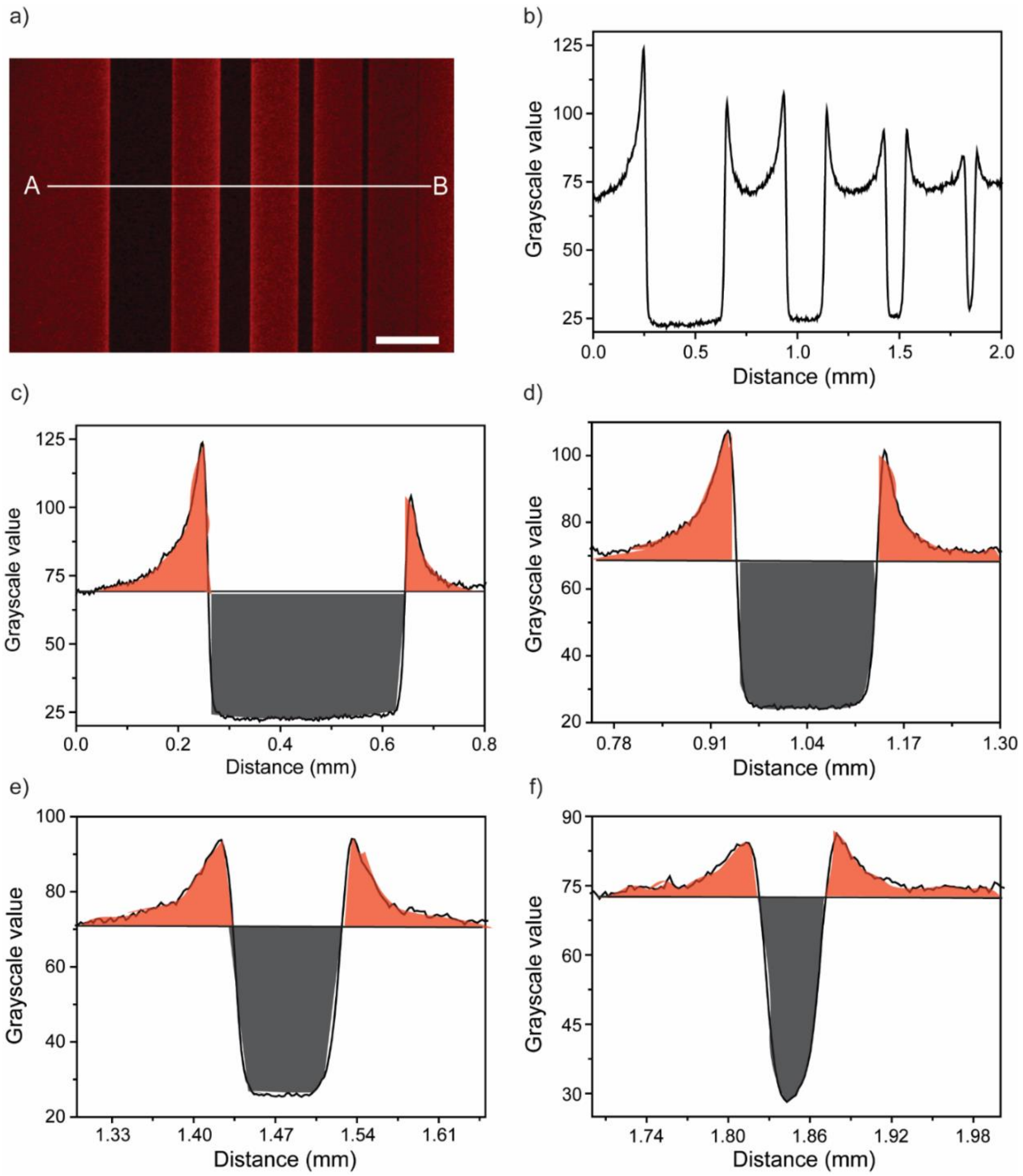

Figure S41. ECL intensity augmentation as a function of the line-shaped insulating feature width. The ECL solution was 4 mM $[Ru(bpy)_3]^{2+}$ and 24 mM oxalate in 0.1 M H_2SO_4 at pH 6. The ITO glass is partially fouled with NLOF 2035 photoresist (thickness 3 µm) features and is serving as a working electrode. The reference and counter electrodes are Ag|AgCl in 3.4 M KCl and a Pt coil, respectively. (a) ECL micrograph (2×, inverted microscope) obtained 100 s after applying a +1.4 V (vs Ag|AgCl) bias voltage. The pattern width ranges from 20 µm (right-end side of the micrograph) to 400 µm (left). The scale bar is 400 µm. (b) Overall ECL plot profile was sampled along the A–B line marked in (a). (b–f) Individual ECL plot profiles including the photoresist feature (400, 200, 100, and 50 µm line) and the adjacent clean ITO region. The red-shaded area is indicative of the ECL “gained” due to the presence of the insulator, while the blacked-out regions represent the loss in electrolysis rates, hence in ECL intensity, because of the photoresist presence.

Table S6. ECL gain-to-loss as a function of the photoresist line width.

Width (µm)	Trial 1	Trial	Trial 3	Trial 4	Trial 5
400	0.23	0.28	0.24	0.26	0.25
200	0.31	0.33	0.35	0.34	0.33
100	0.52	0.45	0.53	0.56	0.51
50	0.60	0.62	0.59	0.68	0.62

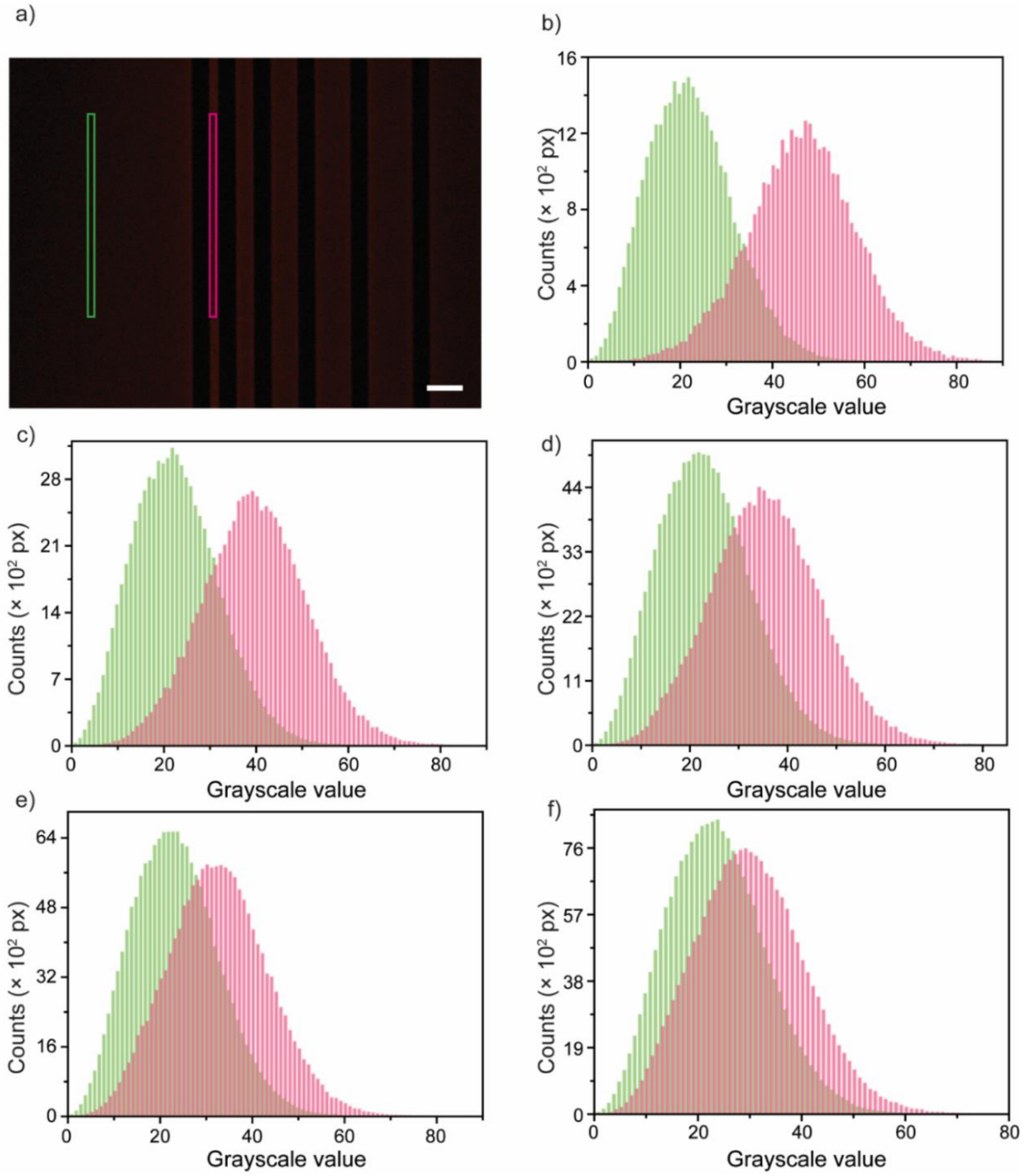

Figure S42. Micrographs and histograms for 50 µm wide lines of NLOF 2035: varying the insulator–insulator distance. The ECL solution was 4 mM $[Ru(bpy)_3]^{2+}$ and 24 mM oxalate in 0.1 M H_2SO_4 at pH 6. The ITO glass is partially fouled with features of NLOF 2035 (thickness 3 µm, 50 µm wide patterns) and it serving as the working electrode. The reference and counter electrodes are Ag|AgCl in 3.4 M KCl and a Pt coil, respectively. (a) ECL micrograph (2×, inverted microscope) recorded 100 s after applying +1.4 V (Ag|AgCl). The scale bar is 100 µm. (b–f) ECL histograms at regions of interest located in the gaps between the insulating features (25, 50, 75, 100 and 125 µm gap width, respectively). Green (away from the insulator) vs. pink (near the insulator and in the gap) regions of interest, each measuring 10 × 1200 px. The size of the boxes was changed according to the spacing between the patterns, but the length remained unchanged at 1200 px.

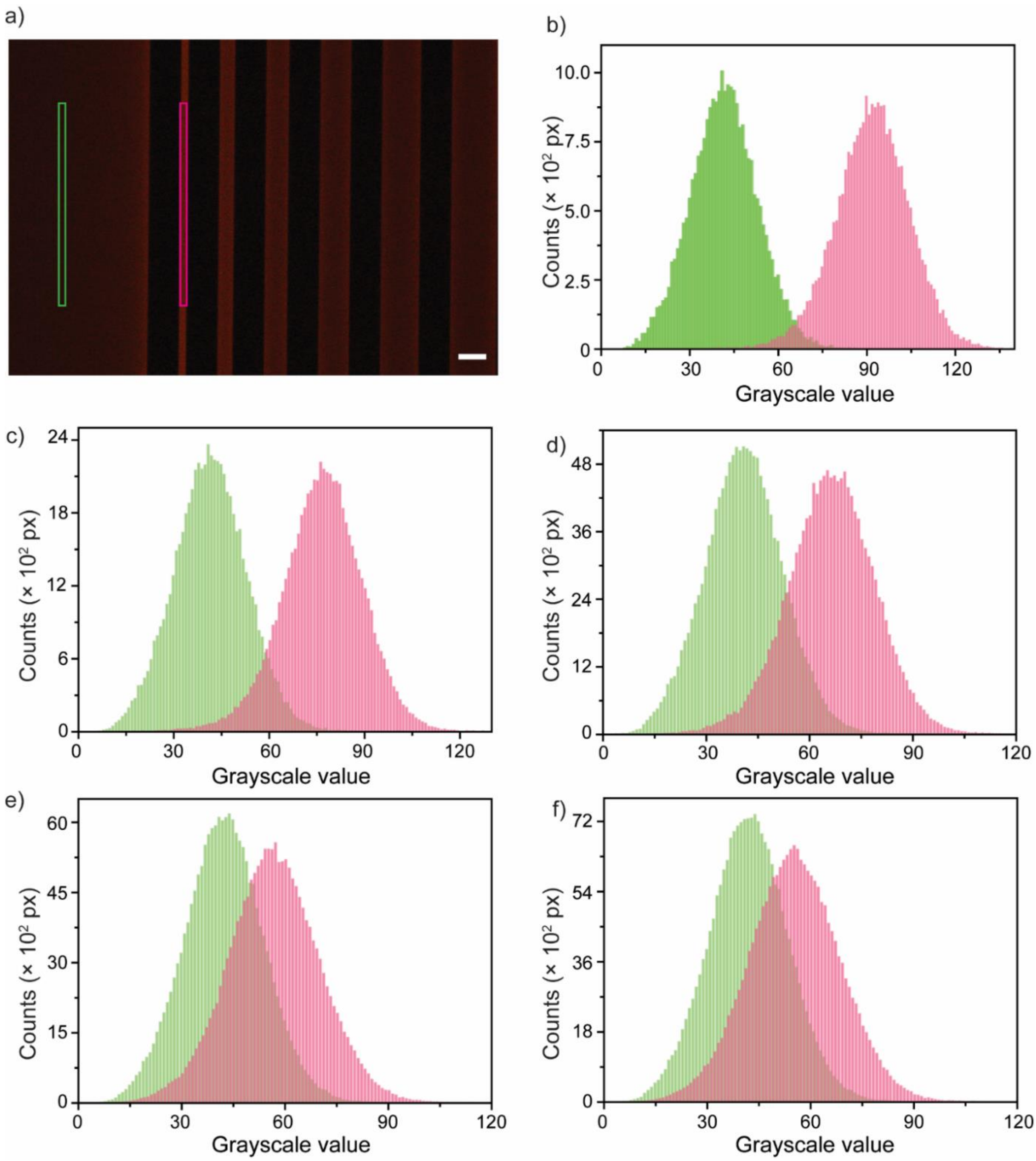

Figure S43. Micrographs and histograms for 100 µm wide lines of NLOF 2035: varying the insulator–insulator distance. The ECL solution was 4 mM $[Ru(bpy)_3]^{2+}$ and 24 mM oxalate in 0.1 M H_2SO_4 at pH 6. The ITO glass is partially fouled with features of NLOF 2035 (thickness 3 µm, 100 µm wide patterns) and it serving as the working electrode. The reference and counter electrodes are Ag|AgCl in 3.4 M KCl and a Pt coil, respectively. (a) ECL micrograph (2×, inverted microscope) recorded 100 s after applying +1.4 V (Ag|AgCl). The scale bar is 100 µm. (b–f) ECL histograms at regions of interest located in the gaps between the insulating features (25, 50, 75, 100 and 125 µm gap width, respectively). Green (away from the insulator) vs. pink (near the insulator and in the gap) regions of interest, each measuring 10 × 1200 px. The size of the boxes was changed according to the spacing between the patterns, but the length remained unchanged at 1200 px.

Table S7. ECL gain-to-loss as a function of the spacing between adjacent photoresist lines.

Spacing between patterns (μm)	Trial 1	Trial	Trial 3	Trial 4	Trial 5
25	1.9	2.0	1.2	2.2	1.8
50	0.6	0.7	0.5	1.0	0.7
75	0.4	0.4	0.4	0.6	0.5
100	0.4	0.3	0.4	0.4	0.4
125	0.3	0.2	0.3	0.3	0.3

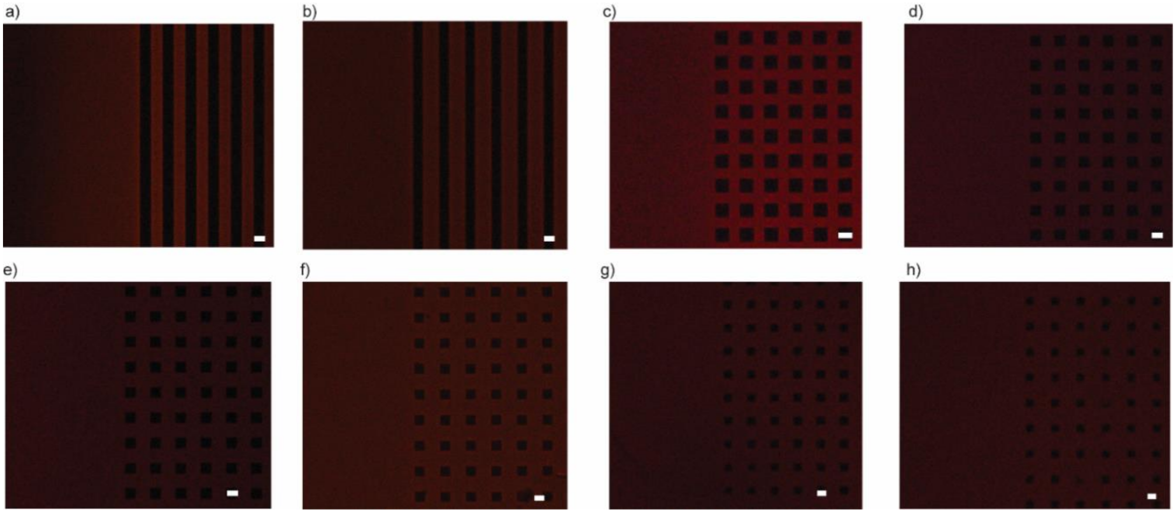

Figure S44. ECL micrographs of ITO electrodes partially fouled by 50 μm wide insulating features separated by clean electrode gaps of different sizes. ECL micrographs (2×) captured during the anodic electrolysis of aqueous solution of 4 mM of $[Ru(bpy)_3]^{2+}$ and 24 mM oxalate in 0.1 M H_2SO_4 at pH 6. The working electrode is ITO–glass biased at +1.4 V (vs Ag|AgCl, 3.4 M KCl). The electrolysis time for the ECL images in the figure ranged between 70 and 110 s. The scale bar is 50 μm. (a) 50 μm NLOF 2035 lines with 50 μm spacing between them. (b) 50 μm NLOF 2035 lines with 75 μm spacing. (c) 50 μm NLOF 2035 squares with 35 μm spacing. (d) 50 μm NLOF 2035 squares with 50 μm spacing. (e) 50 μm NLOF 2035 squares with 60 μm spacing. (f) 50 μm SU–8 2002 squares with 60 μm spacing. (g) 50 μm NLOF 2035 squares with 65 μm spacing. (h) 50 μm SU–8 2002 squares with 65 μm spacing.

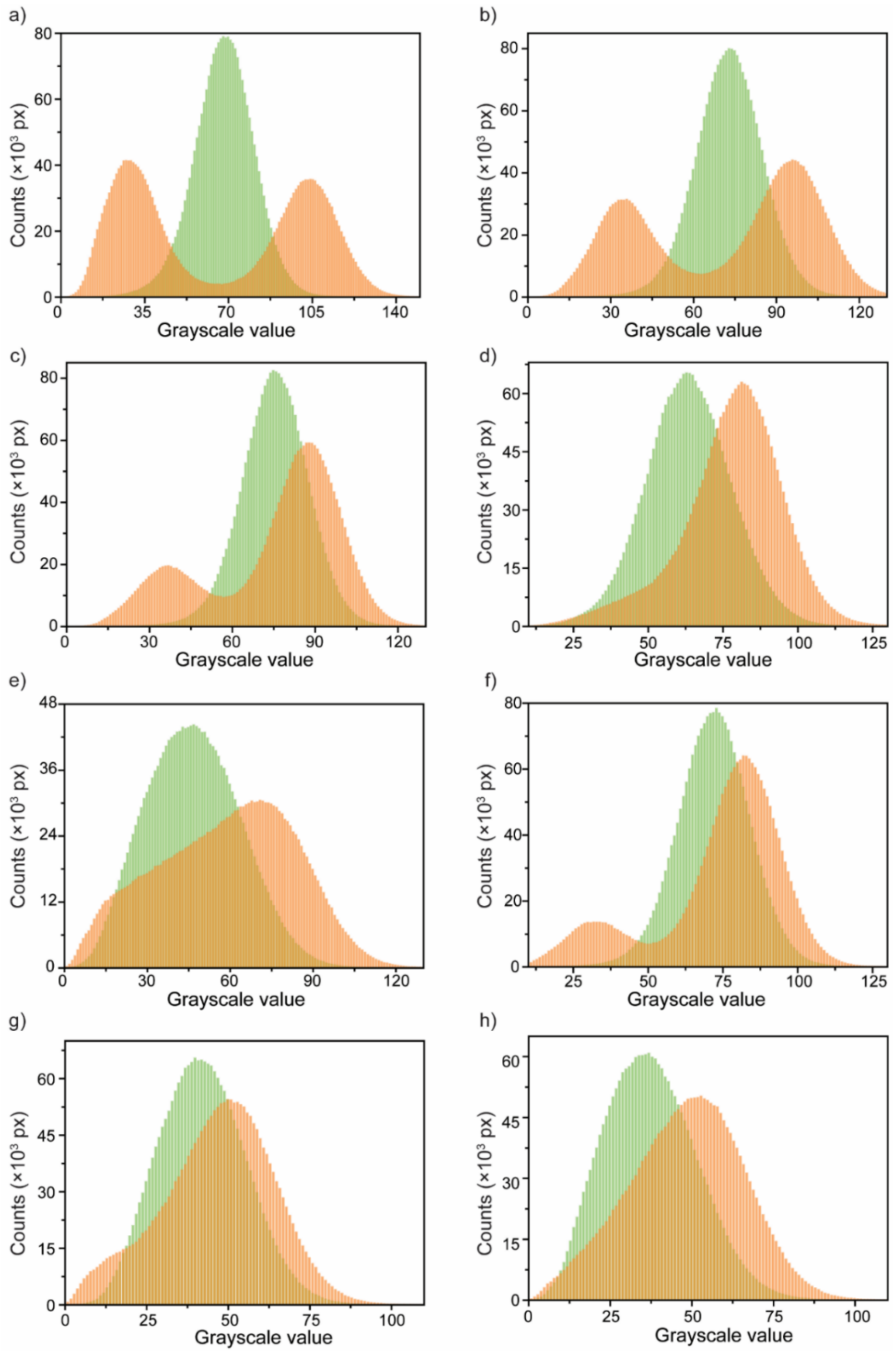

Figure S45. ECL intensity histograms from the micrographs of ITO electrodes partially fouled by 50 µm wide insulating features separated by clean electrode gaps of different size. ECL micrographs (2×) captured during the anodic electrolysis of n aqueous solution of 4 mM of $[Ru(bpy)_3]^{2+}$ and 24 mM oxalate in 0.1 M H_2SO_4 at pH 6, adjusted using 10 M NaOH at an ITO–glass electrode biased at 1.4 V (vs Ag|AgCl, 3.4 M KCl). Images were captured using a Nikon ECLIPSE Ti2-U inverted microscope with a 5.9-megapixel CMOS color camera (DS-Fi3, Nikon). Electrolysis times are between 70 s to 110 s. (a) 50 µm lines with 50 µm spacing for NLOF 2035 (b) 50 µm lines with 75 µm spacing for NLOF 2035 (c) 50 µm squares with 35 µm spacing for NLOF 2035 (d) 50 µm squares with 50 µm spacing for NLOF 2035 (e) 50 µm squares with 60 µm spacing for NLOF 2035 (f) 50 µm squares with 60 µm spacing for SU–8 2002 (g) 50 µm squares with 65 µm spacing for NLOF 2035 (h) 50 µm squares with 65 µm spacing for SU–8 2002

Table S8. ECL gain-to-loss as a function of NLOF and SU–8 2002 photoresist geometry (size, spacing between adjacent photoresist pattern, and shape).

	Square size (µm)	Space between squares (µm)	Gain-to-loss ratio
NLOF 2035 square patterns	25	25	1.1
	25	35	1.1
	25	50	1.1
	25	65	1.2
	25	75	1.1
	50	35	1.0
	50	50	1.0
	50	60	1.2
	50	65	1.2
	50	75	1.0
NLOF 2035 line	50	50	1.0
	50	75	1.0
SU square patterns	25	50	1.1
	25	65	1.1
	50	60	1.1
	50	65	1.3

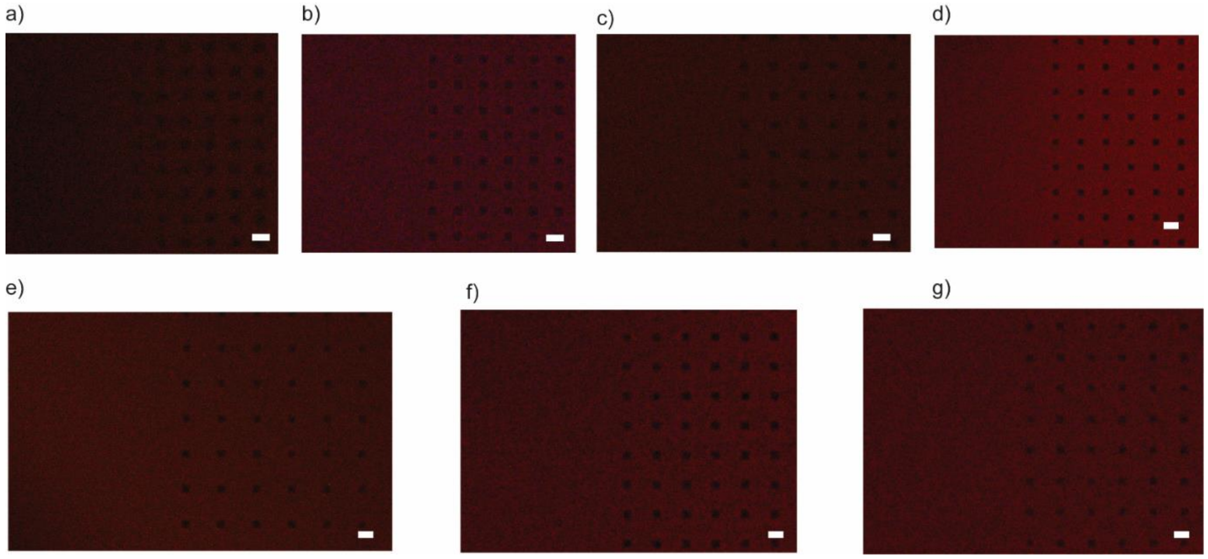

Figure S46. ECL micrographs of ITO electrodes partially fouled by 25 µm wide insulating features separated by clean electrode gaps of different sizes. ECL micrographs (2×) captured during the anodic electrolysis of aqueous solution of 4 mM of $[Ru(bpy)_3]^{2+}$ and 24 mM oxalate in 0.1 M H_2SO_4 at pH 6. The working electrode is ITO–glass biased at +1.4 V (vs Ag|AgCl, 3.4 M KCl). The electrolysis time for the ECL images in figure ranged between 70 and 110 s. The scale bar is 50 µm. (a) 25 µm NLOF 2035 squares with 25 µm spacing between them. (b) 25 µm NLOF 2035 squares with 35 µm spacing. (c) 25 µm NLOF 2035 squares with 50 µm spacing. (d) 25 µm SU–8 2002 squares with 50 µm spacing. (e) 25 µm NLOF 2035 squares with 65 µm spacing. (f) 25 µm SU–8 2002 squares with 65 µm spacing. (g) 25 µm NLOF 2035 squares with 75 µm spacing.

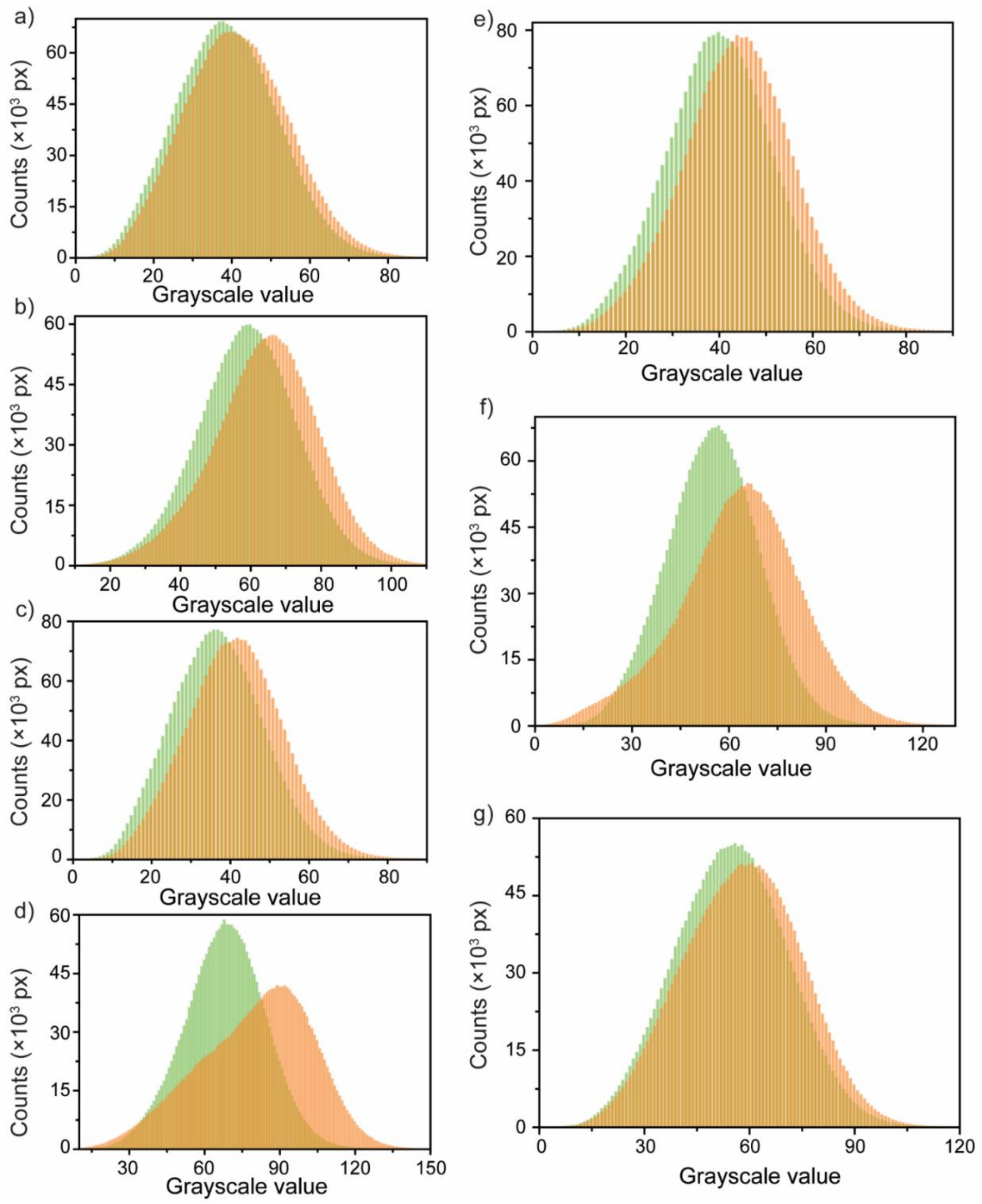

Figure S47. ECL intensity histograms at partially fouled ITO electrodes with 25 µm wide and different spacing. ECL micrographs (2×) captured during the anodic electrolysis of n aqueous solution of 4 mM of $[Ru(bpy)_3]^{2+}$ and 24 mM oxalate in 0.1 M H_2SO_4 at pH 6, adjusted using 10 M NaOH at an ITO–glass electrode biased at 1.4 V (vs Ag|AgCl, 3.4 M KCl). Images were captured using a Nikon ECLIPSE Ti2-U inverted microscope with a 5.9-megapixel CMOS color camera (DS-Fi3, Nikon). Electrolysis times are between 70 s to 110 s. (a) 25 µm squares with 25 µm spacing for NLOF 2035 (b) 25 µm squares with 35 µm spacing for NLOF 2035 (c) 25 µm squares with 50 µm spacing for NLOF 2035 (d) 25 µm squares with 50 µm spacing for SU–8 2002 (e) 25 µm squares with 65 µm spacing for NLOF 2035 (f) 25 µm squares with 65 µm spacing for SU–8 2002 (g) 25 µm squares with 75 µm spacing for NLOF 2035

Table S9. ECL gain-to-loss as a function of the size and the spacing between adjacent NLOF 2035 and SU–8 2002 photoresist squares.

	NLOF 2035						SU-8 2002			
Time (s)	25 µm 35 µm	25 µm 50 µm	25 µm 65 µm	50 µm 50 µm	50 µm 60 µm	50 µm 65 µm	25 µm 50 µm	25 µm 65 µm	50 µm 60 µm	50 µm 65 µm
30	1.0	1.0	1.0	1.0	1.2	1.0	0.9	1.0	1.0	1.1
32	1.0	1.0	1.0	1.0	1.2	1.0	0.9	1.0	1.0	1.1
34	1.0	1.0	1.0	1.0	1.2	1.0	0.9	1.0	1.0	1.1
36	1.0	1.0	1.0	1.0	1.2	1.0	1.0	1.0	1.0	1.1
38	1.0	1.0	1.0	1.0	1.2	1.0	1.0	1.0	1.1	1.1
40	1.0	1.0	0.9	1.0	1.2	1.0	1.0	1.0	1.1	1.1
42	1.0	1.0	0.9	1.0	1.2	1.0	1.0	1.0	1.1	1.1
44	1.0	1.0	0.9	1.0	1.2	1.0	1.0	1.0	1.1	1.1
46	1.0	1.0	0.9	1.0	1.2	1.0	1.1	1.0	1.1	1.1
48	1.0	1.0	0.9	1.0	1.2	1.0	1.1	1.0	1.1	1.1
50	1.0	1.0	0.9	1.0	1.2	1.0	1.1	1.0	1.1	1.0
52	1.0	1.0	0.9	1.0	1.2	1.0	1.1	1.0	1.1	1.0
54	1.0	1.0	0.9	1.0	1.2	1.0	1.1	1.0	1.1	1.0
56	1.0	1.0	0.9	1.0	1.2	1.0	1.1	1.0	1.1	1.0
58	1.0	1.0	0.9	1.0	1.2	1.0	1.1	1.0	1.1	1.0
60	1.0	1.0	0.9	1.0	1.2	1.0	1.1	1.0	1.0	1.0
62	1.1	1.0	0.9	1.0	1.2	1.0	1.1	1.0	1.0	1.0
64	1.1	1.0	0.9	1.0	1.2	1.0	1.1	1.0	1.0	1.0
66	1.1	1.0	0.9	1.0	1.2	1.0	1.1	1.0	1.0	1.0
68	1.1	1.0	1.0	1.0	1.2	1.0	1.1	1.0	1.0	1.0
70	1.1	1.0	1.0	0.9	1.2	1.0	1.1	1.0	1.0	1.0
72	1.1	1.1	1.0	0.9	1.2	1.0	1.0	1.0	1.0	1.0
74	1.1	1.1	1.0	0.9	1.2	1.0	1.0	1.1	1.0	1.0
76	1.1	1.1	1.0	0.9	1.2	1.0	1.0	1.1	1.0	1.0
78	1.1	1.1	1.0	0.9	1.2	1.0	1.0	1.1	0.9	1.0
80	1.1	1.1	1.0	0.9	1.1	1.0	1.0	1.1	0.9	1.0
82	1.1	1.1	1.0	0.9	1.2	1.0	1.0	1.1	0.9	1.0
84	1.1	1.1	1.1	0.9	1.1	1.0	1.0	1.1	0.9	1.0
86	1.1	1.1	1.1	0.9	1.1	1.0	1.0	1.1	0.9	1.0
88	1.1	1.1	1.1	0.9	1.1	1.0	1.0	1.0	0.9	1.1
90	1.1	1.1	1.1	0.9	1.1	1.0	1.0	1.0	0.9	1.1
92	1.0	1.1	1.1	0.9	1.1	1.0	1.0	1.0	0.9	1.2
94	1.0	1.1	1.2	0.9	1.1	1.0	1.0	1.0	0.9	1.2
96	1.0	1.1	1.2	0.9	1.1	1.0	1.0	1.0	0.9	1.3
98	1.0	1.1	1.2	1.0	1.1	1.0	1.0	1.0	1.0	1.3
100	1.0	1.1	1.2	1.0	1.1	1.1	1.0	0.9	1.0	1.3

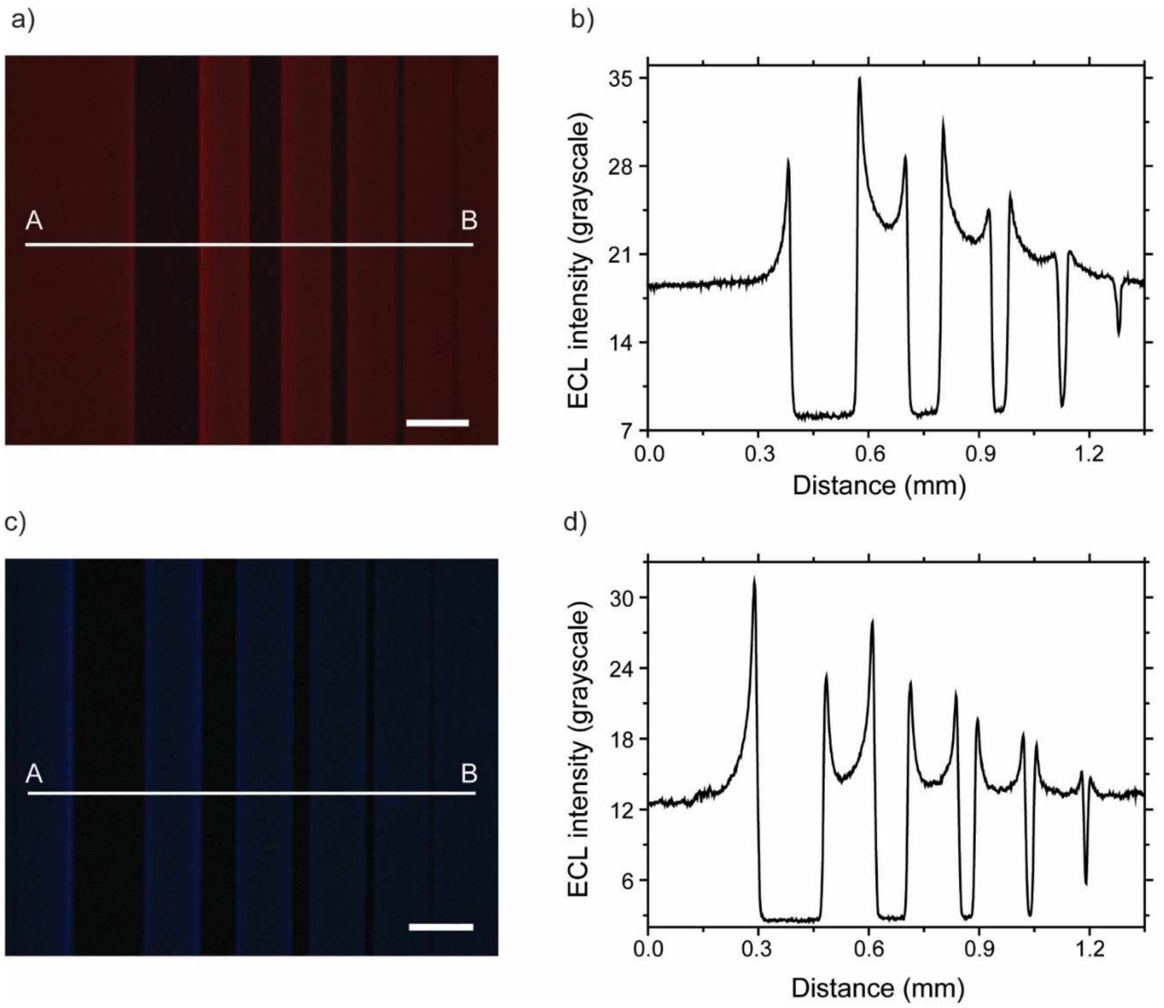

Figure S48. Near-insulator rates augmentation for alternative ECL systems. ECL microscopy at ITO glass partially fouled with photolithographic SU-8 2002 photoresist features (thickness, 3 µm; widths ranging from 25 to 400 µm) serving as the working electrode. The reference and counter electrodes are Ag|AgCl in 3.4 M KCl and a platinum coil, respectively. (a,b) Experiments with 1 mM $[Ru(bpy)_3]^{2+}$, 10 mM TPrA and 100 mM $TBAPF_6$ (tetrabutylammonium hexafluorophosphate) in acetonitrile. (a) Representative ECL micrograph (2×, inverted microscope) obtained 70 s after applying a +1.4 V (vs Ag|AgCl) bias voltage. (b) ECL plot profile sampled along the A–B line marked in (a). (c,d) Experiments with 50 mM luminol, 100 mM NaOH and 0.3% (v/v) H_2O_2 in water. (c) ECL micrograph (2×, inverted microscope) obtained 60 s after applying a +1.0 V (vs Ag|AgCl) bias voltage. (d) ECL plot profile sampled along the A–B line marked in (c). Scale bars in (a,c) are 400 µm. The photoresist line widths of the micrographs in (a,c) ranged from 25 µm (right-end side) to 400 µm (left).

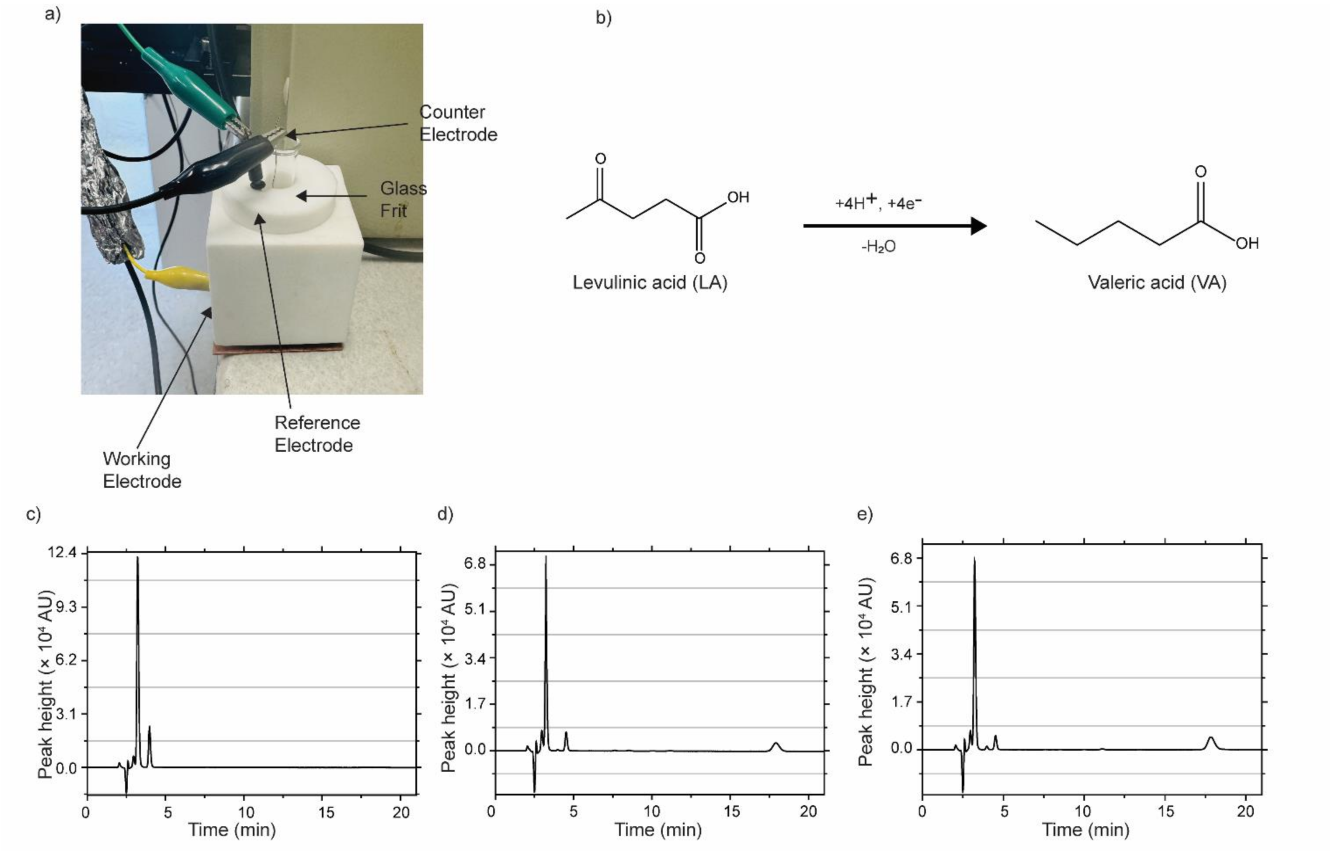

Figure S49. Electrochemical reduction of levulinic acid (LA) to valeric acid (VA): clean vs. fouled electrodes. (a) Image of the divided PTFE cell used for the four-electron, four-proton conversion of levulinic acid (LA) into valeric acid (VA), as schematically shown in (b). A glassy carbon (GC) plate served as the working electrode, a platinum coil as counter electrode, and an Ag|AgCl (3.4 M KCl) as reference electrode. The cell was divided by a glass frit, with the counter electrode compartment filled with 0.5 M H_2SO_4. The GC electrode was either unmodified or patterned across its entire surface by NLOF 2035 features (50 µm-wide squares separated by 60 µm gaps of clean GC surface). The GC cathodic bias was set to −1.8 V vs. the reference, and the electrolysis time was 60 min. (c–e) HPLC chromatograms of the electrolytic solution before (c) and after (d,e) the cathodic electrolysis of aqueous 0.5 M H_2SO_4 (5 mL) containing 0.2 M LA. The HPLC system was equipped with a reverse-phase column (C18, 150 × 2 mm, 5 µm, Agilent) and a photodiode array detector (PDA). The traces shown in (c–e) correspond to absorbance monitored at 212 nm by the PDA. Chromatograms were obtained by injecting 5 µL of the crude reaction mixture and eluting at a flow rate of 0.2 mL/min^{-1} using a mobile phase composed of 10% acetonitrile and 90% Milli-Q™ water. The chromatogram in (d) is that of samples electrolyzed at a clean, unmodified GC electrode, and that in (e) is for samples electrolyzed with a patterned GC electrode. VA elutes at 17.9 min, and LA around 2.6 min. The height of the VA band was 2862 A.U. for the sample electrolyzed on unmodified GC (d), and 4563 A.U. for the sample electrolyzed on NLOF-patterned GC (e).

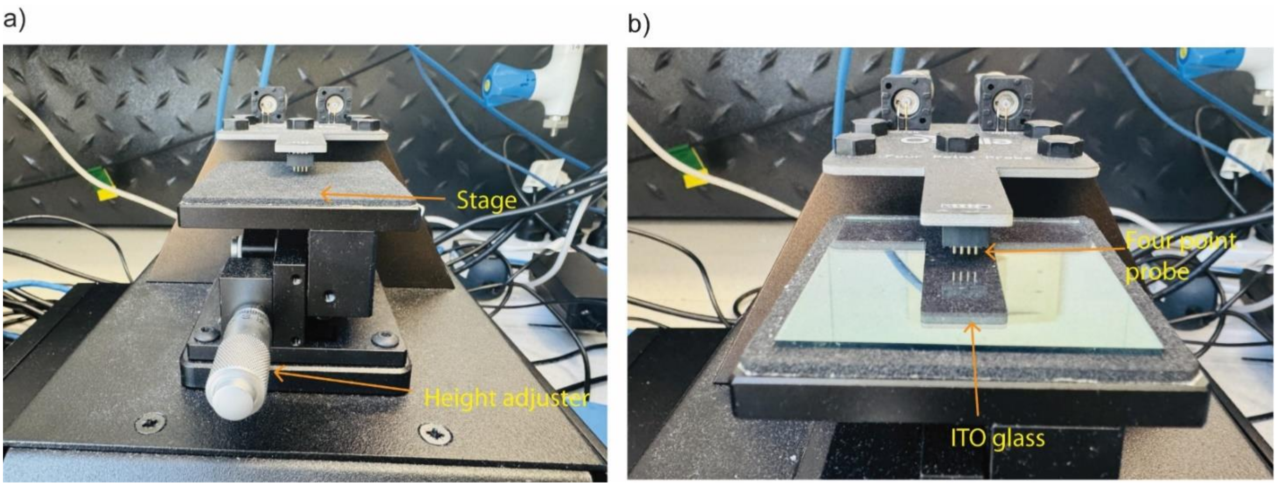

Figure S50. Four-point probe setup used to measure the resistivity of the ITO glass. (a) Experimental setup prior to loading of the sample. Four equally spaced probes are visible at the center of the picture. (b) Probes placed in contact with the ITO-glass surface. The two outer probes force a known current across the sample, while the inner (middle) two probes measure the resulting voltage drop across the material.

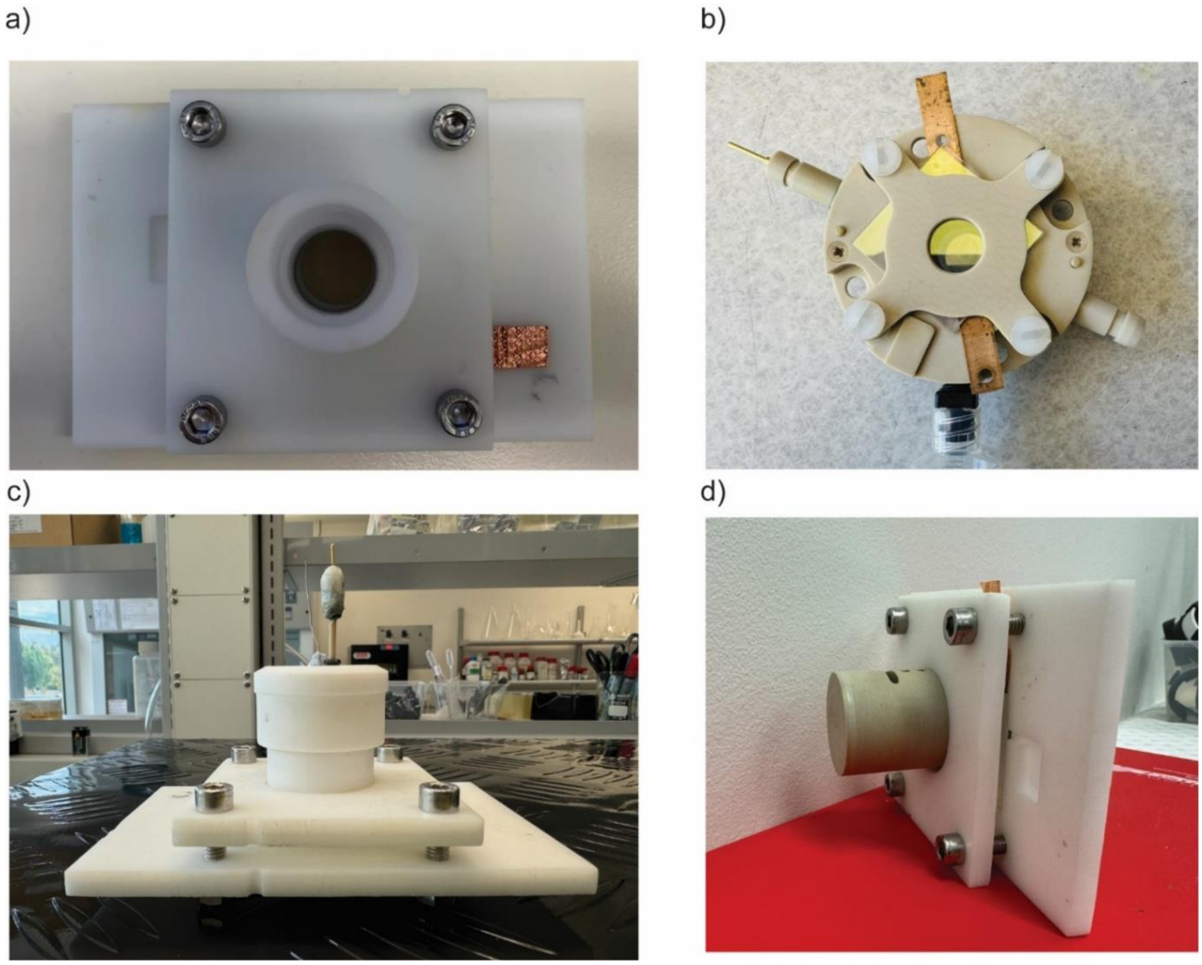

Figure S51. Custom-designed cells for spectroelectrochemical, microscopy, and cyclic voltammetry studies. (a, c) The horizontal cell design is used for electrochemiluminescence (ECL) microscopy, spectroelectrochemical measurements, and photon counting experiments. This cell was specifically designed to accommodate transparent electrodes. (b) The custom cell is used for non-transparent electrodes, such as glassy carbon electrodes. This cell has a smaller volume compared to the horizontal cell of (a, c). (d) Vertical cell setup for large samples.

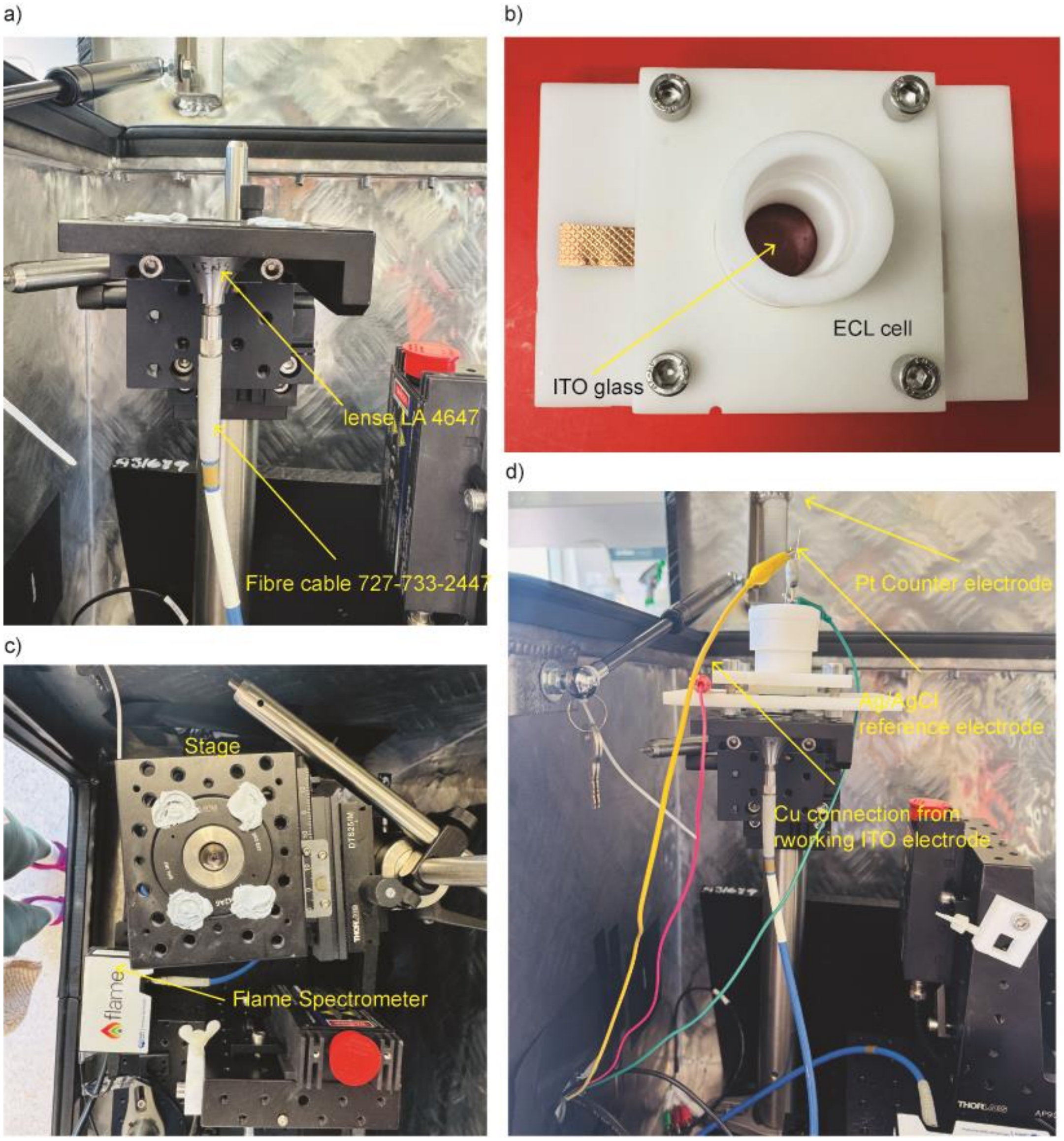

Figure S52. Spectroelectrochemistry setup. (a) The optical system consists of a lens (LA 4647) attached to a fiber optic cable, which is used to collect the light emitted during electrochemical reactions. The lens is positioned to focus the light onto an opical fiber cable for efficient light transmission to the spectrometer (a fiber-coupled diode array spectrometer, Flame Miniature Spectrometer, Ocean Optics, United States). (b) The type of electrochemical cell used for the measurements depends on the electrode geometry and transparency. In (b) is a picture of the cell used with ITO working electrodes. For platinum (Pt) electrodes, a modified quartz cuvette was used, while for glassy carbon electrodes the custom cell is shown in Figure S54b (vertical configuration). (c) Image of the stage used to hold the horizontal electrochemical cell. (d) Side view of the cell mounted on the stage, showing the ITO glass electrode and all associated electrical connections for the spectroelectrochemical measurement.

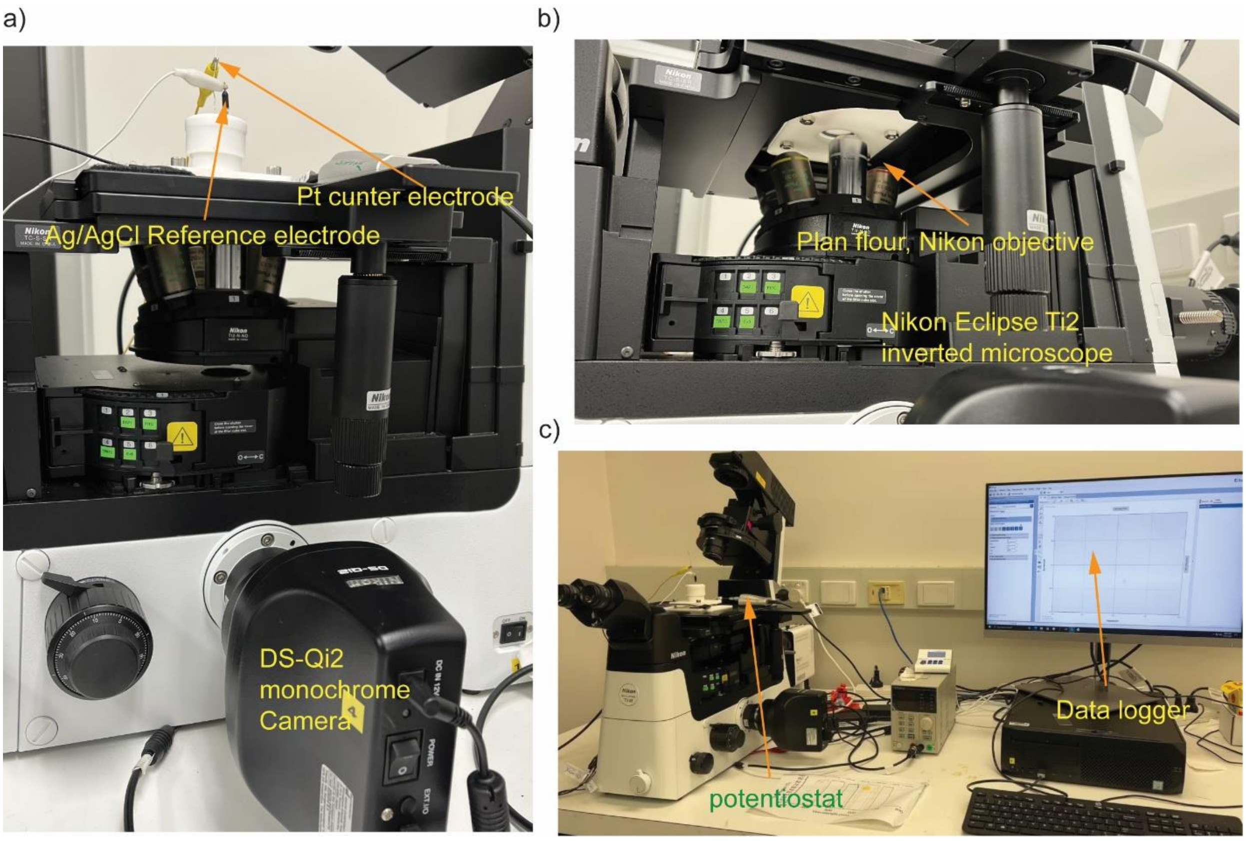

Figure S53. Inverted microscope-based ECL setup. Key components of the microscopy setup used for the ECL imaging experiments (described in further detail in the main text experimental section). (a–d) Ther Nikon ECLIPSE Ti2-U inverted microscope is equipped with both a 16.25-megapixel CMOS monochrome camera (DS-Qi2, Nikon) and a 5.9-megapixel CMOS color camera (DS-Fi3, Nikon). For the purposes of these experiments, only the 5.9-megapixel CMOS color camera (DS-Fi3, Nikon) was used to capture high-resolution images of the ECL emissions. The reference electrode was an Ag|AgCl electrode in 3.4 M KCl, while the counter electrode was a Pt coil.

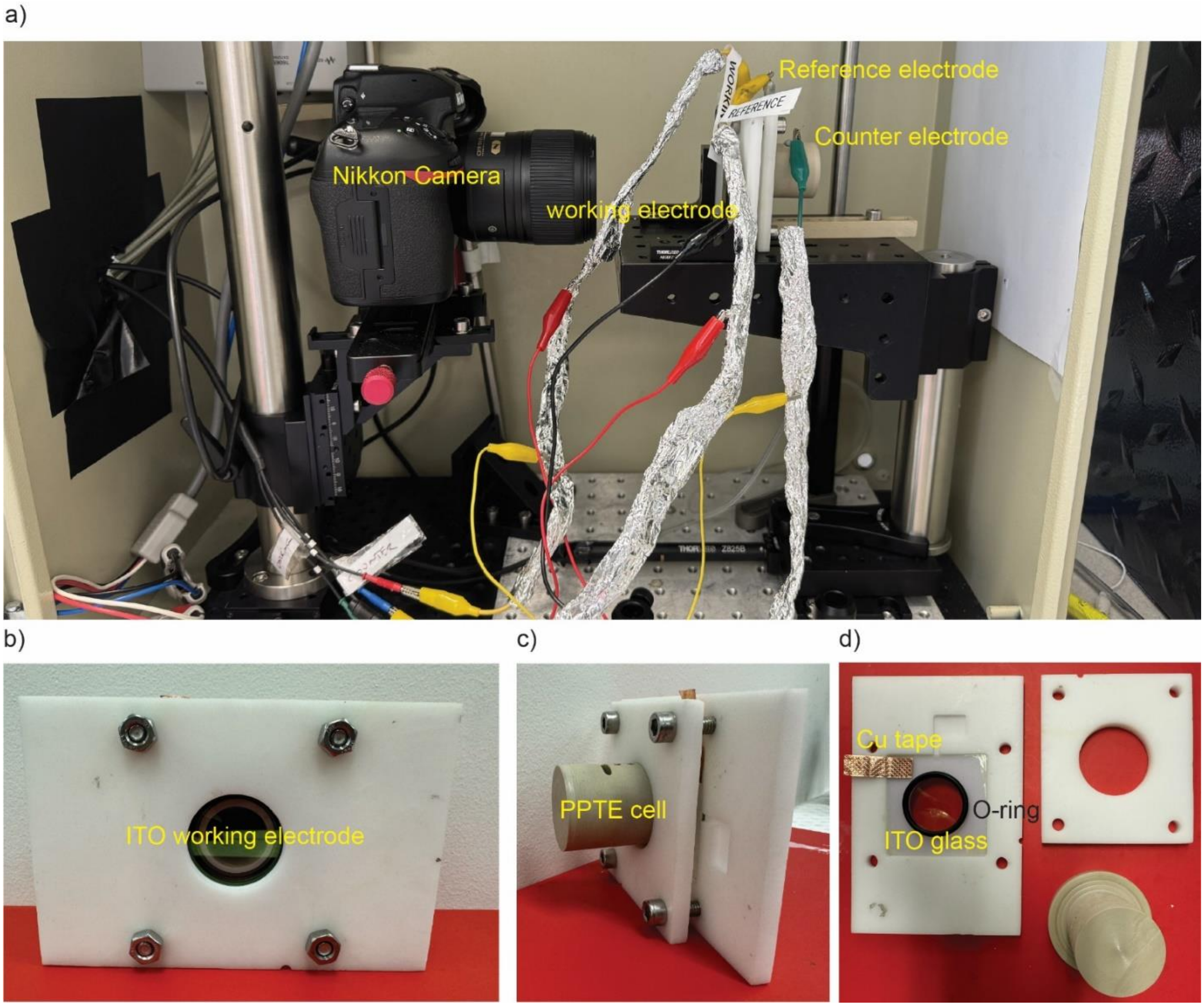

Figure S54. Vertical cell to validate the density gradient-driven convection in the ECL system. The ECL system was 4 mM of $[Ru(bpy)_3]^{2+}$ and 24 mM oxalate in 0.1 M H_2SO_4 at pH 6. The ITO glass was served as the working electrode, the reference electrode was Ag|AgCl in 3.4 M KCl, and the counter electrode was a Pt mesh. (a) The cell was mounted on an XYZ stage, facing a Nikon D850 DSLR camera (35.9 × 23.9 mm CMOS sensor) fitted with a 60 mm AF-S Micro-NIKKOR F/2.8 G ED lens. The camera was focused on the plane where the photoresist line meets the ITO surface. (b–d) Images of the single-compartment PEEK cell, of ~12 mL total liquid capacity, showing the ITO electrode from the camera-facing side and the electrical connection to ITO glass through a strip of Cu tape.

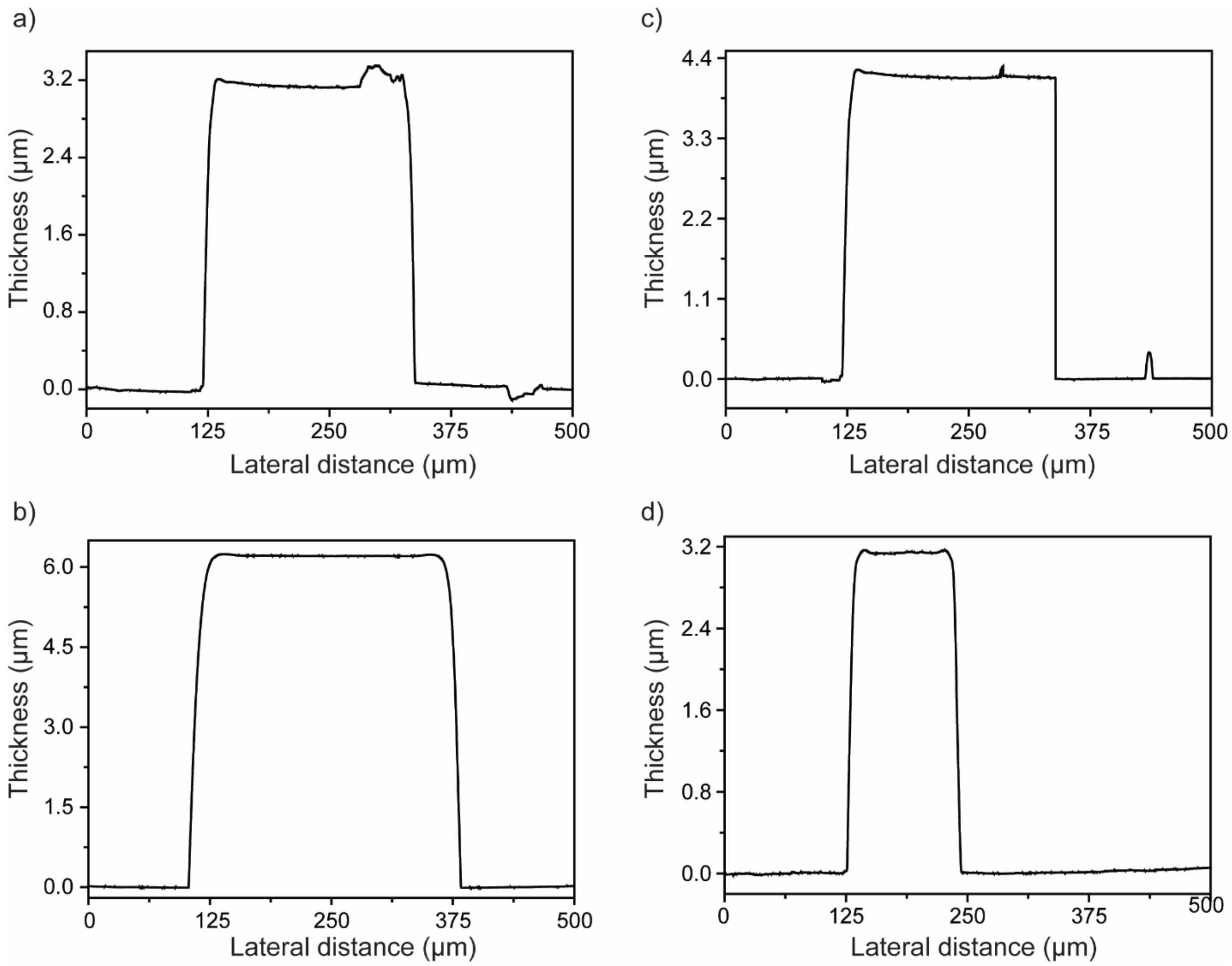

Figure S55. Profilometry analysis of the photoresist film thickness using a Dektak 150 surface profiler. Photoresist-coated ITO glass substrates were prepared at different film thicknesses following procedures reported by the manufacturer. After baking, the spin-coated photoresist was exposed to patterned UV light (375 nm, 2.8 W cm^{-2} with a maskless aligner, or 365 nm, 7.3 mW cm^{-2} for 2.5 s with a photomask). Then the post-baked samples were developed and rinsed with water. The details of the process are in the main text experimental section. Profilometry measurements were performed to experimentally validate the photoresist thickness and are as follows: (a) NLOF 2035, ~ 3.1 µm; (b) NLOF 2035, ~ 4.1 µm; (c) NLOF 2035, ~ 6.2 µm; (d) SU–8 2002, ~ 3.1 µm.

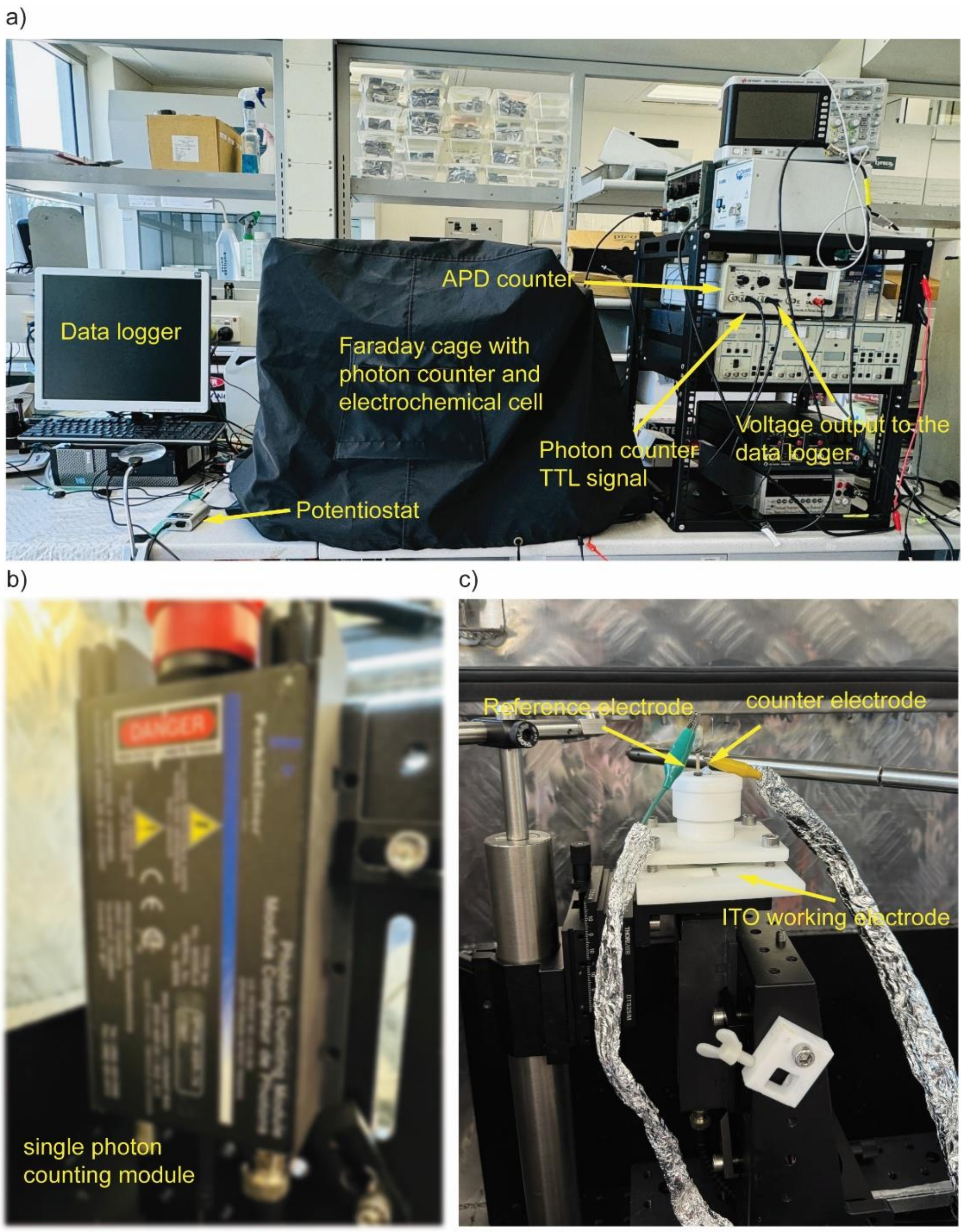

Figure S56. Photon counting setup. (a) Overview of the photon counting setup. (b) The photon counting system is centered around the PerkinElmer SPCM-AQR-14 Single Photon Counting Module, which utilizes silicon avalanche photodiodes (APDs) operating in Geiger mode. The module's BNC TTL output is connected to an avalanche photodiode (APD) counter from Nanonics Imaging Ltd., which converts the signal into a voltage output. This voltage signal is then recorded using a Pico Technology DrDAQ data logger. The SPCM-AQR-14 is powered by the APD counter, with a 5 V power supply. (c) The electrochemical compartment was controlled by an EmStat3 potentiostat (PalmSens BV, Netherlands) and consisted of a custom-made PTFE single-compartment cell. The cell was equipped with a leakless Ag/AgCl (3.4 M KCl) reference electrode from eDAQ, a platinum coil counter electrode, and an ITO-coated glass working electrode.

a)

b)

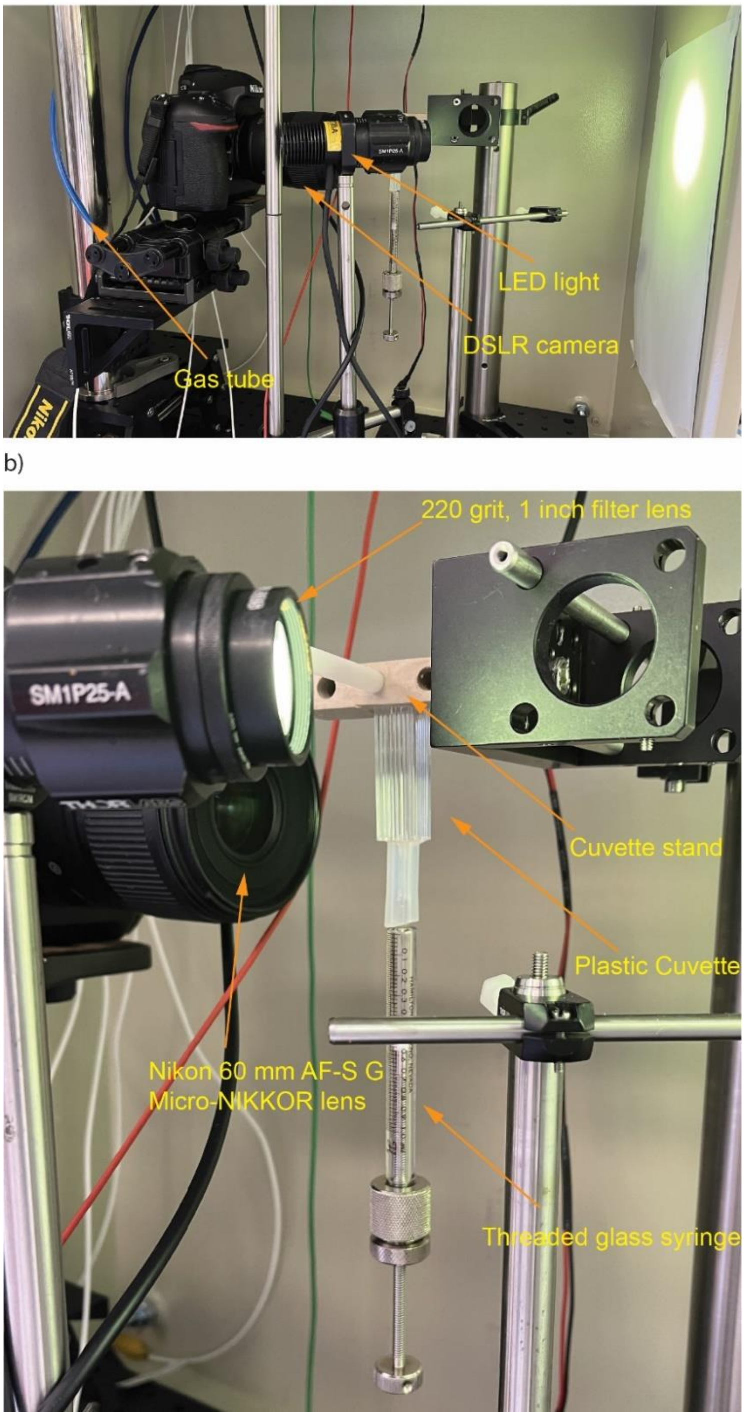

Figure S57. Experimental setup used for the measurement of surface tension. The shape and size of a N_2 gas bubble, forced into a liquid sample held in a two-clear side cuvette, using a threaded plunger glass syringe through a glass capillary was imaged with back-lit CMOS DSLR camera (Nikon D850, Nikon 60 mm AF-S G Micro-NIKKOR lens), recording a frame approximately every 60 ms. Further details in the main text experimental section.

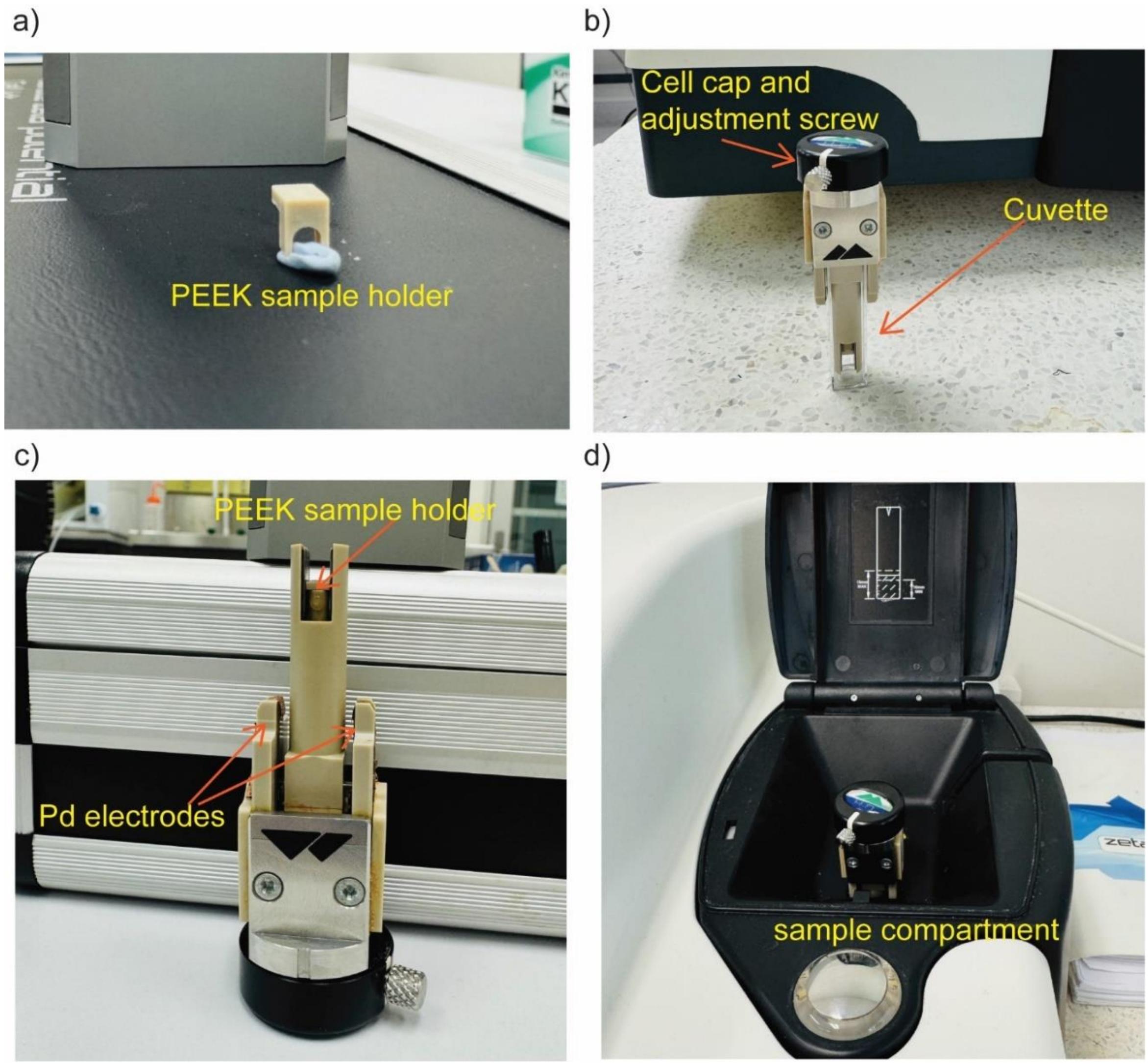

Figure S58. Surface Zeta potential measurement setup using a Malvern Zetasizer. Full instrumental details and methods are in the main text experimental section. (a) Image of the PEEK sample holder which was coated with the photoresists sample (NLOF-2035 or SU–8 2002). (b) Apparatus to mount and control the vertical position of the sample during the experiment. (c) The photoresist-coated PEEK sample holder was mounted on the adapter and then placed in the sample compartment as shown in (d). The movement of tracer particles near the film is measured by a Malvern Zetasizer.

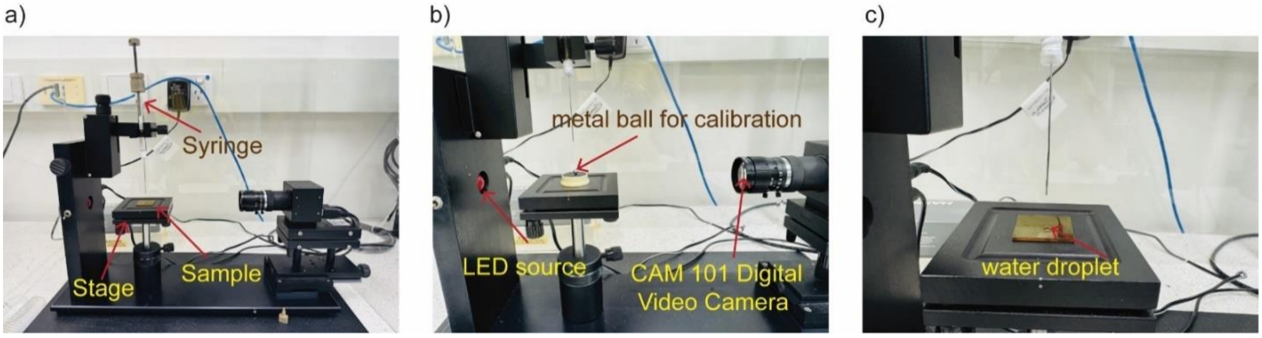

Figure S59. Contact angle measurements setup. The images in the figure show the experimental setup used to measure the static contact angle of a liquid droplet on a solid surface. (a) The syringe stage and sample are shown, where a water droplet is dispensed onto the surface of the sample for contact angle measurement. (b) The metal ball used for calibration, along with the LED light source and CAM 101 digital video camera, captures the droplet profile for accurate contact angle analysis. (c) A water droplet is visible on the sample surface. The system allows precise control of sample positioning and liquid dispensing for consistent and reproducible measurements.

a)

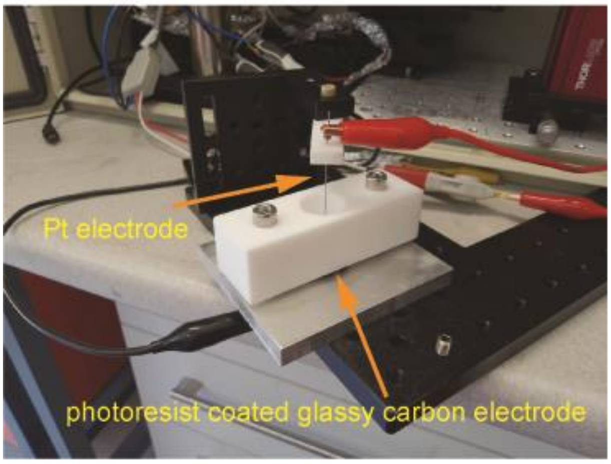

b)

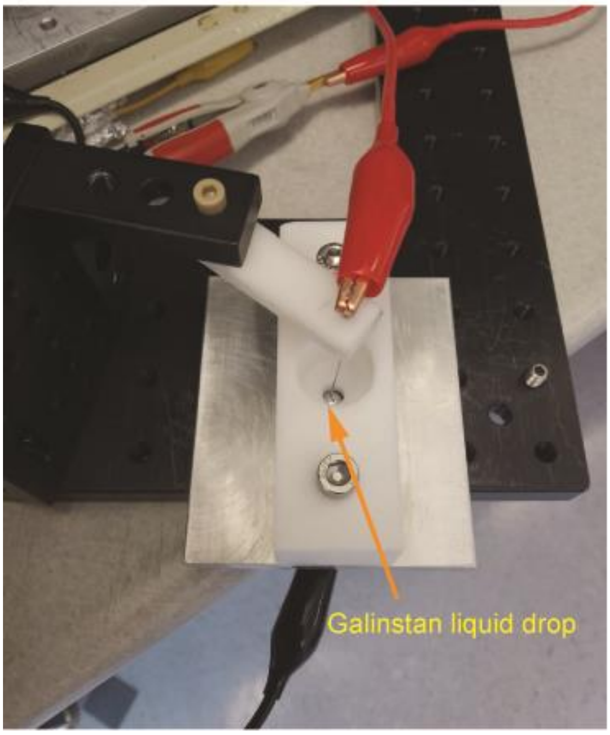

Figure S60. Image of the cell used for capacitance measurements of the photoresists (dielectric constant estimation). The cell ((a), side view; (b), top view) is designed to mount glassy carbon plates spin-coated with samples of SU-8 2002 and NLOF 2035 photoresists. The plates serve as “bottom” electrodes and are electrically connected to the LCR meter (Keysight, E4980AL) via an Al plate. A small quantity (~1 mL) of Galinstan, a liquid metal alloy of gallium, indium, and tin (Ga/In/Sn, 62:22:16 wt %, Thermo Fisher Scientific, United States) was used as the “top” electrode. A platinum wire (0.5 mm diameter) was used to contact the top portion of the Galinstan layer and connect it to the LCR.

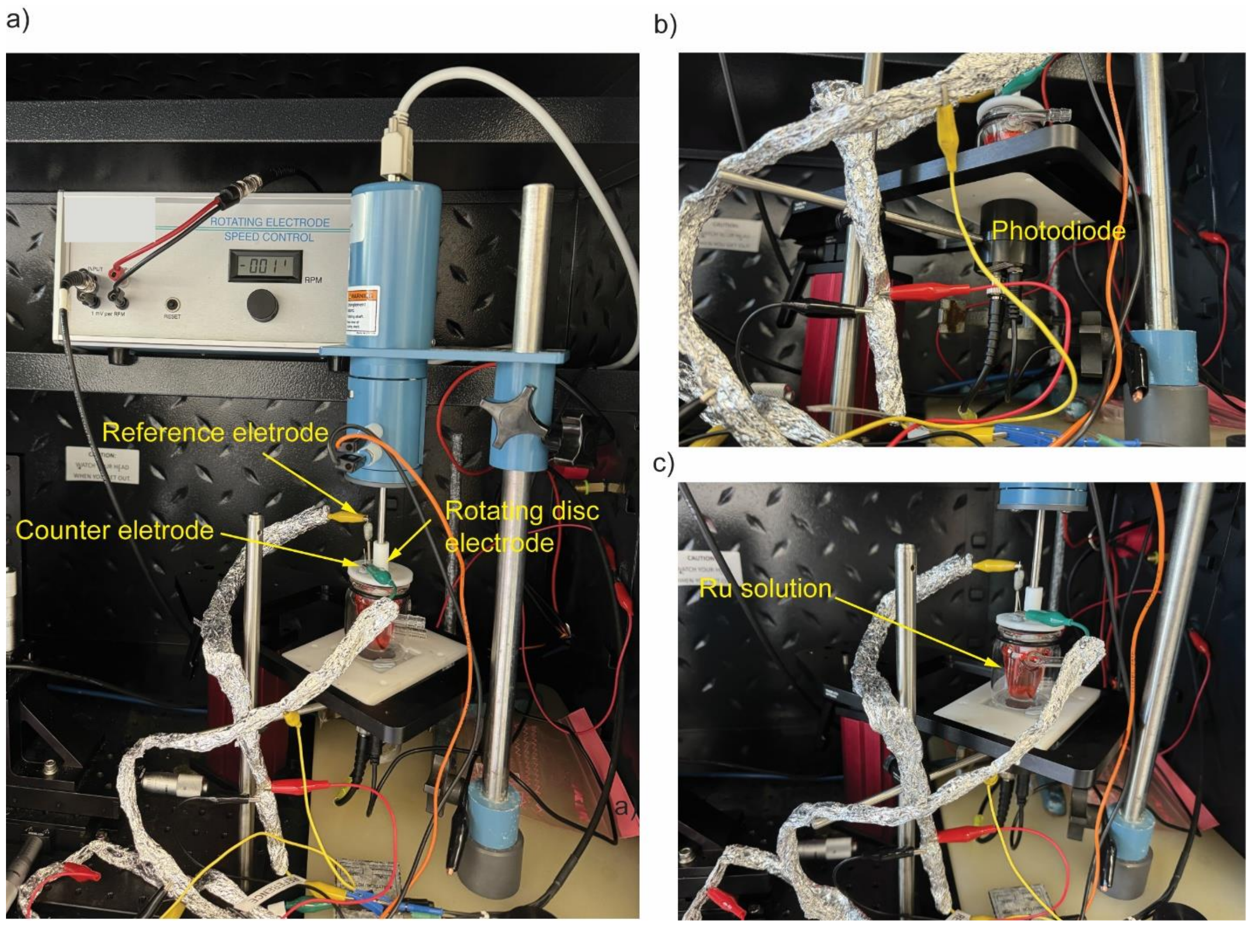

Figure S61. Rotating disc electrode (RDE)/ECL setup. (a) Overview of the setup with text labels indicating main the electrochemical components. A platinum rotating disk electrode (RDE, 5.0 mm disk outer diameter, 12.0 mm PTFE shroud outer diameter, Pine Research Instrumentation, Inc., United States) was used as the working electrode, platinum coil was the counter electrode, and a "leakless" Ag|AgCl in 3.4 M KCl served as reference electrode. (b) The bottom of the transparent three-electrode single-compartment glass cell is facing a photodiode (IPR–T TS2, Inphora Inc., United States) which is connected to a trans-impedance signal amplifier (P–9202–5, Gigahertz-Optik GmbH, Germany). (c) The ECL solution was prepared as described in the experimental section and contained $[Ru(bpy)_3]^{2+}$ (4 mM) and oxalic acid (24 mM) in H_2SO_4 (0.1 M) corrected to pH 6.

Author Contributions

K.W.P. performed most of the experiments and data analysis. H.K. and M.M assisted photolithography process. M.MG. carried out the XPS measurements. N.D., and S.C. supervised the project. K.W.P. and S.C. wrote the manuscript with contributions from all authors.

References

[82] J. Schindelin, I. Arganda-Carreras, E. Frise, V. Kaynig, M. Longair, T. Pietzsch, S. Preibisch, C. Rueden, S. Saalfeld, B. Schmid, J. Y. Tinevez, D. J. White, V. Hartenstein, K. Eliceiri, P. Tomancak, A. Cardona, "Fiji: an open-source platform for biological-image analysis" *Nat. Methods* **2012**, *9*, 676.

[83] S. Liu, C. S. Dutcher, "Measurements of Static and Dynamic Bubble Surface Tension Using a Deformation-Based Microfluidic Tensiometer" *J. Phys. Chem. B* **2021**, *125*, 13916.

[84] H. M. Rodriguez, M. Martyniuk, K. S. Iyer, S. Ciampi, "Insulator-on-Conductor Fouling Amplifies Aqueous Electrolysis Rates" *J. Am. Chem. Soc.* **2024**, *146*.

[85] A. Daerr, A. Mogne, "Pendent_Drop: An ImageJ Plugin to Measure the Surface Tension from an Image of a Pendent Drop" *J. Open Res. Softw.* **2016**, *4*, e3.

[86] E. Huang, A. Skoufis, T. Denning, J. Qi, R. Dagastine, R. Tabor, J. Berry, "OpenDrop: Open-source software for pendant drop tensiometry contact angle measurements" *J. Open Res. Softw.* **2021**, *6*, 2604.

[87] N. Matubayasi, *Surface Tension and Related Thermodynamic Quantities of Aqueous Electrolyte Solutions*, CRC Press, Boca Raton, **2013**.

[88] J. C. W. Corbett, F. McNeil-Watson, R. O. Jack, M. Howarth, "Measuring surface zeta potential using phase analysis light scattering in a simple dip cell arrangement" *Colloids Surf., A* **2012**, *396*, 169.

[89] H. Mateos, A. Valentini, E. Robles, A. Brooker, N. Cioffi, G. Palazzo, "Measurement of the zeta-potential of solid surfaces through Laser Doppler Electrophoresis of colloid tracer in a dip-cell: Survey of the effect of ionic strength, pH, tracer chemical nature and size" *Colloids Surf., A* **2019**, *576*, 82.

[90] C. Hurtado, T. Andreoli, A. P. Le Brun, M. MacGregor, N. Darwish, S. Ciampi, "Galinstan Liquid Metal Electrical Contacts for Monolayer-Modified Silicon Surfaces" *Langmuir* **2024**, *40*, 201.

[91] W. Miao, J.-P. Choi, A. J. Bard, "Electrogenerated Chemiluminescence 69: The Tris(2,2'-bipyridine)ruthenium(II), (Ru(bpy)32+)/Tri-n-propylamine (TPrA) System RevisitedA New Route Involving TPrA•+ Cation Radicals" *J. Am. Chem. Soc.* **2002**, *124*, 14478.
